# New Polyphenols from a Deep Sea *Spiromastix* sp. Fungus, and Their Antibacterial Activities

**DOI:** 10.3390/md13042526

**Published:** 2015-04-22

**Authors:** Siwen Niu, Dong Liu, Peter Proksch, Zongze Shao, Wenhan Lin

**Affiliations:** 1 College of Marine Life, Ocean University of China, Qingdao 266003, China; E-Mails: niusi123@126.com (S.N.); shaozz@163.com (Z.S.); 2 State Key Laboratory of Natural and Biomimetic Drugs, Peking University, Beijing 100191, China; E-Mail: liudong_1982@126.com; 3 Institute für Pharmazeutische Biologie und Biotechnologie, Heinrich-Heine-Universität Düsseldorf, Universitätsstr. 1, Geb.26.23, 40225 Düsseldorf, Germany; E-Mail: proksch@uni-duesseldor.de; 4 Key Laboratory of Marine Biogenetic Resources, Third Institute of Oceanography, SOA, Xiamen 361005, China

**Keywords:** fungus from deep sea, *Spiromastix* sp., spiromastols A–K, polyphenols, antibacterial activities

## Abstract

Eleven new polyphenols namely spiromastols A–K (**1**–**11**) were isolated from the fermentation broth of a deep sea-derived fungus *Spiromastix* sp. MCCC 3A00308. Their structures were determined by extensive NMR data and mass spectroscopic analysis in association with chemical conversion. The structures are classified as diphenyl ethers, diphenyl esters and isocoumarin derivatives, while the *n*-propyl group in the analogues is rarely found in natural products. Compounds **1**–**3** exhibited potent inhibitory effects against a panel of bacterial strains, including *Xanthomanes vesicatoria*, *Pseudomonas lachrymans*, *Agrobacterium tumefaciens*, *Ralstonia solanacearum*, *Bacillus thuringensis*, *Staphylococcus aureus* and *Bacillus subtilis*, with minimal inhibitory concentration (MIC) values ranging from 0.25 to 4 µg/mL. The structure-activity relationships are discussed, while the polychlorinated analogues **1**–**3** are assumed to be a promising structural model for further development as antibacterial agents.

## 1. Introduction

The deep sea is a vast and relatively untapped source of unique molecular, structural and biological diversity with less than 2% of marine natural products reported in literature [1,2,3]. Life in the deep sea requires its inhabitants to adapt their biochemical machinery to cope with extreme conditions, involving exposure to high hydrostatic pressures, variable temperatures and low oxygen and light. The extremophilic organisms may have the potential to induce primary and secondary metabolic pathways to give rise to structurally unique metabolites. The recent advancements in marine technologies have allowed accessing the deep sea and to detect microbial activities [4,5], while screening of phylogenetically diverse and unique organisms from rare or extreme ecosystems in the deep ocean floor has been used to discover relevant bioactive metabolites. Deep sea derived natural products have emerged as a new frontier in drug discovery and development, leading to the identification of anti-tumor, anti-microtubule, anti-proliferative, photoprotective, antibiotic and anti-fouling compounds in the marine environment [6,7,8,9,10,11,12]. Despite the high scientific and commercial interest in the microbial ecology of these ecosystems, relatively little is known about the diversity of functional taxonomic groups of free-living microbes that occupy these niches as well as their biotechnological potential [13]. Fungi derived from deep water sediments have yielded an array of interesting new metabolites, including indole diketopiperazines, indole and quinazolinone alkaloids, sterigmatocystin derivatives, benzodiazepine alkaloids, polyketides, spiroditerpenoids, sesquiterpene quinones, sorbicillinoids, and trichoderones with strong bioactivities such as cytotoxic, antibiotic and antiviral effects.

**Figure 1:**
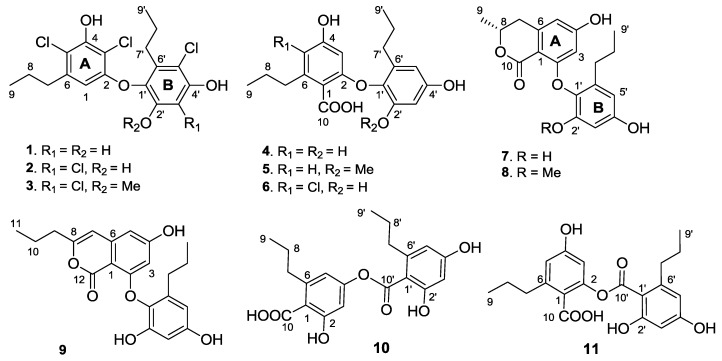
Structures of spiromastols A–K (**1**–**11**).

In the course of our ongoing search for structurally unusual and bioactive secondary metabolites from deep sea derived microorganisms, a *Spiromastix* sp. fungus MCCC 3A00308 isolated from a sediment of South Atlantic at depth of 2869 m was examined chemically. Previously, a number of new depsidone-based spiromastixones A–O with potent antibacterial effects were isolated from the fermentation broth of this specimen [14]. Further examination of the minor components resulted in the isolation of 11 new diaryl derivatives, named spiromastols A–K (**1**–**11**) (Figure 1). Herein, we report the isolation and structure elucidation of the new compounds and their antibacterial activities.

## 2. Results and Discussion

**Figure 2:**
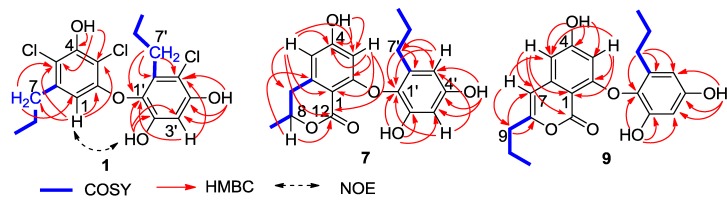
Key 2D NMR correlations of **1**, **7** and **9**.

Spiromastol A (**1**) was isolated as a colorless oil (Figure 1). Its molecular formula was deduced as C_18_H_19_Cl_3_O_4_ on the basis of the HRESIMS (*m/z* 403.0272 [M − H]^−^) and NMR data, requiring eight degrees of unsaturation and containing three chlorine atoms. The IR absorption bands at 3408, 1651 and 1601 cm^−1^ suggested the presence of hydroxy and aromatic functionalities. The ^1^H NMR spectrum displayed three exchangeable protons (δ_H_ 10.00, 9.99 and 9.73), two aromatic singlets at δ_H_ 5.97 (1H, s, H-1) and 6.57 (1H, s, H-3′), and the alkyl protons for four methylene and two methyl groups. The ^13^C NMR spectrum exhibited a total of 18 carbon resonances, including 12 aromatic carbons for two phenyl moieties (rings A and B) and six alkyl carbons for two *n*-propyl groups, which were assigned by the COSY and HMBC correlations (Figure 2). In regard to the substitution of the aromatic ring A, the HMBC interaction of H-1 with a *n*-propyl methylene (δ_C_ 35.6, C-7) allowed to assign the location of the *n*-propyl group vicinal to C-1 (δ_C_ 106.7). Additional HMBC interactions from H-1 to C-2 (δ_C_ 153.1), C-3 (δ_C_ 108.4), and C-5 (δ_C_ 114.8), and a weak correlation with C-4 (δ_C_ 150.3), H_2_-7 (δ_H_ 2.48, m) to C-1, C-5 and C-6 (δ_C_ 139.0), and a phenol proton at δ_H_ 10.00 (brs, OH-4) to C-3, C-4 and C-5 established a 3,5-disubstituted and 2,4-dioxygenated 6-propylphenyl segment, in which C-4 was hydroxylated. Similarly, the substitution of the aromatic ring B was established on the basis of the HMBC relationships. The observation of the HMBC interactions from the aromatic proton H-3′ to C-1′ (δ_C_ 132.7), C-2′ (δ_C_ 149.3), C-4′ (δ_C_ 151.5) and C-5′ (δ_C_ 110.4), the phenol proton at δ_H_ 9.73 (s, OH-2′) to C-1′, C-2′ and C-3′ (δ_C_ 102.7), and the other phenol proton at δ_H_ 9.99 (s, OH-4′) to C-3′, C-4′ and C-5′, in addition to the HMBC interactions of the second *n*-propyl protons, assigned a 5′-substituted and 1′-oxygenated 2′,4′-dihydroxy-6′-propylphenyl ring. Since two aromatic rings covered eight degrees of the molecular unsaturation, the connection of rings A and B was suggested through a C-C bond or an ether bond. The observation of NOE interaction between H-1 and OH-2′ (Figure 2) assumed an ether linkage across C-2 and C-1′. Thus, the quaternary carbons C-3, C-5 and C-5′ were substituted by chlorine atoms.

The molecular formula of spiromastol B (**2**) was determined as C_18_H_18_Cl_4_O_4_ by the HRESIMS (*m/z* 436.9873 [M − H]^−^) and NMR data, indicating the presence of one more chlorine atom and the absence of a proton in comparison with those of **1**. The NMR data of **2** were very similar to those of **1**, with the exception of a quaternary carbon (δ_C_ 109.6, C-3′) of **2** to replace an aromatic methine of **1**. Comparison of the NMR data in association with the HMBC interactions, such as from H_2_-7 (δ_H_ 2.49, m) to C-1 (δ_C_ 106.8), C-5 (δ_C_ 115.4) and C-6 (δ_C_ 139.3), from the phenol proton OH-4 (δ_H_ 10.10, s) to C-3 (δ_C_ 108.9), C-4 (δ_C_ 150.5) and C-5, and from H-1 (δ_H_ 5.97, s) to C-2 (δ_C_ 152.9), C-3, C-4 and C-5, indicated both compounds sharing the partial structure of the aromatic ring A. Thus, the additional chlorine atom was placed in the aromatic ring B. The HMBC relationships of both phenol protons at δ_H_ 9.91 (s, OH-2′) and 9.86 (s, OH-4′) with the quaternary carbon C-3′, clarified the structure of **2** to be a 3′-chlorinated analogue of **1**.

Spiromastol C (**3**) has a molecular formula of C_19_H_20_Cl_4_O_4_ as determined by the HRESIMS data (*m/z* 451.0042 [M − H]^−^), containing a CH_2_ unit more than that of **2**. The similar NMR data with the exception of the presence of methoxy resonances whose protons (δ_H_ 3.83, s) correlated to C-2′ (δ_C_ 150.5) in the HMBC spectrum, clarified compound **3** to be a 2′-methoxy analogue of **2**. The downfield shifted C-1′ (Δ 3.8 ppm), C-2′ (Δ 4.1 ppm) and C-3′ (Δ 6.1 ppm) in comparison with the corresponding carbons of **2** further supported the methoxy substitution.

The molecular formula of spiromastol D (**4**) was determined as C_19_H_22_O_6_ based on the HRESIMS (*m/z* 345.1341 [M − H]^−^) and NMR data. The ^13^C NMR spectrum provided a total of 19 carbon resonances, including 12 aromatic carbons for two phenyl rings, a carboxylic carbon, and six alkyl carbons which were assigned to two *n*-propyl groups based on the COSY and HMBC data. The COSY spectrum displayed two aromatic spin systems of *meta*-couplings between δ_H_ 5.77 (1H, d, *J* = 2.3 Hz, H-3)/6.21(1H, d, *J* = 2.3 Hz, H-5) for ring A, and δ_H_ 6.27 (1H, d, *J* = 2.8 Hz, H-3′)/6.11 (1H, d, *J* = 2.8 Hz, H-5′) for ring B. In the aromatic ring A, the HMBC interactions from a phenol proton at δ_H_ 9.45 (s, OH-4) to C-3 (δ_C_ 98.7), C-4 (δ_C_ 158.8) and C-5 (δ_C_ 109.1) indicated C-4 to be hydroxylated. Additional HMBC interactions from H-5 to the methylene carbon at δ_C_ 35.5 and from H-3 and H-5 to C-1 (δ_C_ 115.7) and the carboxylic carbon at δ_C_ 169.5 through ^4^*J*_H-C_ coupling, revealed the position of a *n*-propyl group at C-6 (δ_C_ 141.9) and the carboxylic group at C-1. The oxygenated C-2 (δ_C_ 157.1) was assigned by the ^2^*J*_H-C_ coupling between H-3 and C-2 in the HMBC spectrum. The substitution of the second *n*-propyl group at C-6′ (δ_C_ 137.0) in the aromatic ring B was evident from the HMBC interaction between H-5′ and the methylene carbon at δ_C_ 32.0 (C-7′), while the NOE interactions between a phenol proton at δ_H_ 9.21 (s, OH-2′) and H-3′, and from the other phenol proton at δ_H_ 9.11 (s, OH-4′) to H-3′ and H-5′ assigned a 2′, 4′-dihydroxy-6′-propylphenyl ring. Since C-4 was positioned by a hydroxy group, the connection of the aromatic ring B at C-1′ (δ_C_ 132.6) with the aromatic ring A through an ether bond with C-2 or ester bond with C-10 was suggested. The observation of the carboxylic proton at δ_H_ 12.58 (brs) clarified C-10 to be an acidic group. Thus, the linkage of ring B with ring A though an ether bond across C-2 and C-1′ was assumed.

Spiromastol E (**5**) has a molecular formula of C_20_H_24_O_6_ as determined by the HRESIMS (*m/z* 359.1498 [M − H]^−^) and NMR data, bearing a CH_2_ unit more than that of **4**. Apart from **5** containing an additional methoxy group (δ_H_ 3.66, δ_C_ 56.0), the NMR data of both **5** and **4** were closely similar (Table 1 and Table 2). The methoxy group of **5** was positioned at C-2′ (δ_C_ 153.2) on the basis of the HMBC relationship between the methoxy protons and C-2′. Thus, compound **5** was determined as a 2′-methoxylated analogue of **4**.

**Table 1:** The ^1^H NMR data of spiromastols A–K (**1**–**11**) (δ_H_ ppm, *J* in Hz).

No	1 ^a^	2 ^a^	3 ^b^	4 ^b^	5 ^b^	6 ^c^	7 ^b^	8 ^a^	9 ^b^	10 ^b^	11 ^a^
1	5.97 s	5.97 s	6.00 s								
3				5.77 (d, 2.3)	5.67 (d, 1.8)	6.05 s	5.90 brs	5.81 (d, 1.8)	5.96 (d, 2.2)	6.59 (d, 1.9)	6.55 (d, 1.7)
5				6.21 (d, 2.3)	6.20 (d, 1.8)		6.26 brs	6.26 (d, 1.8)	6.35 (d, 2.2)	6.51 (d, 1.9)	6.47 (d, 1.7)
7	2.48 m	2.49 m	2.51 m	2.51 (t, 6.5)	2.49 m	2.62 (t, 7.7)	2.77 (dd, 10.6, 16.2) 2.87 (dd, 2.7, 16.2)	2.77 (dd, 10.4, 16.0) 2.87 (dd, 2.5, 16.0)	6.32 s	2.61 m	2.65 (t, 7.5)
8	1.40 m	1.39 m	1.41 m	1.56 m	1.56 m	1.55 m	4.50 m	4.51 m		1.55 m	1.55 m
9	0.81 (t, 7.2)	0.81 (t, 7.2)	0.81 (t, 7.3)	0.90 (t, 7.3)	0.90 (t, 7.2)	0.93 (t, 7.4)	1.37 (d, 6.3)	1.37 (d, 6.3)	2.42 (t, 7.3)	0.89 (t, 7.3)	0.89 (t, 7.5)
10									1.63 m		
11									0.95 (t, 7.5)		
3′	6.57 s			6.27 (d, 2.8)	6.39 (d, 2.4)	6.26 (d, 2.7)	6.28 (d, 2.6)	6.42 (d, 2.3)	6.31 (d, 2.6)	6.25 (d, 1.7)	6.24 (d, 1.8)
5′				6.11 (d, 2.8)	6.25 (d, 2.4)	6.09 (d, 2.7)	6.14 (d, 2.6)	6.28 (d, 2.3)	6.15 (d, 2.6)	6.20 (d, 1.7)	6.19 (d, 1.8)
7′	2.46 m	2.43 m	2.48 m	2.28 (t, 7.4)	2.30 (t, 7.6)	2.26 (t, 7.6)	2.27 (t, 7.6)	2.28 m	2.26 (t, 7.6)	2.59 m	2.59 (t, 7.7)
8′	1.39 m	1.38 m	1.40 m	1.40 m	1.40 m	1.38 m	1.45 m	1.43 m	1.46 m	1.55 m	1.56 m
9′	0.82 (t, 7.2)	0.82 (t, 7.5)	0.83 (t, 7.3)	0.79 (t, 7.3)	0.78 (t, 7.4)	0.77 (t, 7.2)	0.78 (t, 7.4)	0.77 (t, 7.5)	0.77 (t, 7.4)	0.90 (t, 7.3)	0.90 (t, 7.3)
1-COOH				12.58 brs	12.37 brs	12.78 brs				12.23 brs	12.20 brs
2-OH										9.87 s	
4-OH	10.00 brs	10.10 s		9.45 s	9.39 brs	10.10 s	10.20 s	10.22 s	10.46 s		9.87 s
2′-OH	9.73 s	9.91 s		9.21 s		9.33 brs	9.27 s		9.37 s	10.14 s	10.14 s
2′-OCH_3_			3.83 s		3.66 s			3.65 s			
4′-OH	9.99 s	9.86 s		9.11 brs	9.34 brs	9.12 s	9.12 s	9.37 s	9.18 s	9.87 s	9.88 s

^a^ recorded in DMSO-*d*_6_ at 500 MHz; ^b^ recorded in DMSO-*d*_6_ at 400 MHz; ^c^ recorded in DMSO-*d*_6_ at 600 MHz.

**Table 2:** ^13^C NMR data of spiromastols A–K (**1**–**11**) (δ_C_ ppm).

No	1 ^a^	2 ^a^	3 ^b^	4 ^b^	5 ^b^	6 ^c^	7 ^b^	8 ^a^	9 ^b^	10 ^b^	11 ^a^
1	106.7 CH	106.8 CH	106.8 CH	115.7 C	115.9 C	116.4 C	105.0 C	104.7 C	100.8 C	113.6 CH	118.8 C
2	153.1 C	152.9 C	152.5 C	157.1 C	156.9 C	153.9 C	162.4 C	162.4 C	158.3 C	157.4 C	158.3 C
3	108.4 C	108.9 C	109.0 C	98.7 CH	98.3 CH	99.3 CH	100.6 CH	100.2 CH	100.7 CH	107.5 CH	107.5 CH
4	150.3 C	150.5 C	150.6 C	158.8 C	158.7 C	154.2 C	162.8 C	162.8 C	164.2 C	152.2 C	152.2 C
5	114.8 C	115.4 C	115.6 C	109.1 CH	108.9 CH	112.0 C	107.6 CH	107.7 CH	103.9 CH	119.1 C	113.5 CH
6	139.0 C	139.3 C	139.5 C	141.9 C	141.7 C	137.9 C	144.1 C	144.2 C	141.9 C	143.1 C	143.5 C
7	35.6 CH_2_	35.5 CH_2_	35.5 CH_2_	35.5 CH_2_	35.5 CH_2_	33.2 CH_2_	35.8 CH_2_	35.8 CH_2_	103.1 CH	35.9 CH_2_	36.1 CH_2_
8	22.8 CH_2_	22.8 CH_2_	22.8 CH_2_	24.3 CH_2_	24.2 CH_2_	22.6 CH_2_	73.5 CH	73.5 CH	158.1 C	24.4 CH_2_	24.4 CH_2_
9	13.8 CH_3_	13.8 CH_3_	13.8 CH_3_	14.2 CH_3_	14.4 CH_3_	14.2 CH_3_	20.9 CH_3_	20.9 CH_3_	34.8 CH_2_	14.3 CH_3_	14.3 CH_3_
10				169.5 C	169.2 C	168.4 C	168.5 C	168.5 C	20.1 CH_2_	170.2 C	170.4 C
11									13.8 CH_3_		
12									162.8 C		
1′	132.7 C	134.2 C	138.0 C	132.6 C	133.2 C	131.8 C	132.6 C	133.1 C	132.2 C	110.0 C	110.0 C
2′	149.3 C	146.4 C	150.5 C	150.8 C	153.2 C	150.3 C	150.4 C	152.7 C	150.3 C	159.0 C	159.1 C
3′	102.7 CH	109.6 C	115.7 C	102.0 CH	99.0 CH	101.6 CH	102.1 CH	99.1 CH	102.2 CH	101.0 CH	101.0 CH
4′	151.5 C	148.0 C	147.0 C	155.2 C	155.5 C	154.9 C	155.3 C	155.7 C	155.4 C	160.9 C	160.9 C
5′	110.4 C	112.8 C	118.8 C	107.4 CH	108.3 CH	106.9 CH	107.4 CH	108.3 CH	107.5 CH	108.9 CH	108.9 CH
6′	134.6 C	132.7 C	133.5 C	137.0 C	137.0 C	136.4 C	136.5 C	136.8 C	136.3 C	144.2 C	144.2 C
7′	30.1 CH_2_	30.1 CH_2_	30.1 CH_2_	32.0 CH_2_	31.8 CH_2_	31.5 CH_2_	32.2 CH_2_	32.0 CH_2_	32.2 CH_2_	36.3 CH_2_	36.4 CH_2_
8′	22.3 CH_2_	22.1` CH_2_	22.1 CH_2_	23.5 CH_2_	23.4 CH_2_	23.0 CH_2_	23.1 CH_2_	23.0 CH_2_	23.0 CH_2_	24.6 CH_2_	24.6 CH_2_
9′	14.4 CH_3_	14.3 CH_3_	14.3 CH_3_	14.4 CH_3_	14.1 CH_3_	13.7 CH_3_	14.3 CH_3_	14.3 CH_3_	14.3 CH_3_	14.4 CH_3_	14.4 CH_3_
10′										167.3 C	167.4 C
2′-OCH_3_			60.8 CH_3_		56.0 CH_3_			56.0 CH_3_			

^a^ recorded in DMSO-*d*_6_ at 125 MHz; ^b^ recorded in DMSO-*d*_6_ at 100 MHz; ^c^ recorded in DMSO-*d*_6_ at 150 MHz.

The molecular formula of spiromastol F (**6**) was determined as C_19_H_21_ClO_6_ by the HRESIMS (*m/z* 379.0951 [M − H]^−^) and NMR data, with one chlorine atom more than that of **4**. Comparison of the NMR data revealed that both compounds had the same partial structure of the aromatic ring B, whereas a quaternary carbon at δ_C_ 112.0 (C-5) in the aromatic ring A of **6** was recognized to replace the aromatic methine C-5 of **4**. This finding reflected C-5 of **6** to be substituted by a chlorine atom. This assignment was supported by the HMBC correlations from H_2_-7 and OH-4′ (δ_H_ 10.10, s) to C-5, in association with the similar NMR data and HMBC relationships of the remaining resonances.

**Figure 3:**
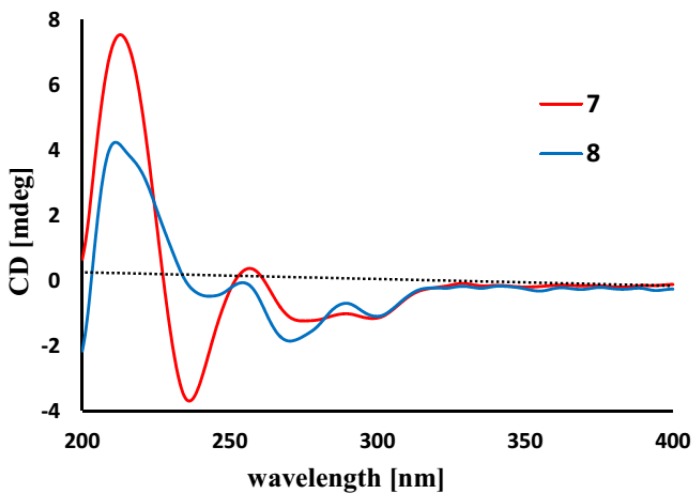
Circular dichroism (CD) curves of **7** and **8**.

Spiromastol G (**7**) has a molecular formula of C_19_H_20_O_6_, as determined by the HRESIMS (*m/z* 343.1176 [M − H]^−^) and NMR data, requiring ten degrees of unsaturation. The NMR data of **7** regarding the aromatic ring B were compatible to those of **4**, indicating that both compounds share the same partial structure of ring B. The distinction was attributed to the substitution of the propyl group at C-6 of the aromatic ring A, in which the methyl protons (δ_H_ 1.37, d, *J* = 6.3 Hz, H_3_-9) showed a COSY correlation with an oxymethine proton (δ_H_ 4.50, m, H-8) and the HMBC interactions with C-8 (δ_C_ 73.5) and C-7 (δ_C_ 35.8). These facts indicated C-8 to be substituted by an oxygen atom. Additional HMBC interaction between H-8 and the carbonyl carbon at δ_C_ 168.5 (C-10) allowed the formation of a δ-lactone. Thus, ring A was assigned as a 8-methyldihydroisocoumarine unit. The HMBC interactions of phenol protons at δ_H_ 10.20 (1H, s, OH-4), 9.27 (1H, s, OH-2′) and 9.12 (1H, s, OH-4′) with the aromatic carbons allowed the assignment of C-4 (δ_C_ 162.8), C-2′ (δ_C_ 150.4) and C-4′ (δ_C_ 155.3) to be hydroxylated. Additional HMBC relationships from the aromatic protons to the aromatic carbons (Figure 2) assigned C-2 (δ_C_ 162.4) and C-1′ (δ_C_ 132.6) to be substituted by oxygen atoms. The absence of phenol protons for OH-2 and OH-1′ conducted the connection of rings A and B through an ether bond across C-2/C-1′. Based on the helicity rule of the chiral benzoic ester chromophore [15,16], the negative circular dichroism (CD) effect (Figure 3) at 273 nm (Δε-1.23) for the n-π* transition reflected 8*R* configuration.

Spiromastol H (**8**) has a molecular formula of C_20_H_22_O_6_ as provided by the HRESIMS (*m/z* 357.1338 [M − H]^−^) and NMR data, containing a CH_2_ unit more than that of **7**. The NMR data of both **8** and **7** were very similar, except for **8** presenting an additional methoxy group (δ_H_ 3.65, δ_C_ 56.0). These findings indicated **8** to be a methoxylated analogue of **7**. The HMBC relationship between the methoxy protons and C-2′ (δ_C_ 152.7) determined **8** as a 2′-methoxylated analogue of **7**. The similar CD effects [Δε-1.84 (272)] (Figure 3) revealed the same configuration at C-8 of both compounds.

The molecular formula of spiromastol I (**9**) was established as C_21_H_22_O_6_ based on the HRESIMS (*m/z* 369.1339 [M − H]^−^) and NMR data, requiring 11 degrees of unsaturation. Comparison of the NMR data resulted in the partial structure regarding the aromatic ring B to be the same as that of **7**. Analysis of 1D and 2D NMR (COSY, HMQC and HMBC) data disclosed an isocoumarin core, based on the presence of *meta*-spin system at H-3 (δ_H_ 5.96, d, *J* = 2.2 Hz) and H-5 (δ_H_ 6.35, d, *J* = 2.2 Hz), in association with the HMBC interactions from H-5 to C-1 (δ_C_ 100.8), C-3 (δ_C_ 100.7), C-4 (δ_C_ 164.2), C-7 (δ_C_ 103.1) and C-12 (δ_C_ 162.8), H-3 to C-1, C-2 (δ_C_ 158.3), C-4, C-5 (δ_C_ 103.9) and C-12, H-7 (δ_H_ 6.32, s) to C-1, C-5, C-6 (δ_C_ 141.9), and C-9 (δ_C_34.8), as well as a phenol proton at δ_H_ 10.46 (s, OH-4) to C-3, C-4 and C-5. These findings ascertained C-4 to be hydroxylated, while C-2 was substituted by an oxygen atom. The COSY correlations afforded an additional *n*-propyl group, whose methylene protons at δ_H_ 2.42 (2H, t, *J* = 7.3 Hz, H_2_-9) showed the HMBC correlations with C-8 and C-7, indicating the *n*-propyl group to be positioned at C-8. Since the phenol protons OH-4, OH-2′ (δ_H_ 9.36, s) and OH-4′ (δ_H_ 9.18, s) were defined, the only possibility for the connection of isocoumarin moiety with the aromatic ring B through a C-2/C-1′ ether bond was determined.

**Figure 4:**
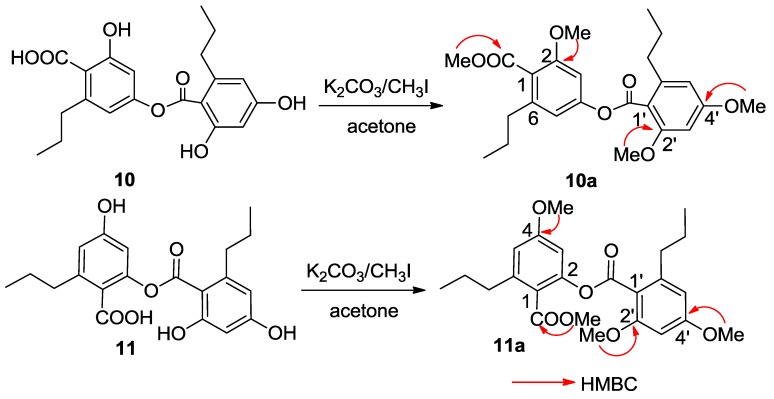
Methylation of **10** and **11**.

Spiromastol J (**10**) had a molecular formula of C_20_H_22_O_7_ as determined by the HRESIMS (*m/z* 373.1287 [M − H]^−^) and NMR data. Analysis of the ^1^H and ^13^C NMR data revealed the presence of two aromatic rings, two *n*-propyl groups and two carboxylic carbons, while the HMQC spectrum assigned the protons and their associated carbons. In the COSY spectrum, two spin systems for *meta*-coupling aromatic protons between δ_H_ 6.59 (d, *J* = 1.9 Hz, H-3)/6.51 (d, *J* = 1.9 Hz, H-5) and δ_H_ 6.25 (d, *J* = 1.7 Hz, H-3′)/6.20 (d, *J* = 1.7 Hz, H-5′) were observed. The substitution of the *n*-propyl groups at C-6 (δ_C_ 143.1) and C-6′ (δ_C_ 144.2), respectively, was evident from the HMBC interactions between H-5/C-7 (δ_C_ 35.9) and H-5′/C-7′ (δ_C_ 36.3). A phenol proton at δ_H_ 10.14 (s) showed the HMBC correlations with C-1′ (δ_C_ 110.0), C-2′ (δ_C_ 159.0) and C-3′ (δ_C_ 101.0) assigned C-2′ to be hydroxylated. Although the absence of the HMBC interaction for remaining phenol protons due to their broad signals, the HMBC correlations of the aromatic protons enabled to assign C-2 (δ_C_ 157.4) and C-4 (δ_C_ 152.2) of the aromatic ring A, and C-4′ (δ_C_ 160.9) of the aromatic ring B to be oxygenated. The ^4^*J*_H-C_ correlations observed from H-3 and H-5 to the carboxylic carbon at δ_C_ 170.2 (C-10) and from H-3′ and H-5′ to δ_C_ 167.3 (C-10′) conducted the carboxylic groups to be located at C-1 and C-1′, respectively. In order to establish the connection of both aromatic rings A and B, compound **10** was methylated [17] to form an analogue **10a** (Figure 4), which displayed four methoxy resonances in the ^1^H NMR spectrum. The HMBC interactions of **10a** between δ_H_ 3.92 (3H, s, OMe)/δ_C_ 168.5 (s, C-10), δ_H_ 3.85 (3H, s, OMe)/δ_C_ 157.3 (s, C-2), δ_H_ 3.86 (3H, s, OMe)/δ_C_ 158.6 (s, C-2′), and δ_H_ 3.89 (3H, s, OMe)/δ_C_ 161.9 (s, C-4′), clarified an ester bond formed across C-4 and C-10′.

Spiromastol K (**11**) has the same molecular formula as that of **10** as determined by the HRESIMS (*m/z* 373.1281 [M − H]^−^) data. The NMR data of both **11** and **10** were virtually similar. The presence of the phenol protons OH-2′ (δ_H_ 10.14, s) and OH-4′ (δ_H_ 9.88, s) in addition to their HMBC correlations with the aromatic carbons resulted in the partial structure regarding the aromatic ring B of **11** to be the same as that of **10**. A phenol proton at δ_H_ 9.87 (s) in ring A showing the HMBC interactions with C-3 (δ_C_ 107.5), C-4 (δ_C_ 152.2) and C-5 (δ_C_ 113.5) revealed C-4 to be hydroxylated. Thus, the connection of aromatic rings B with A through an ester bond across C-10′ (δ_C_ 167.4) and C-2 (δ_C_ 158.3) was assumed. The HMBC interactions of the methylated analogue **11a** (Figure 4) further supported the structural assignment.

Spiromastols (**1**–**11**) were tested against a panel of bacterial strains, including *Xanthomanes vesicatoria* ATCC 11633, *Pseudomonas lachrymans* ATCC11921, *Agrobacterium tumefaciens* ATCC11158, *Ralstonia solanacearum* ATCC11696, *Bacillus thuringensis* ATCC 10792, *Staphylococcus aureus* ATCC 25923 and *Bacillus subtilis* CMCC 63501. As shown in Table 3, compounds **1**–**3** exhibited potent antibacterial activities against all strains of bacteria with minimal inhibitory concentration (MIC) values ranging from 0.25 to 4 µg/mL, while compounds **9–11** showed moderate inhibition with MIC values in the range of 8–64 µg/mL. However, no inhibition was observed for compounds **4–8**. Analysis of the structure-activity relationships of spiromastols revealed that antibacterial activities depended on the substitution in rings A and B. The dichlorinated ring A (**1**–**3**) increased the inhibitory effects against bacteria, while the 2′-methoxylated analogue **3** showed more potent effect than that with a 2′-hydroxy group (**2**). The analogues with a carboxylic acid at C-1 (**4**–**6**) dramatically decreased the antibacterial activity. Comparison of the data of **7**–**9** (Table 3) revealed that isocoumarin **9** showed stronger inhibition than those with dihydroisocoumarin scaffold (**7**–**8**). The analogues with an ester bond connecting rings A and B (**10**–**11**) showed stronger effects than these with an ether bond (**4**–**6**). These data may help to design or modify new analogues with potential antibacterial effects.

**Table 3:** Antibacterial activities of compounds **1**–**11**.

	MIC (µg/mL)						
Compound	*Staphylococcus aureus* ATCC 25923	*Bacillussubtilis* CMCC 63501	*Bacillus thuringensis* ATCC 10792	*Ralstonia solanacearum* ATCC 11696	*Xanthomanes vesicatoria* ATCC 11633	*Agrobacterium tumefaciens* ATCC11158	*Pseudomonas lachrymans* ATCC11921
**1**	0.5	0.5	0.25	0.5	0.5	0.25	0.5
**2**	4	4	4	4	4	2	4
**3**	0.25	0.5	0.5	0.5	0.5	0.5	0.5
**4**	>128	>128	>128	>128	>128	>128	>128
**5**	>128	>128	>128	>128	>128	>128	>128
**6**	>128	>128	>128	>128	>128	>128	>128
**7**	>128	>128	>128	>128	>128	>128	>128
**8**	>128	>128	>128	>128	>128	>128	>128
**9**	8	8	8	8	16	8	16
**10**	32	32	32	32	64	32	32
**11**	32	32	32	32	64	32	32
**CP** ^a^	1	1	1	2	2	2	2

CP ^a^: chloroamphenicol, positive control.

## 3. Experimental Section

### 3.1. General Experimental Procedures

Optical rotations were measured on a Rudolph IV Autopol automatic polarimeter at 25 °C. UV spectra were measured on a Cary 300 spectrometer. IR spectra were measured on a Thermo Nicolet Nexus 470 FT-IR spectrometer. CD spectra were measured on a JASCO J-810 spectropolarimeter. ^1^H, ^13^C, and 2D NMR spectra were recorded on a Bruker Advance 400, 500, and 600 NMR spectrometers, respectively. Chemical shifts are expressed in δ (ppm) referenced to the solvent peaks at δ_H_ 2.50 and δ_C_ 39.5 for DMSO-*d*_6_, and δ_H_ 7.26 and δ_C_ 77.2 for CDCl_3_, respectively, and coupling constants are in Hz. HRESIMS spectra were obtained from Xevo G2 Q-TOF mass spectrometer. Materials for column chromatography (CC) involved silica gel (100–200 and 200–300 mesh, Qingdao Marine Chemistry Co. Ltd., Qingdao, China), ODS gel (50 μm, YMC, Japan) and Sephadex LH-20 (18–110 μm, Amersham Pharmacia Biotech AB, Uppsala, Sweden). Precoated silica gel plates (Merck, Kieselgel 60 F254, 0.25 mm) were used for TLC analysis. HPLC chromatography was performed on a Waters e2695 separation Module coupled with a Waters 2998 photodiode array detector and a semi-preparative reversed-phased column (YMC-packed C_18_, 5 μm, 250 mm × 10 mm) was used for purification.

### 3.2. Fungal Material and Fermentation

The fungal *Spiromastix* sp. MCCC 3A00308 was isolated from a deep ocean sediment, which was collected with TV-multicore in June 2011 from the South Atlantic Ocean at site S015-TVMC06 (GPS 13.75° W, 15.17° S) at a depth of 2869 m during the Comra 22nd oceanic cruise Leg 5. The fungus was identified as *Spiromastix* genus by ITS gene sequence analysis (GeneBank accession number KJ010057). The strain MCCC 3A00308 was deposited in the Marine Culture Collection Center (MCCC), Third Institute of Oceanography, State Oceanic Administration, Xiamen, China. The fungus *Spiromastix* sp. MCCC 3A00308 was cultured on PDA slants at 25 °C for 10 days. The fermentation was carried out in Erlenmeyer flasks (50 × 500 mL), each containing 100 g of rice, to which distilled H_2_O (140 mL) was added. The contents were soaked overnight before autoclaving at 15 psi for 30 min. After cooling to about 30 °C, each flask was inoculated with 5 mL of the spore inoculum and incubated at 25 °C for 50 days.

### 3.3. Extraction and Isolation

The fermentation broth of *Spiromastix* sp. MCCC 3A00308 fungus was extracted three times with ethyl acetate (3 × 10 L). The organic extracts were evaporated under vacuum to afford crude extracts (58.4 g). The crude extract was subjected to silica gel vacuum liquid chromatography (VLC) eluted with petroleum ether–Me_2_CO (50:1–1:1) to afford 10 fractions (FA–FJ). FG (6.1 g) was purified through an ODS column eluted with MeOH-H_2_O (1:1–1:0) to give eleven sub-fractions (SFG1–SFG11). SFG9 (165 mg) was separated sequentially on ODS column eluted with MeOH–H_2_O (1:1–1:0), and semi-preparative HPLC with a mobile phase of MeCN-H_2_O (4:1) to yield compound **3** (3.8 mg). FH (8.2 g) was separated by ODS chromatography eluted with MeOH–H_2_O (1:1–1:0) to obtain ten sub-fractions (SFH1–SFH10). SFH5 (423 mg) was purified by silica gel column eluted with petroleum ether-acetone (10:1) and then by semi-preparative HPLC with a mobile phase of MeCN–H_2_O (11:9) to obtain **6** (1.2 mg), while compounds **9** (6.5 mg), **11** (4.1 mg), and **10** (18.7 mg) were separated from SFH4 (120 mg) by the same protocol as for SFH5. SFH6 (72 mg) was subjected to a Sephadex LH-20 column eluting with MeOH to afford compound **1** (4.6 mg). SFH7 (1.1 g) was purified by an ODS column eluted with MeOH–H_2_O (2:3–1:0), and then by semi-preparative HPLC with a mobile phase of MeCN-H_2_O (33:17) to obtain **2** (2.0 mg). FI (2.7 g) was separated by ODS chromatography eluted with MeOH-H_2_O (1:1–1:0) to obtain twenty-eight sub-fractions (SFI1–SFI28). SFI20 (168 mg) was separated by a Sephadex LH-20 column eluted with MeOH to collect three fractions (SFI20-1 to SFI20-3). The semi-preparative HPLC separation of SFI20-1 (28 mg) with a mobile phase of MeCN-H_2_O (11:9) to yield compounds **5** (3.5 mg) and **8** (1.6 mg), while compounds **7** (5.0 mg) and **4** (22.3 mg) were obtained from SFI20-2 (46 mg) by the same protocol as for SFI20-1.

Spiromastol A (**1**): Colorless oil; UV (MeOH) λ_max_ (logε) 215 (4.48), 288 (3.71); IR (KBr) ν_max_ 3408, 2962, 2871, 1651, 1601, 1459, 1242, 1154 cm^−1^; ^1^H and ^13^C NMR data, see Table 1 and Table 2; HRESIMS *m/z* 403.0272 [M − H]^−^ (calcd for C_18_H_18_O_4_Cl_3_, 403.0271).

Spiromastol B (**2**): Colorless oil; UV (MeOH) λ_max_ (logε) 213 (4.42), 288 (3.54); IR (KBr) ν_max_ 3450, 2961, 2930, 2870, 1716, 1580, 1442, 1335, 1227, 1180 cm^−1^; ^1^H and ^13^C NMR data, see Table 1 and Table 2; HRESIMS *m/z* 436.9873 [M − H]^−^ (calcd for C_18_H_17_O_4_Cl_4_, 436.9881).

Spiromastol C (**3**): Colorless oil; UV (MeOH) λ_max_ (logε) 215 (4.59), 288 (3.46); IR (KBr) ν_max_ 3360, 2960, 2933, 2870, 1655, 1581, 1461, 1419, 1313, 1246, 1198 cm^−1^; ^1^H and ^13^C NMR data, see Table 1 and Table 2; HRESIMS *m/z* 451.0042 [M − H]^−^ (calcd for C_19_H_19_O_4_Cl_4_, 451.0037).

Spiromastol D (**4**): Colorless oil; UV (MeOH) λ_max_ (logε) 213 (4.37), 282 (3.67); IR (KBr) ν_max_ 3276, 2961, 2930, 2870, 1705, 1605, 1459, 1317, 1239, 1193 cm^−1^; ^1^H and ^13^C NMR data, see Table 1 and Table 2; HRESIMS *m/z* 345.1341 [M − H]^−^ (calcd for C_19_H_21_O_6_, 345.1338).

Spiromastol E (**5**): Colorless oil; UV (MeOH) λ_max_ (logε) 208 (4.19), 279 (3.46); IR (KBr) ν_max_ 3281, 2959, 2930, 2870, 1705, 1604, 1458, 1321, 1251, 1195 cm^−1^; ^1^H and ^13^C NMR data, see Table 1 and Table 2; HRESIMS *m/z* 359.1498 [M − H]^−^ (calcd for C_20_H_23_O_6_, 359.1495).

Spiromastol F (**6**): Colorless oil; UV (MeOH) λ_max_ (logε) 215 (4.26), 282 (3.52); IR (KBr) ν_max_ 3306, 2961, 2931, 2871, 1701, 1607, 1458, 1364, 1231, 1196 cm^−1^; ^1^H and ^13^C NMR data, see Table 1 and Table 2; HRESIMS *m/z* 379.0951 [M − H]^−^ (calcd for C_19_H_20_O_6_Cl, 379.0948).

Spiromastol G (**7**): Colorless oil; ${\left[\text{α}\right]}_{\text{D}}^{25}$ −4 (*c* = 0.2, MeOH); UV (MeOH) λ_max_ (logε) 206 (4.27), 227 (4.25), 266 (3.98), 292 (3.64); IR (KBr) ν_max_ 3349, 2960, 2928, 2869, 1676, 1608, 1464, 1340, 1257, 1186, 1158 cm^−1^; CD (MeOH) λ (Δε) 301 (−1.06), 291 (−1.02), 273 (−1.23), 259 (0.30), 238 (−3.47), 215 (7.38). ^1^H and ^13^C NMR data, see Table 1 and Table 2; HRESIMS *m/z* 343.1176 [M − H]^−^ (calcd for C_19_H_19_O_6_, 343.1182).

Spiromastol H (**8**): Colorless oil; ${\left[\text{α}\right]}_{\text{D}}^{25}$ −6 (*c* 0.2, MeOH); UV (MeOH) λ_max_ (logε) 205 (4.16), 226 (4.02), 265 (3.81), 292 (3.51); IR (KBr) ν_max_ 3358, 2961, 2930, 2870, 1688, 1607, 1463, 1337, 1249, 1193 cm^−1^; CD (MeOH) λ (Δε) 301 (−1.06), 291 (−0.75), 272 (−1.84), 257 (−0.11), 243 (−0.47), 213 (4.12). ^1^H and ^13^C NMR data, see Table 1 and Table 2; HRESIMS *m/z* 357.1338 [M − H]^−^ (calcd for C_20_H_21_O_6_, 357.1338).

Spiromastol I (**9**): Colorless oil; UV (MeOH) λ_max_ (logε) 207 (4.26), 245 (4.60), 278 (3.88), 321 (3.64); IR (KBr) ν_max_ 3246, 2960, 2871, 1689, 1597, 1461, 1359 cm^−1^; ^1^H and ^13^C NMR data, see Table 1 and Table 2; HRESIMS *m/z* 369.1339 [M − H]^−^ (calcd for C_21_H_21_O_6_, 369.1338).

Spiromastol J (**10**): Colorless oil; UV (MeOH) λ_max_ (logε) 216 (4.41), 270 (4.13), 301 (3.92); IR (KBr) ν_max_ 3396, 2960, 2872, 1656, 1614, 1451, 1311, 1248, 1192 cm^−1^; ^1^H and ^13^C NMR data, see Table 1 and Table 2; HRESIMS *m/z* 373.1287 [M − H]^−^ (calcd for C_20_H_21_O_7_, 373.1287).

Spiromastol K (**11**): Colorless oil; UV (MeOH) λ_max_ (logε) 216 (4.32), 270 (4.04), 300 (3.85); IR (KBr) ν_max_ 3163, 2959, 2870, 1654, 1591, 1453, 1310, 1248, 1195 cm^−1^; ^1^H and ^13^C NMR data, see Table 1 and Table 2; HRESIMS *m/z* 373.1281 [M − H]^−^ (calcd for C_20_H_21_O_7_, 373.1287).

### 3.4. Methylation

Compound **10** (5.0 mg) was dissolved in anhydrous acetone (900 µL), and then K_2_CO_3_ (3.8 mg) and CH_3_I (650 μL) were added to the solution, which was stirred for 16 h at 0 °C. After filtration, the solution was concentrated *in vacuo*, and the residue was purified on a silica gel column eluting with petroleum ether-Me_2_CO (20: 1) to obtain the methyl ether **10a** (2.9 mg, 58% yield). Compound **11** is submitted to the same protocol as for **10** to derive a methylated product **11a**.

Compound **10a**: ^1^H NMR (400 MHz, CDCl_3_) δ_H_ 6.72 (1H, d, *J* = 1.8, H-3), 6.68 (1H, d, *J* = 1.8, H-5), 6.41 (1H, d, *J* = 2.1, H-5′), 6.39 (1H, d, *J* = 2.1, H-3′), 3.92 (3H, s, OMe-10), 3.89 (3H, s, OMe-2′), 3.86 (3H, s, OMe-4′), 3.85 (3H, s, OMe-2), 2.70 (2H, t, *J* = 7.8, H_2_-7′), 2.58 (2H, t, *J* = 7.9, H_2_-7), 1.72 (2H, m, H_2_-8′), 1.65 (2H, m, H_2_-8), 1.00 (3H, t, *J* = 7.3, H_3_-9′), 0.97 (3H, t, *J* = 7.4, H_3_-9); ^13^C NMR (100 MHz, CDCl_3_) δ_C_ 168.5 (C-10), 166.4 (C-10′), 161.9 (C-4′), 158.6 (C-2′), 157.3 (C-4), 152.5 (C-2), 143.5 (C-6′), 142.4 (C-6), 121.1 (C-5), 115.2 (C-1′), 114.4 (C-1), 106.1 (C-5′), 102.7 (C-3), 96.3 (C-3′), 56.1 (OMe-2′), 56.0 (OMe-2), 55.4 (OMe-4′), 52.2 (OMe-10), 36.1 (C-7′), 35.6 (C-7), 24.6 (C-8′), 24.0 (C-8), 14.1 (C-9′), 14.0 (C-9). ESIMS *m/z* 431.32 [M + H]^+^, 453.26 [M + Na]^+^.

Compound **11a**: ^1^H NMR (400 MHz, CDCl_3_) δ_H_ 6.70 (1H, d, *J* = 2.0, H-3), 6.66 (1H, d, *J* = 2.0, H-5), 6.40 (1H, d, *J* = 2.0, H-5′), 6.38 (1H, d, *J* = 2.0, H-3′), 3.92 (3H, s, OMe-10), 3.87 (3H, s, OMe-2′), 3.86 (3H, s, OMe-4′), 3.82 (3H, s, OMe-4), 2.71 (2H, t, *J* = 7.8, H_2_-7′), 2.56 (2H, t, *J* = 7.9, H_2_-7), 1.71 (2H, m, H_2_-8′), 1.67 (2H, m, H_2_-8), 1.01 (3H, t, *J* = 7.0, H_3_-9′), 0.96 (3H, t, *J* = 7.0, H_3_-9); ^13^C NMR (100 MHz, CDCl_3_) δ_C_ 168.3 (C-10), 166.5 (C-10′), 161.8 (C-4′), 158.5 (C-2′), 157.8 (C-4), 152.3 (C-2), 143.4 (C-6′), 142.5 (C-6), 119.0 (C-5), 115.3 (C-1′), 114.2 (C-1), 106.0 (C-5′), 102.2 (C-3), 96.4 (C-3′), 56.2 (OMe-2′), 56.1 (OMe-4), 55.3 (OMe-4′), 52.1 (OMe-10), 36.0 (C-7′), 35.5 (C-7), 24.7 (C-8′), 24.4 (C-8), 14.0 (C-9′), 14.1 (C-9). ESIMS *m/z* 431.30 [M + H]^+^.

### 3.5. Antibacterial Assay

Spiromastols A–K (**1**–**11**) were tested against a panel of seven bacterial strains, including *Xanthomanes vesicatoria* ATCC 11633, *Pseudomonas lachrymans* ATCC11921, *Agrobacterium tumefaciens* ATCC11158, *Ralstonia solanacearum* ATCC11696, *Bacillus thuringensis* ATCC 10792, *Staphylococcus aureus* ATCC 25923 and *Bacillus subtilis* CMCC 63501, according to previously described methods [14].

## 4. Conclusions

In summary, this work described a group of new polyphenols with diverse scaffolds derived from deep sea derived fungus *Spiromastix* sp., while these findings provided additional evidence to support that the microorganisms from deep sea are a potential source for the discovery of chemical diversity. The potent antibacterial effects of **1**–**3** suggested that these compounds can be used for further lead modification.

## Supplementary Files

Supplementary File 1
