# Supplementary material for: New Polyphenols from a Deep Sea Spiromastix sp. Fungus, and Their Antibacterial Activities
Source: Mar Drugs. 2015 Apr 22;13(4):2526–40. doi: 10.3390/md13042526 (PMC4413224; doi:10.3390/md13042526)
Supplement: Supplementary File 1 [file marinedrugs-13-02526-s001.pdf]

Supplementary Information

Figure S1. IR spectrum of compound **1**.
Figure S2. Negative mode HRESIMS data of compound **1**.
Figure S3. ^{1}H NMR spectrum of compound **1** in DMSO-*d6* (500 MHz).
Figure S4. ^{13}C NMR spectrum of compound **1** in DMSO-d_6 (125 MHz).
Figure S5. HSQC spectrum of compound **1** in DMSO-d_6.
Figure S6. ^{1}H-^{1}H COSY spectrum of compound **1** in DMSO-d_6.
Figure S7. HMBC spectrum of compound **1** in DMSO-d_6.
Figure S7-1. HMBC spectrum of compound **1** in DMSO-d_6 (part amplification).
Figure S8. NOESY spectrum of compound **1** in DMSO-d_6.
Figure S9. IR spectrum of compound **2**.
Figure S10. Negative mode HRESIMS data of compound **2**.
Figure S11. ^{1}H NMR spectrum of compound **2** in DMSO-d_6 (500 MHz).
Figure S12. ^{13}C NMR spectrum of compound **2** in DMSO-d_6 (125 MHz).
Figure S13. HSQC spectrum of compound **2** in DMSO-d_6.
Figure S14. ^{1}H-^{1}H COSY spectrum of compound **2** in DMSO-d_6.
Figure S15. HMBC spectrum of compound **2** in DMSO-d_6.
Figure S16. IR spectrum of compound **3**.
Figure S17. Negative mode HRESIMS data of compound **3**.
Figure S18. ^{1}H NMR spectrum of compound **3** in DMSO-d_6 (400 MHz).
Figure S19. ^{13}C NMR spectrum of compound **3** in DMSO-d_6 (100 MHz).
Figure S20. HSQC spectrum of compound **3** in DMSO-d_6.
Figure S21. ^{1}H-^{1}H COSY spectrum of compound **3** in DMSO-d_6.
Figure S22. HMBC spectrum of compound **3** in DMSO-d_6.
Figure S23. IR spectrum of compound **4**.
Figure S24. Negative mode HRESIMS data of compound **4**.
Figure S25. ^{1}H NMR spectrum of compound **4** in DMSO-d_6 (400 MHz).
Figure S26. ^{13}C NMR spectrum of compound **4** in DMSO-d_6 (100 MHz).
Figure S27. HSQC spectrum of compound **4** in DMSO-d_6.
Figure S28. ^{1}H-^{1}H COSY spectrum of compound **4** in DMSO-d_6.
Figure S29. HMBC spectrum of compound **4** in DMSO-d_6.
Figure S30. NOESY spectrum of compound **4** in DMSO-d_6.
Figure S31. IR spectrum of compound **5**.
Figure S32. Negative mode HRESIMS data of compound **5**.
Figure S33. ^{1}H NMR spectrum of compound **5** in DMSO-d_6 (400 MHz).
Figure S34. ^{13}C NMR spectrum of compound **5** in DMSO-d_6 (100 MHz).
Figure S35. HSQC spectrum of compound **5** in DMSO-d_6.
Figure S36. ^{1}H-^{1}H COSY spectrum of compound **5** in DMSO-d_6.
Figure S37. HMBC spectrum of compound **5** in DMSO-d_6.
Figure S38. IR Spectrum of compound **6**.
Figure S39. Negative mode HRESIMS data of compound **6**.
Figure S40. ^{1}H NMR spectrum of compound **6** in DMSO-d_6 (600 MHz).
Figure S41. ^{13}C NMR spectrum of compound **6** in DMSO-d_6 (150 MHz).
Figure S42. HSQC spectrum of compound **6** in DMSO-d_6.
Figure S43. ^{1}H-^{1}H COSY spectrum of compound **6** in DMSO-d_6.
Figure S44. HMBC spectrum of compound **6** in DMSO-d_6.
Figure S45. IR spectrum of compound **7.**
Figure S46. Negative mode HRESIMS data of compound **7.**

Figure S47. ^{1}H NMR spectrum of compound **7** in DMSO-d_6 (400 MHz).
Figure S48. ^{13}C NMR spectrum of compound **7** in DMSO-d_6 (100 MHz).
Figure S49. HSQC spectrum of compound **7** in DMSO-d_6.
Figure S50. ^{1}H-^{1}H COSY spectrum of compound **7** in DMSO-d_6.
Figure S51. HMBC spectrum of compound **7** in DMSO-d6.
Figure S52. IR spectrum of compound **8**.
Figure S53. Negative mode HRESIMS data of compound **8**.
Figure S54. ^{1}H NMR spectrum of compound **8** in DMSO-d_6 (500 MHz).
Figure S55. ^{13}C NMR spectrum of compound **8** in DMSO-d_6 (125 MHz).
Figure S56. HSQC spectrum of compound **8** in DMSO-d_6.
Figure S57. ^{1}H-^{1}H COSY spectrum of compound **8** in DMSO-d_6.
Figure S58. HMBC spectrum of compound **8** in DMSO-d_6.
Figure S59. IR spectrum of compound **9**.
Figure S60. Negative mode HRESIMS data of compound **9**.
Figure S61. ^{1}H NMR spectrum of compound **9** in DMSO-d_6 (400 MHz).
Figure S62. ^{13}C NMR spectrum of compound **9** in DMSO-d_6 (100 MHz).
Figure S63. HSQC spectrum of compound **9** in DMSO-d_6.
Figure S64. ^{1}H-^{1}H COSY spectrum of compound **9** in DMSO-d_6.
Figure S65. HMBC spectrum of compound **9** in DMSO-d_6.
Figure S66. IR spectrum of compound **10**.
Figure S67. Negative mode HRESIMS data of compound **10**.
Figure S68. ^{1}H NMR spectrum of compound **10** in DMSO-d_6 (500 MHz).
Figure S69. ^{13}C NMR spectrum of compound **10** in DMSO-d_6 (125 MHz).
Figure S70. HSQC spectrum of compound **10** in DMSO-d_6.
Figure S71. ^{1}H-^{1}H COSY spectrum of compound **10** in DMSO-d_6.
Figure S72. HMBC spectrum of compound **10** in DMSO-d_6.
Figure S73. IR spectrum of compound **11**.
Figure S74. Negative mode HRESIMS data of compound **11**.
Figure S75. ^{1}H NMR spectrum of compound **11** in DMSO-d_6 (400 MHz).
Figure S76. ^{13}C NMR spectrum of compound **11** in DMSO-d_6 (100 MHz).
Figure S77. HSQC spectrum of compound **11** in DMSO-d_6.
Figure S78. ^{1}H-^{1}H COSY spectrum of compound **11** in DMSO-d_6.
Figure S79. HMBC spectrum of compound **11** in DMSO-d_6.
Figure S80. Negative mode ESIMS data of compound **10a**.
Figure S81. ^{1}H NMR spectrum of compound **10a** in $CDCl_3$ (400 MHz).
Figure S82. ^{13}C NMR spectrum of compound **10a** in $CDCl_3$ (100 MHz).
Figure S83. HSQC spectrum of compound **10a** in $CDCl_3$.
Figure S84. ^{1}H-^{1}H COSY spectrum of compound **10a** in $CDCl_3$.
Figure S85. HMBC spectrum of compound **10a** in $CDCl_3$.

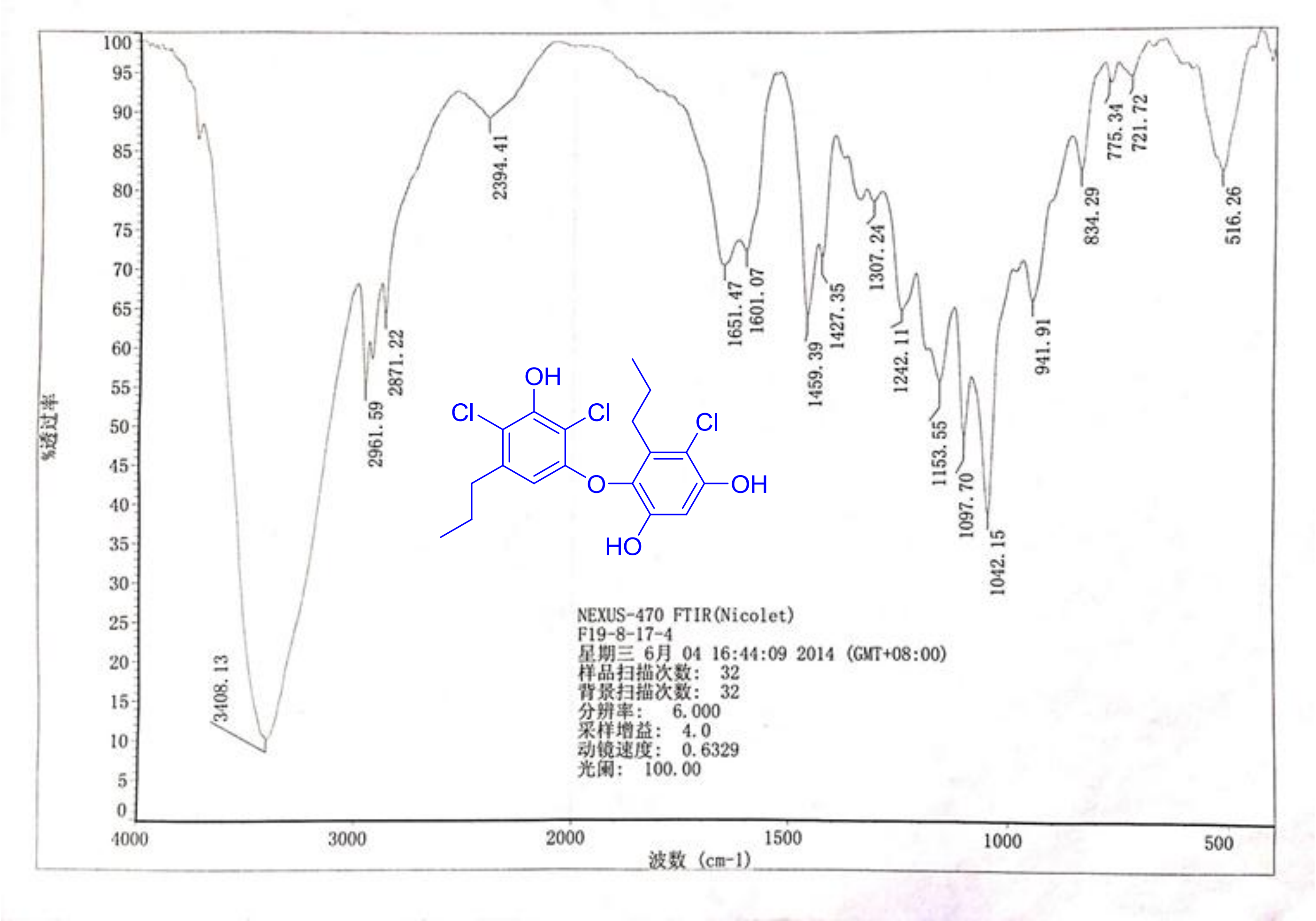

Figure S1. IR spectrum of compound **1**.

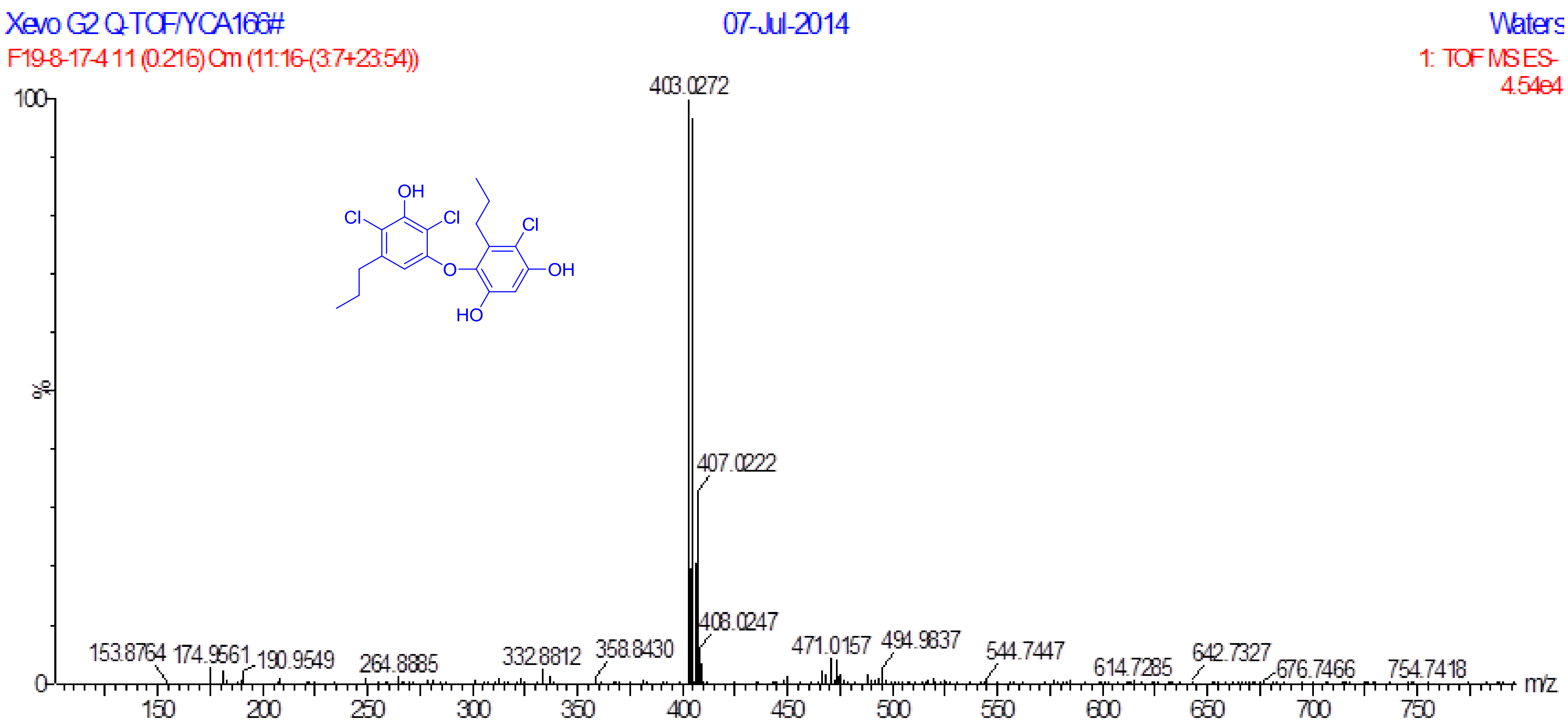

Figure S2. Negative mode HRESIMS data of compound **1**.

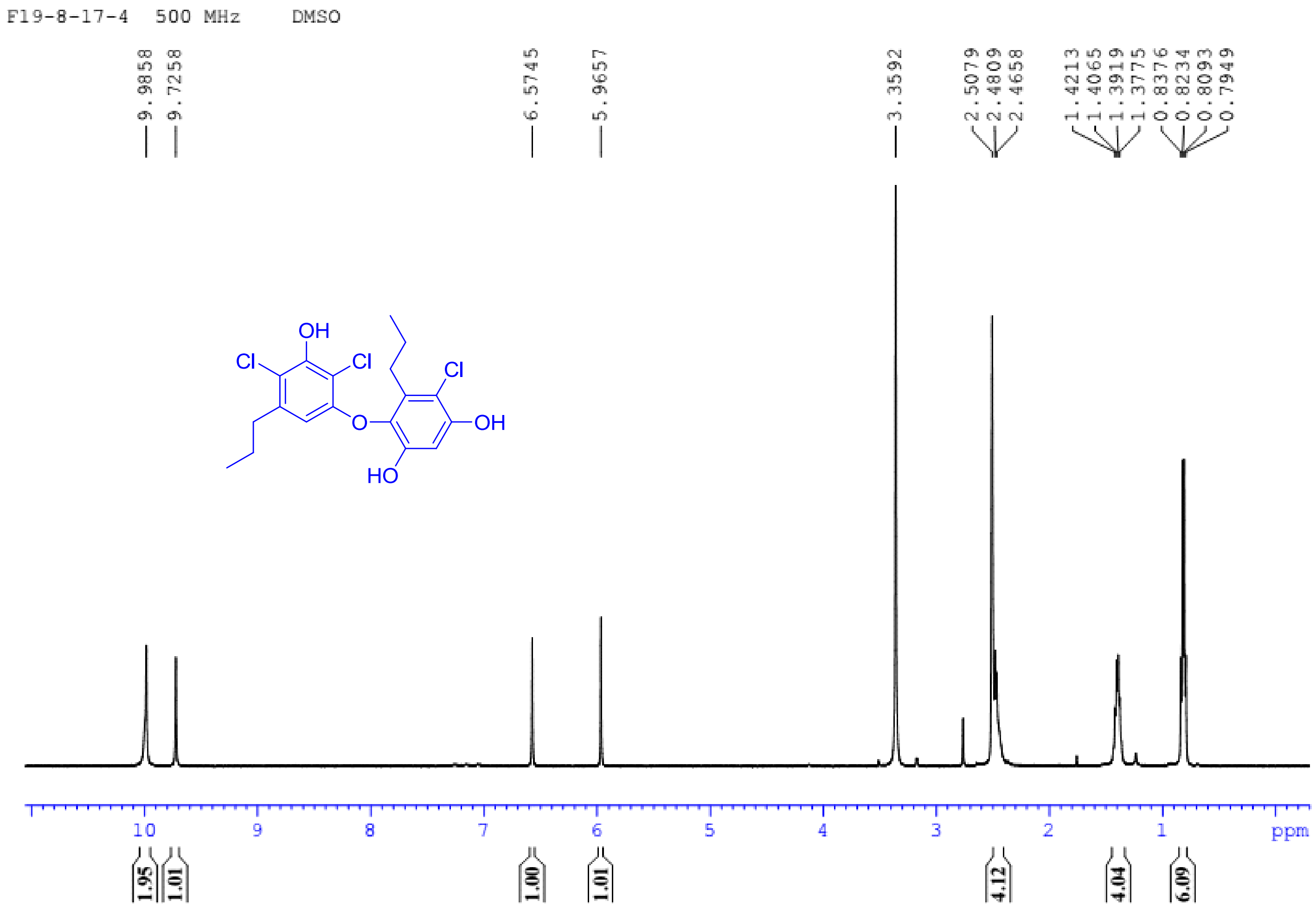

Figure S3. ^{1}H NMR spectrum of compound **1** in DMSO-d6 (500 MHz).

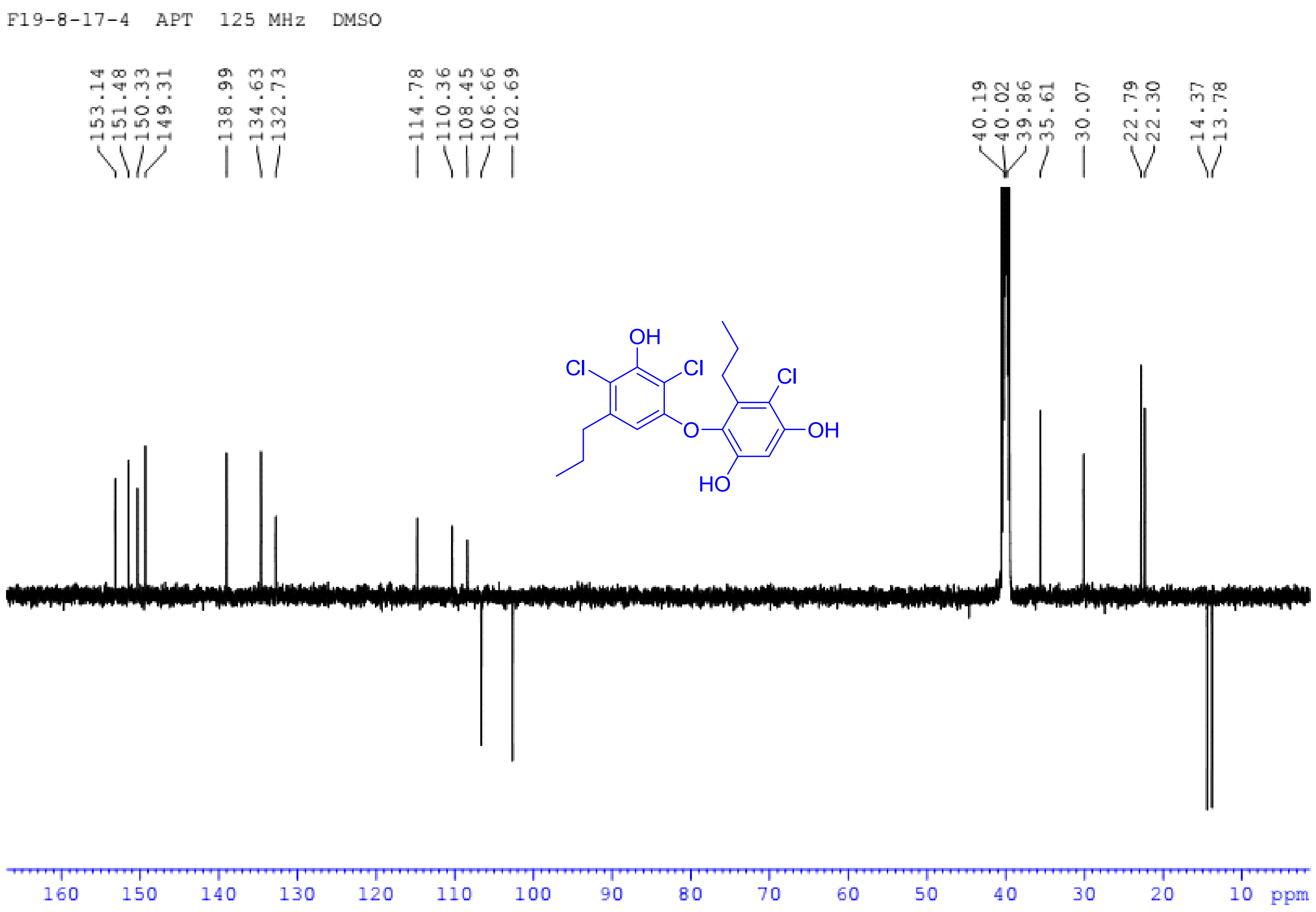

Figure S4. ^{13}C NMR spectrum of compound **1** in DMSO-d6 (125 MHz).

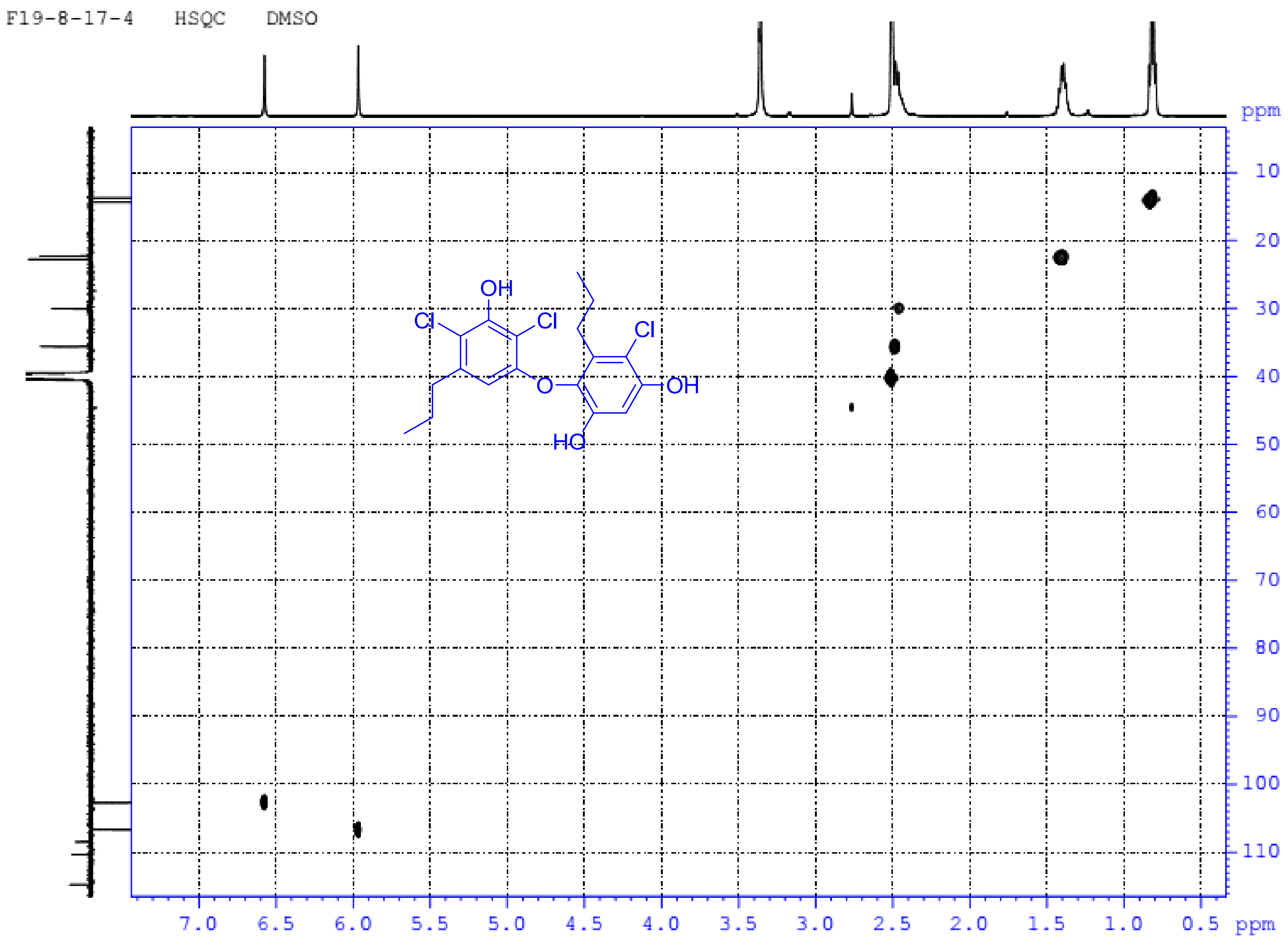

Figure S5. HSQC spectrum of compound **1** in DMSO-d6.

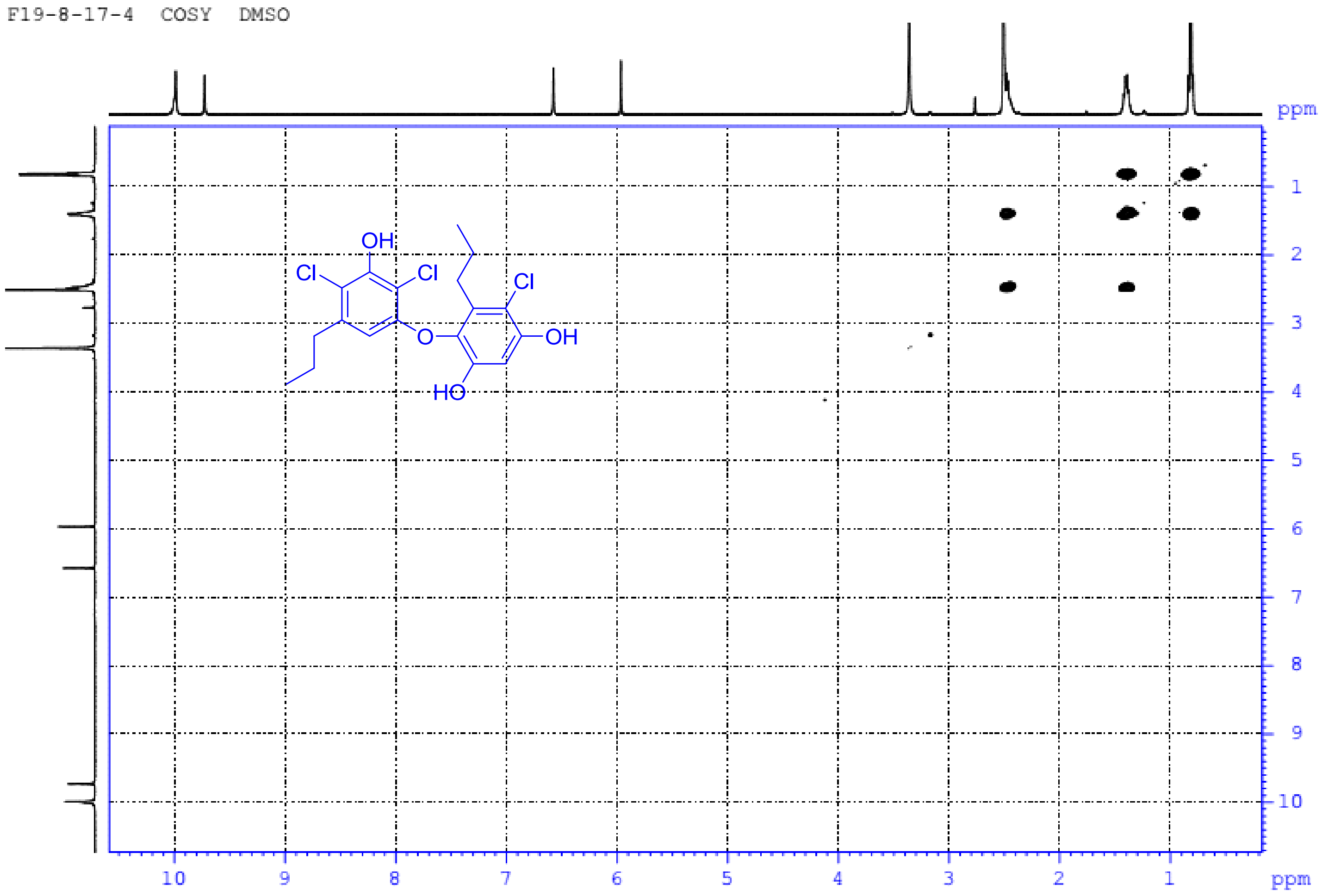

Figure S6. ^{1}H-^{1}H COSY spectrum of compound **1** in DMSO-d6.

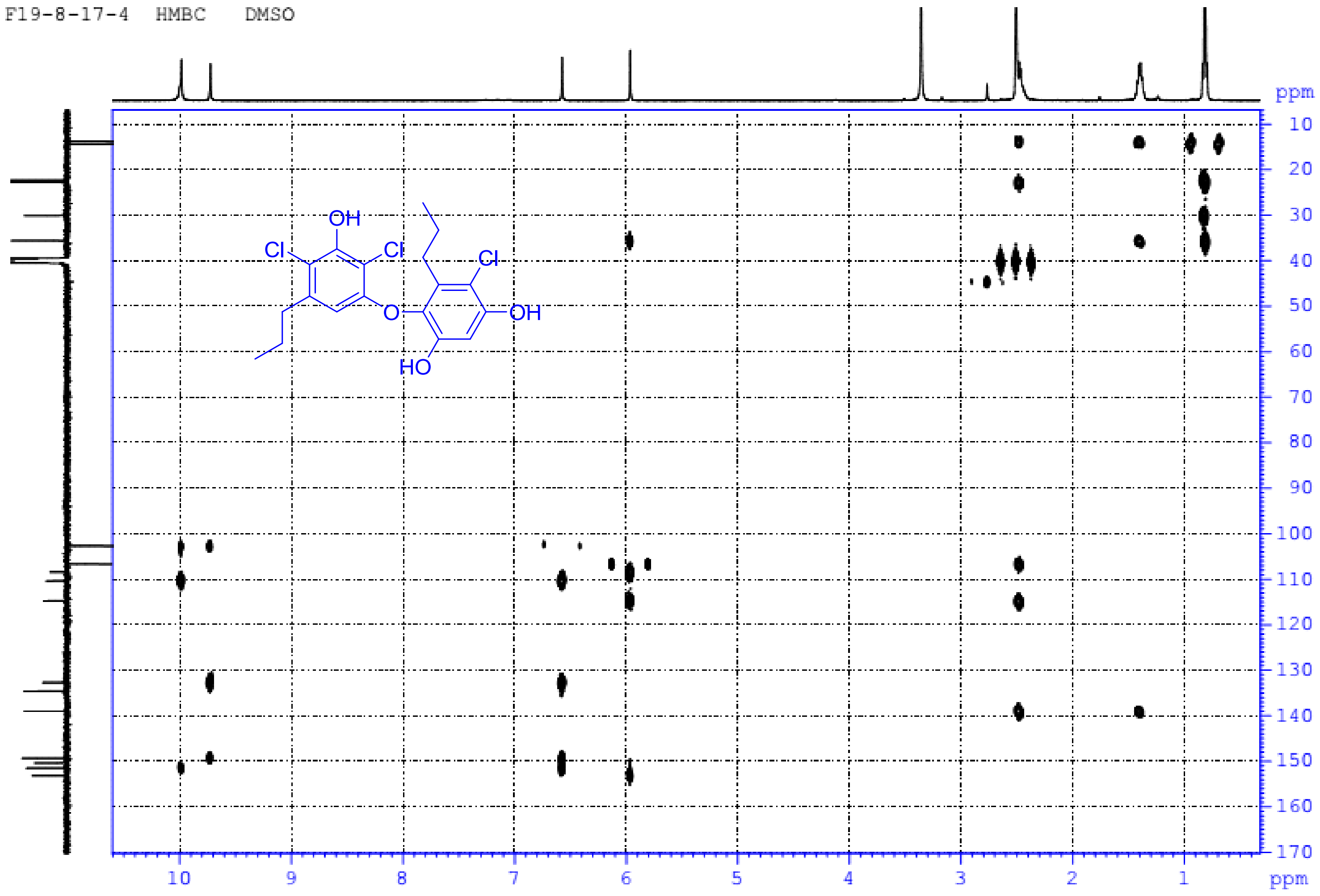

Figure S7. HMBC spectrum of compound **1** in DMSO-d6.

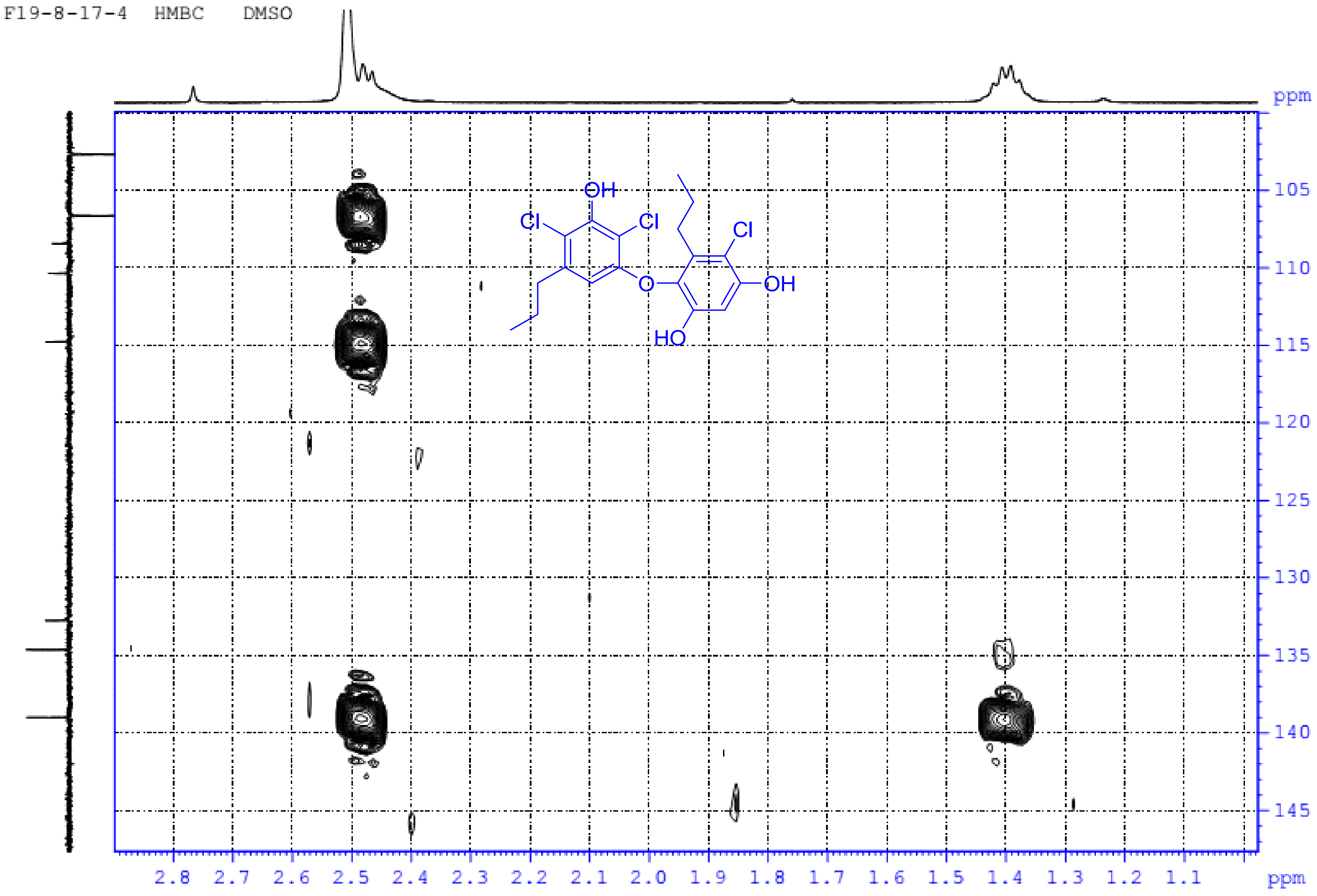

Figure S7-1. HMBC spectrum of compound **1** in DMSO-d6 (part amplification).

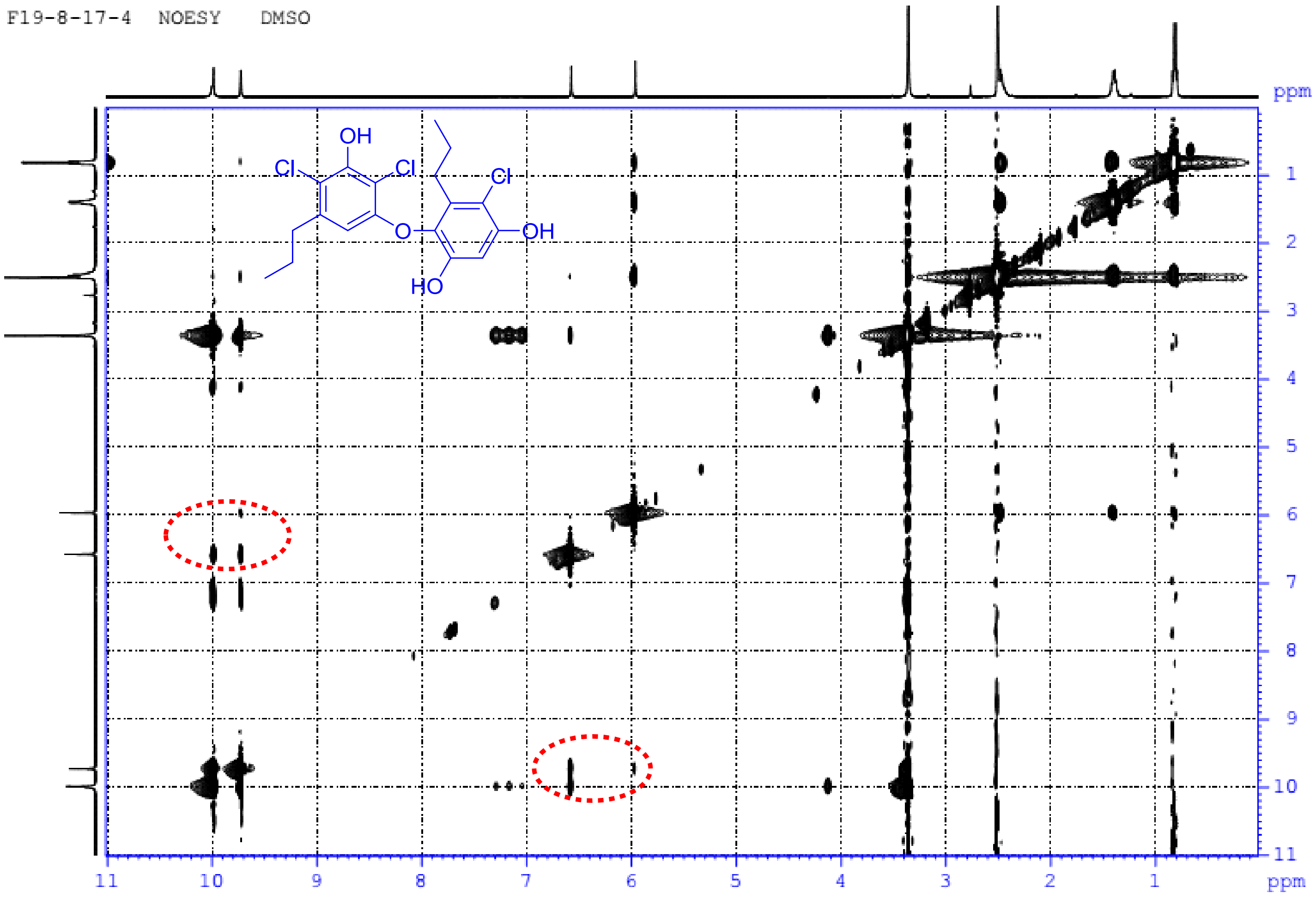

Figure S8. NOESY spectrum of compound **1** in DMSO-d6.

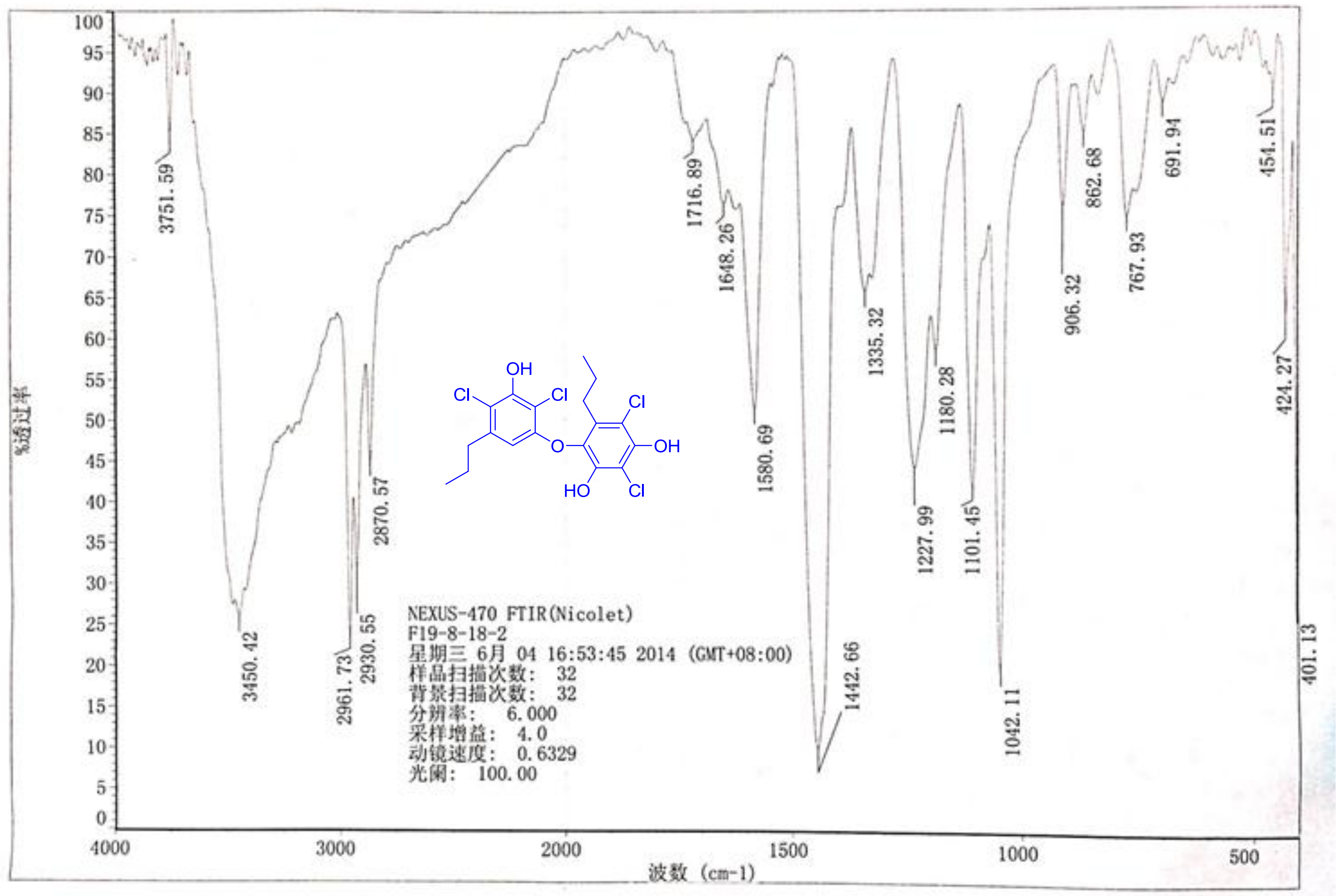

Figure S9. IR spectrum of compound **2**.

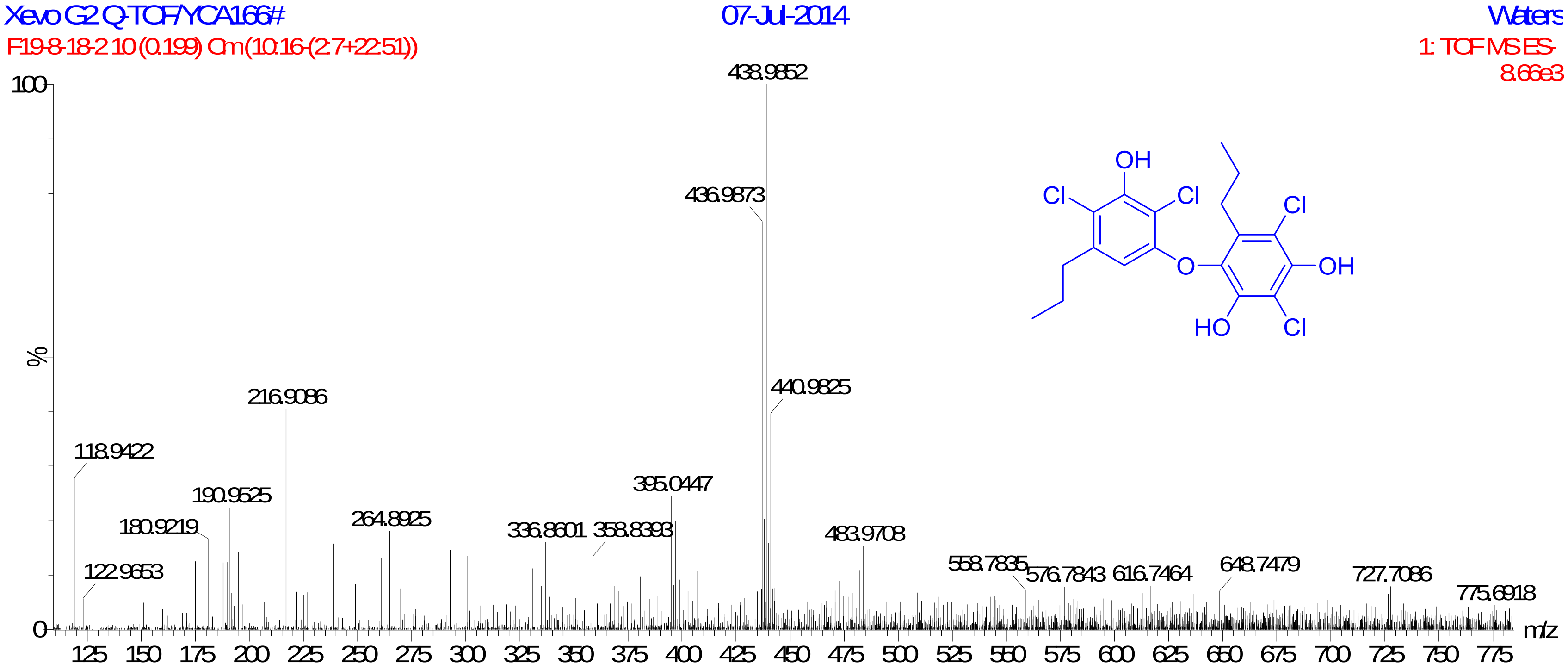

Figure S10. Negative mode HRESIMS data of compound **2**.

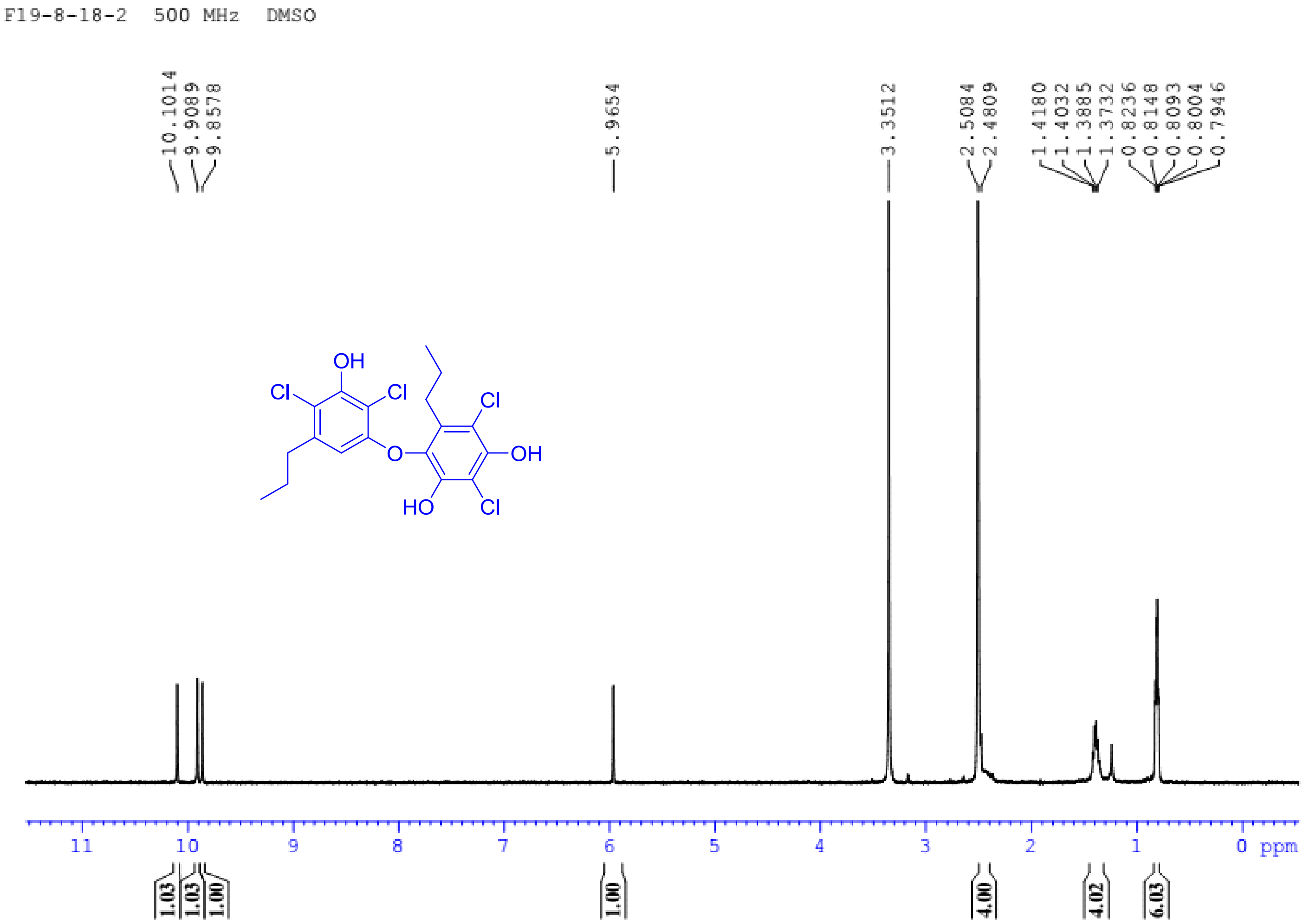

Figure S11. ^{1}H NMR spectrum of compound **2** in DMSO-d6 (500 MHz).

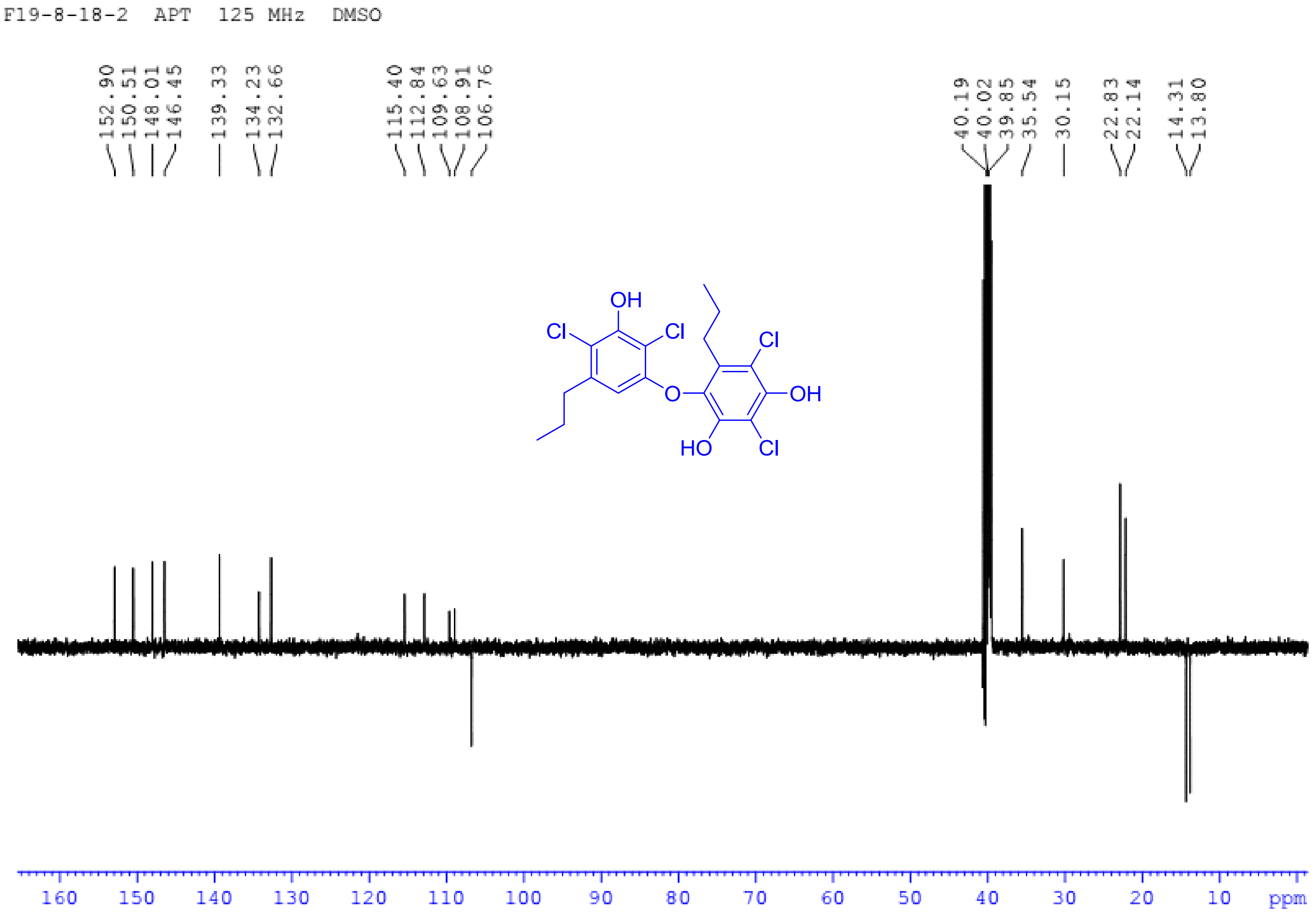

Figure S12. ^{13}C NMR spectrum of compound **2** in DMSO-d6 (125 MHz).

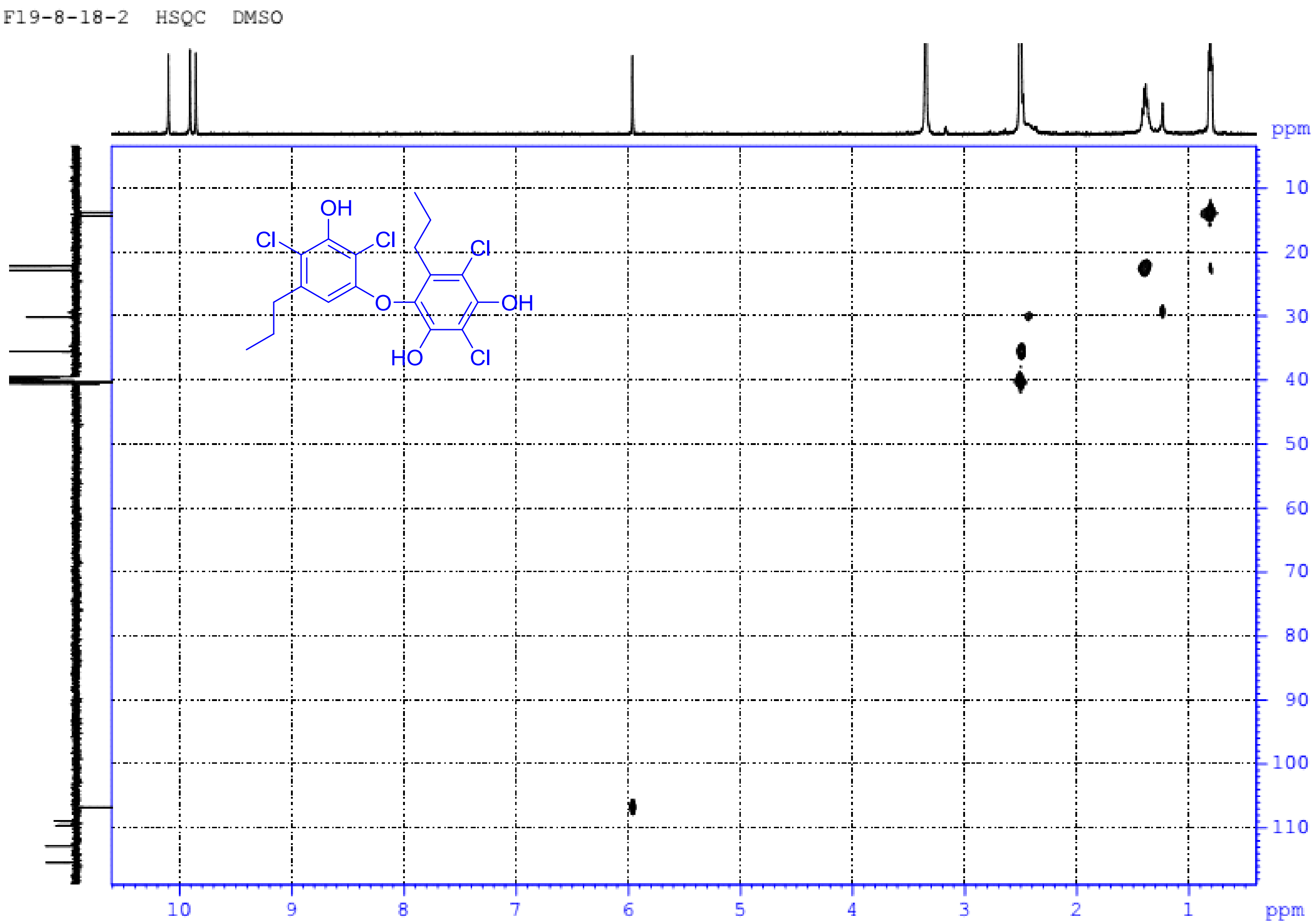

Figure S13. HSQC spectrum of compound **2** in DMSO-d6.

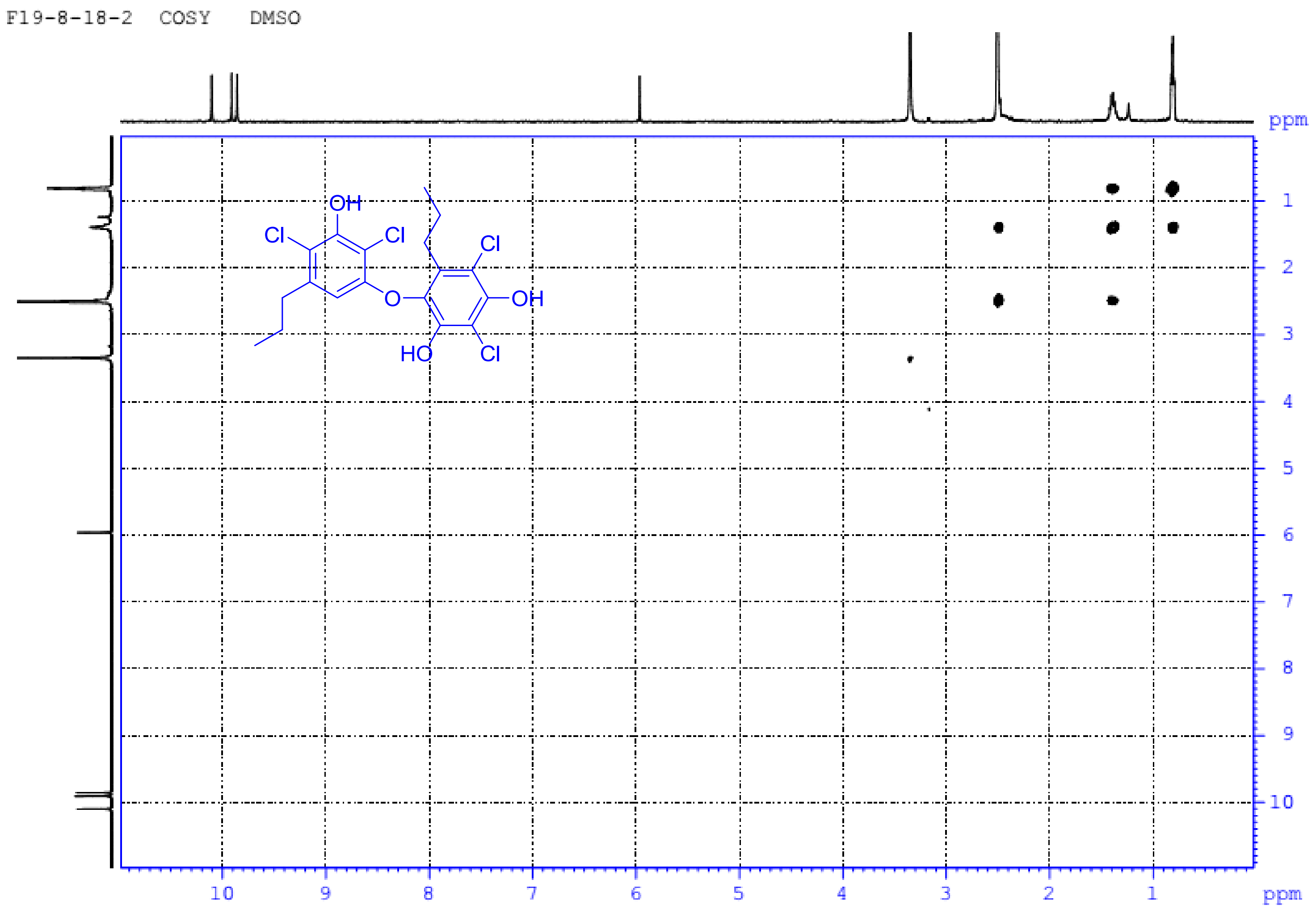

Figure S14. ^{1}H-^{1}H COSY spectrum of compound **2** in DMSO-d6.

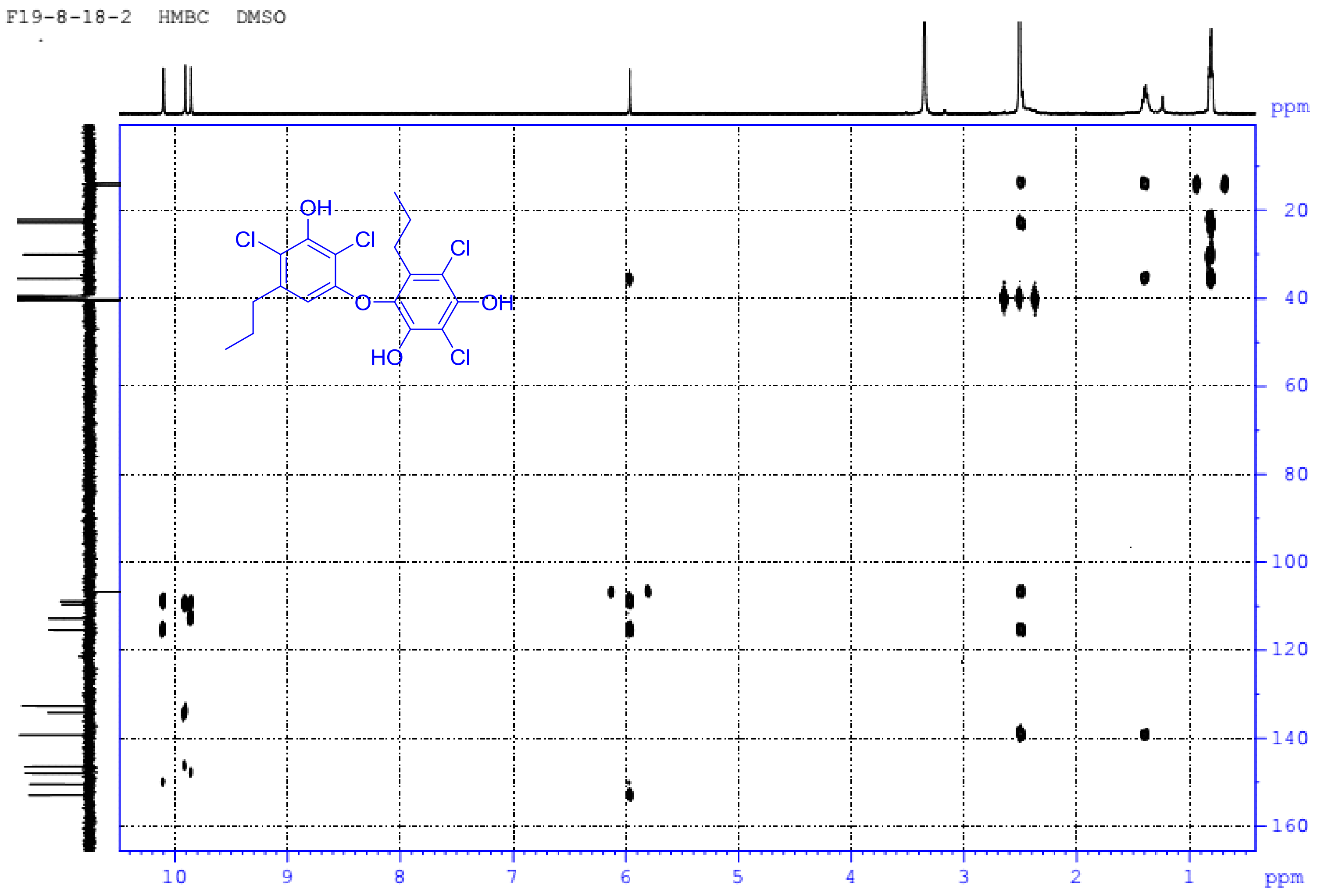

Figure S15. HMBC spectrum of compound **2** in DMSO-d6.

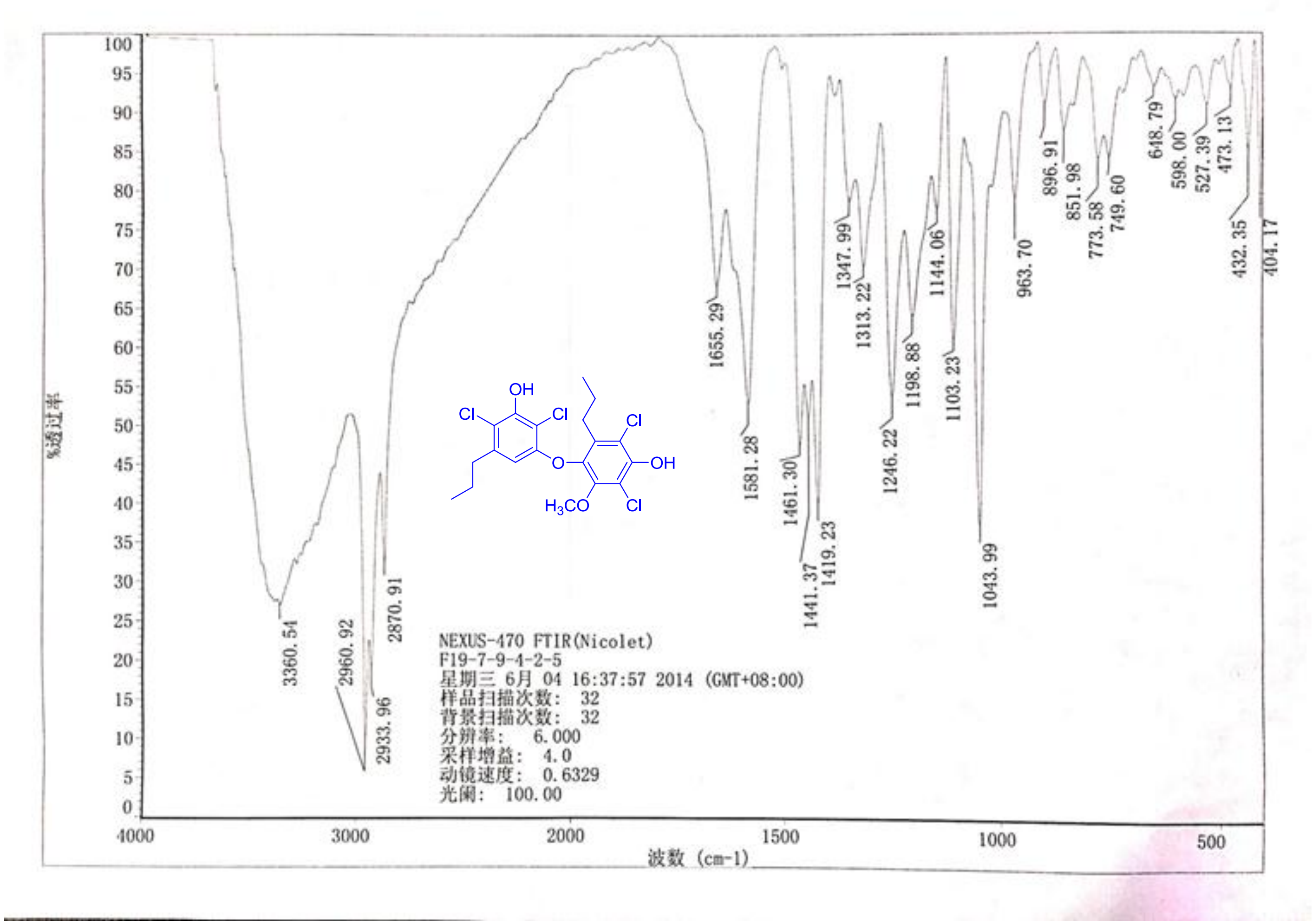

Figure S16. IR spectrum of compound **3**.

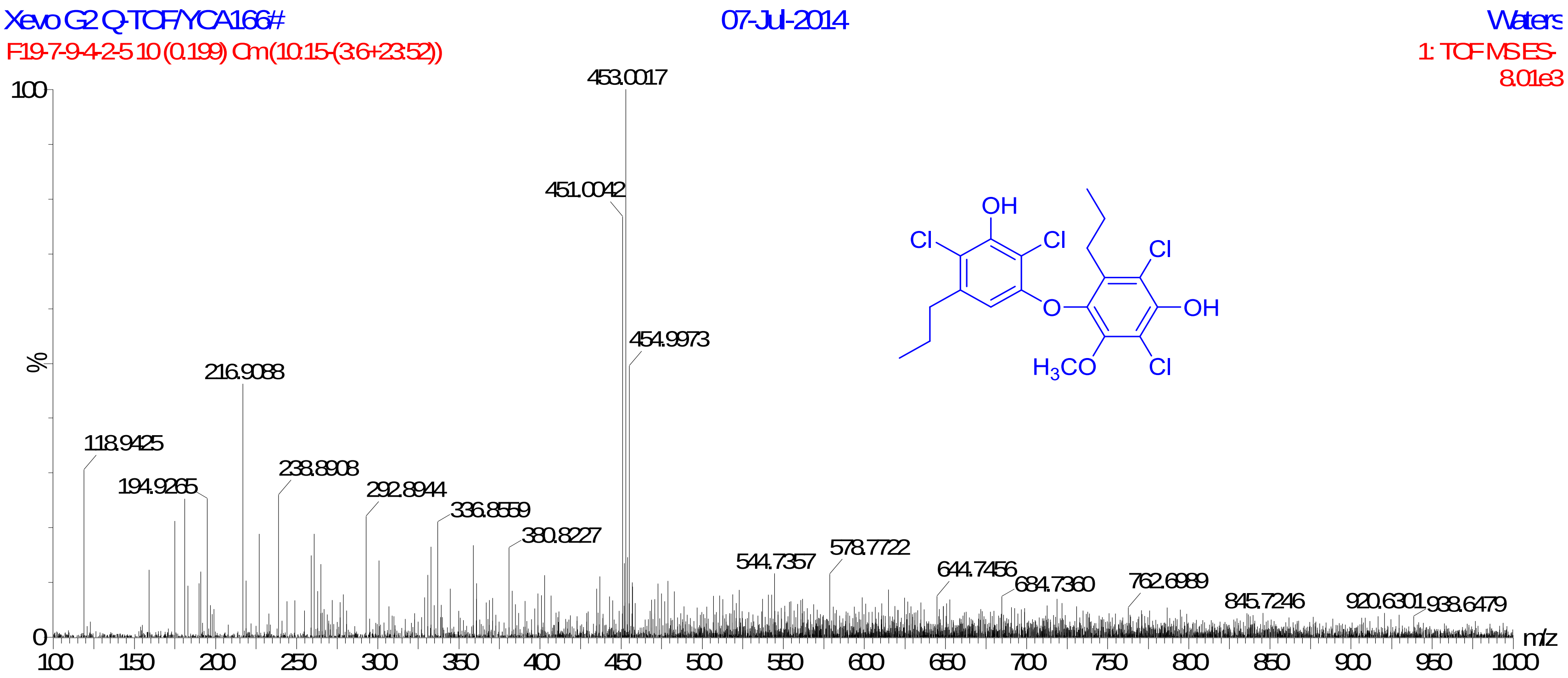

Figure S17. Negative mode HRESIMS data of compound **3**.

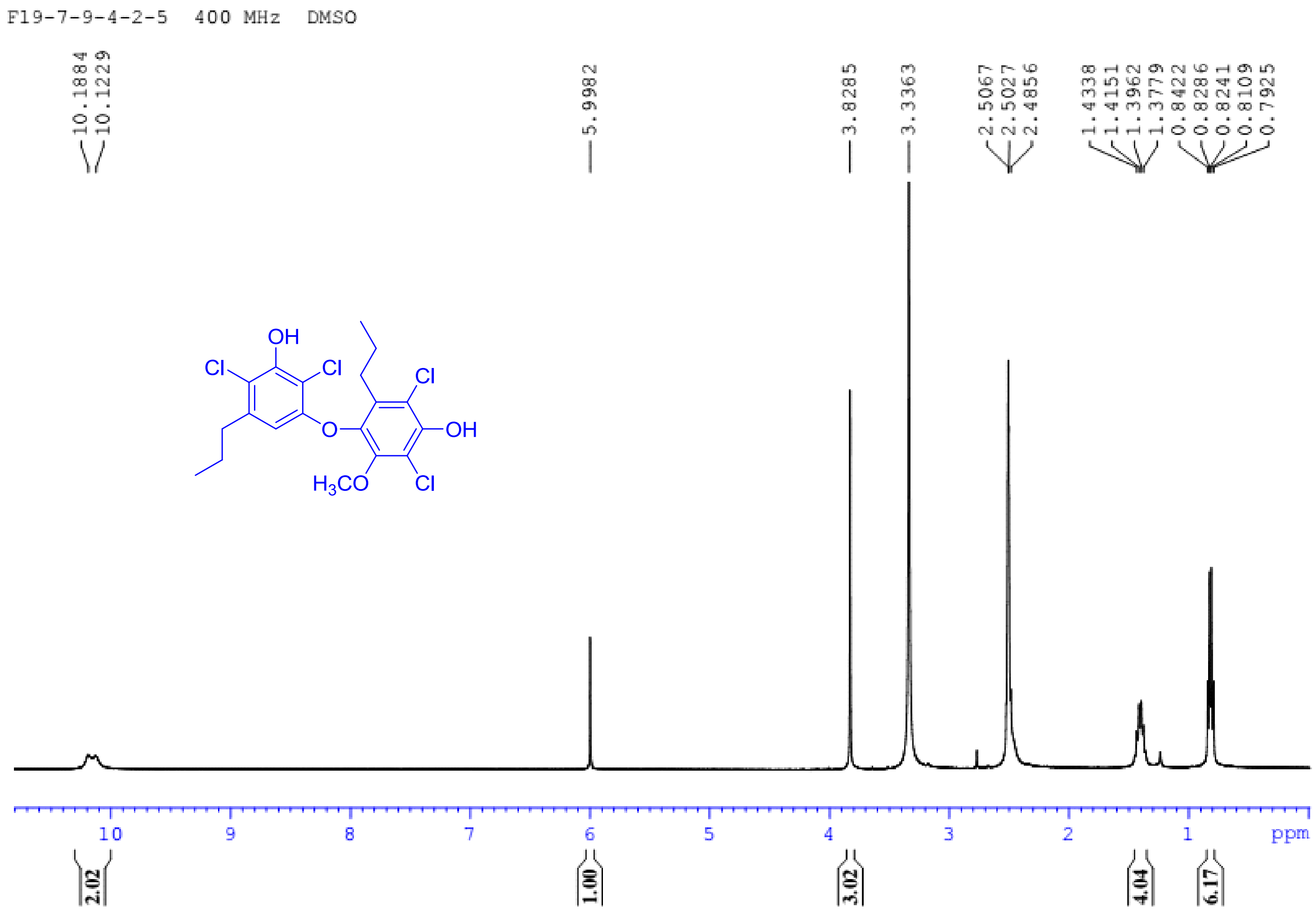

Figure S18. ^{1}H NMR spectrum of compound **3** in DMSO-d6 (400 MHz).

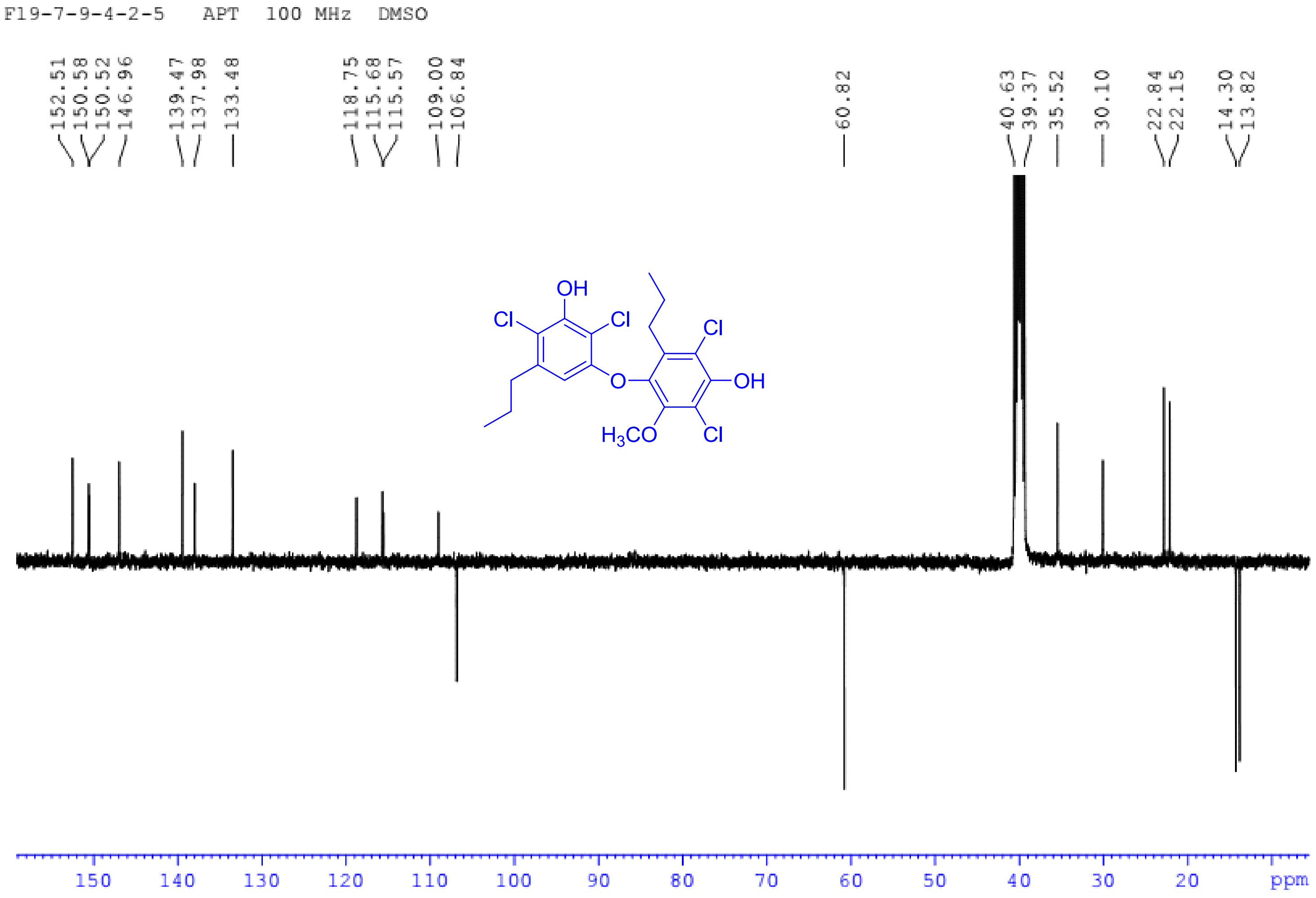

Figure S19. ^{13}C NMR spectrum of compound **3** in DMSO-d6 (100 MHz).

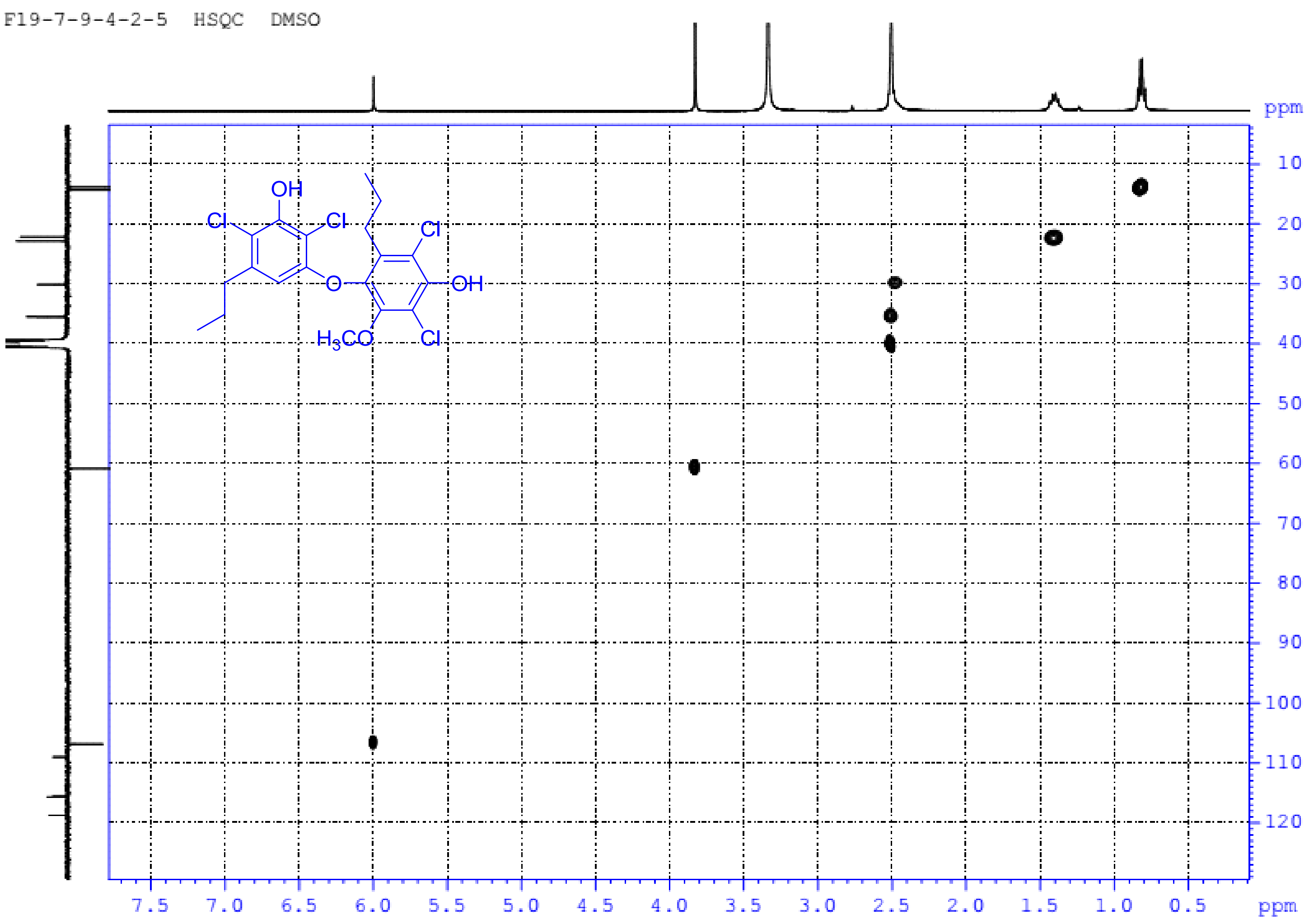

Figure S20. HSQC spectrum of compound **3** in DMSO-d6.

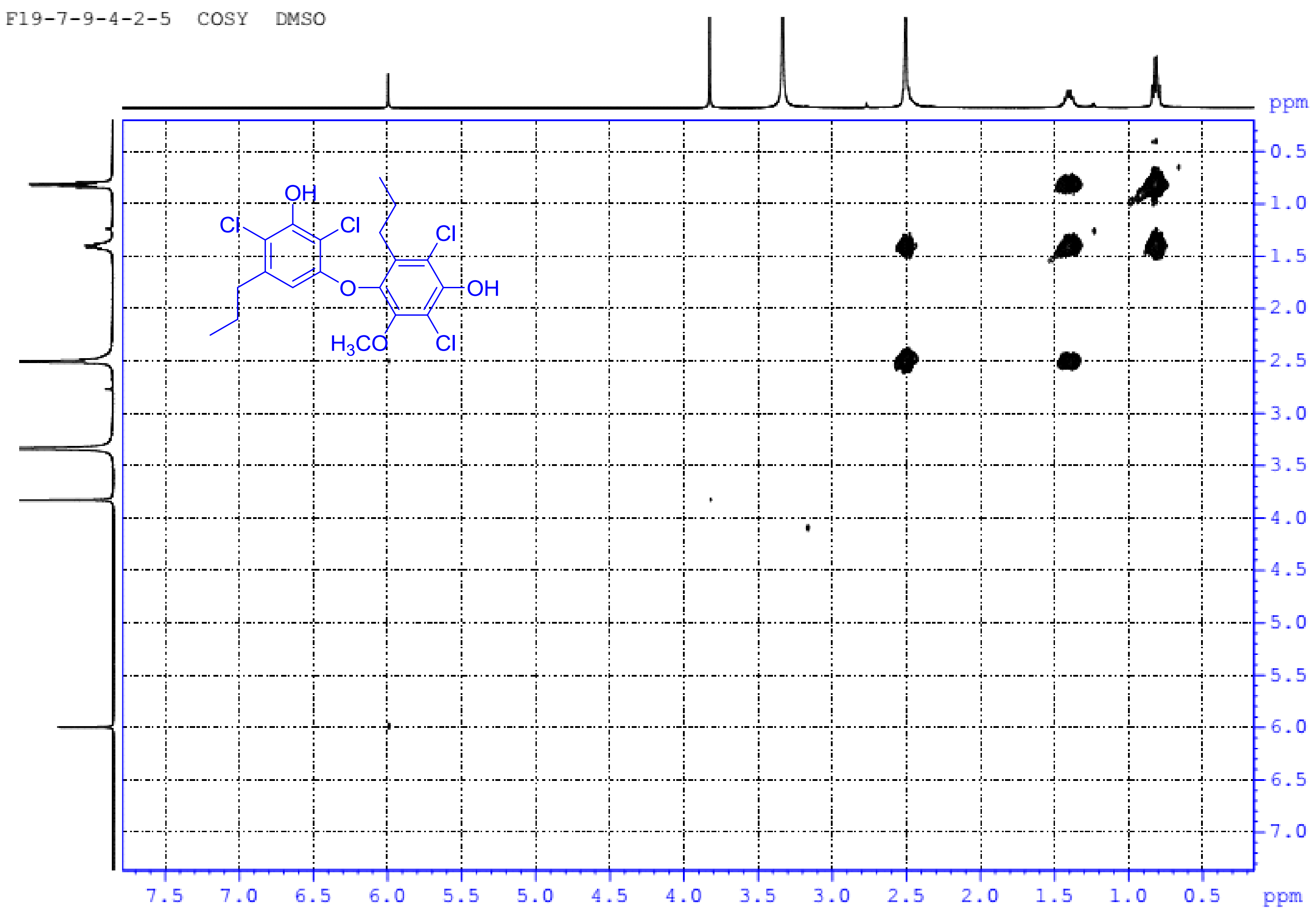

Figure S21. ^{1}H-^{1}H COSY spectrum of compound **3** in DMSO-d6.

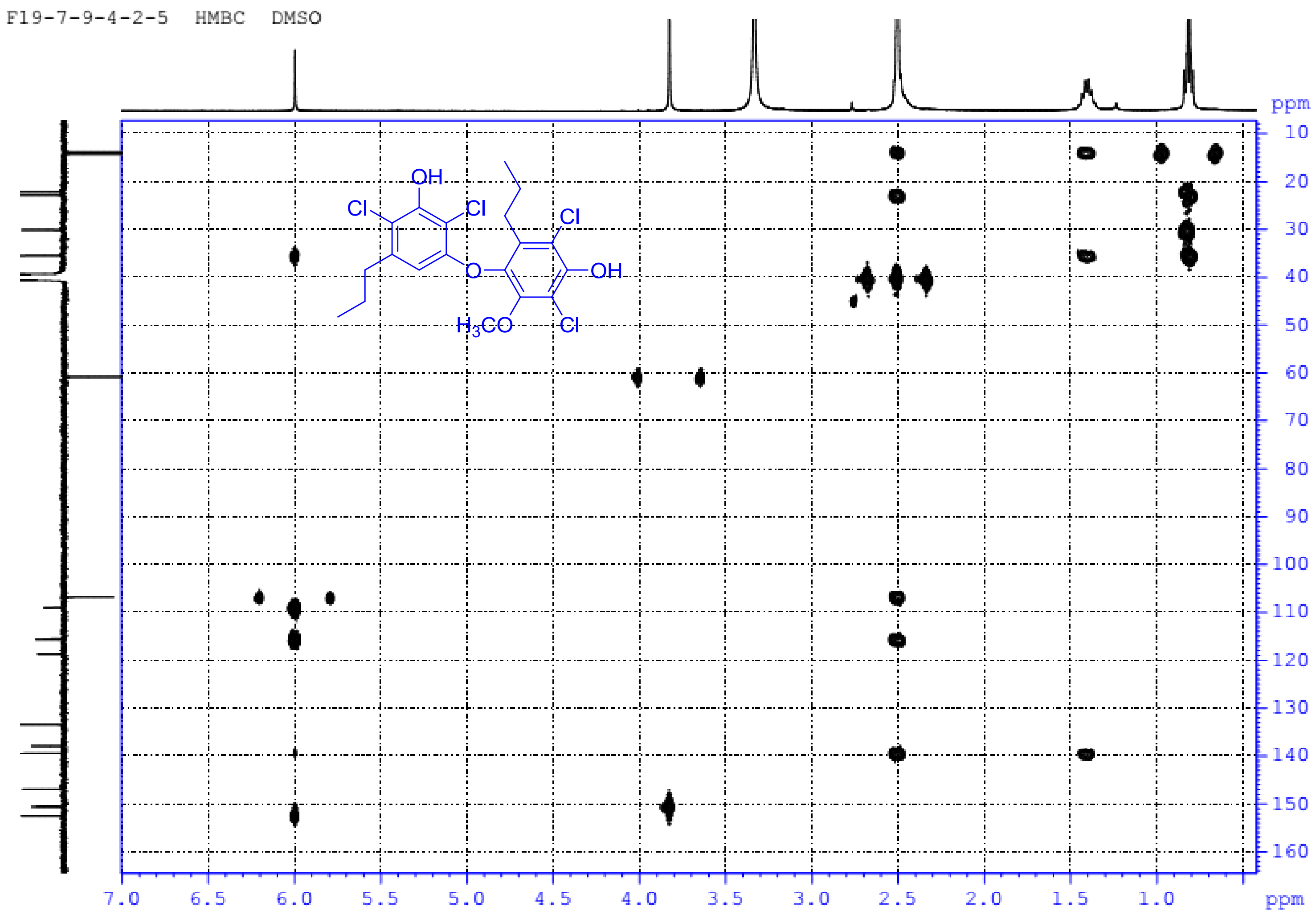

Figure S22. HMBC spectrum of compound **3** in DMSO-d6.

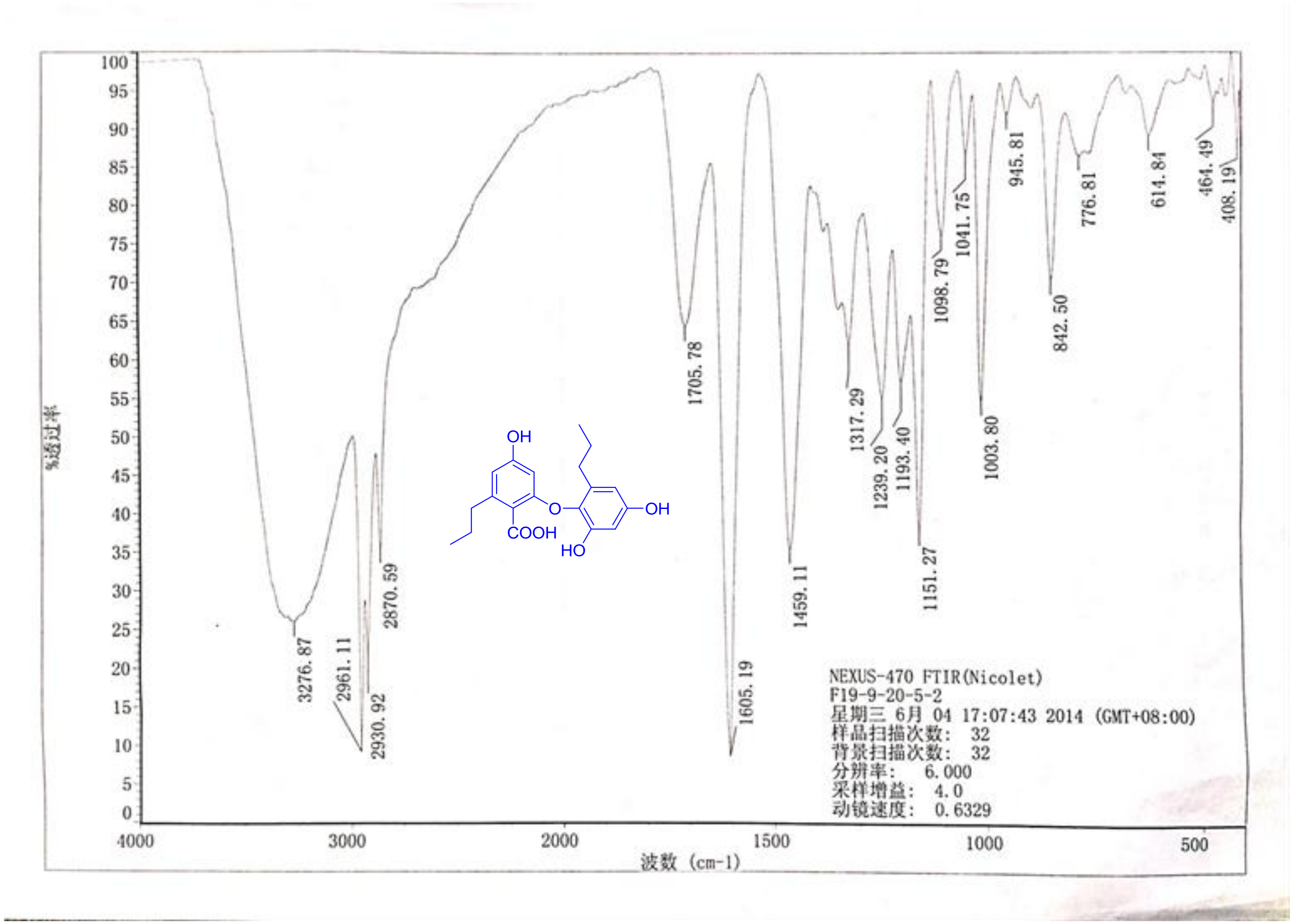

Figure S23. IR spectrum of compound **4**.

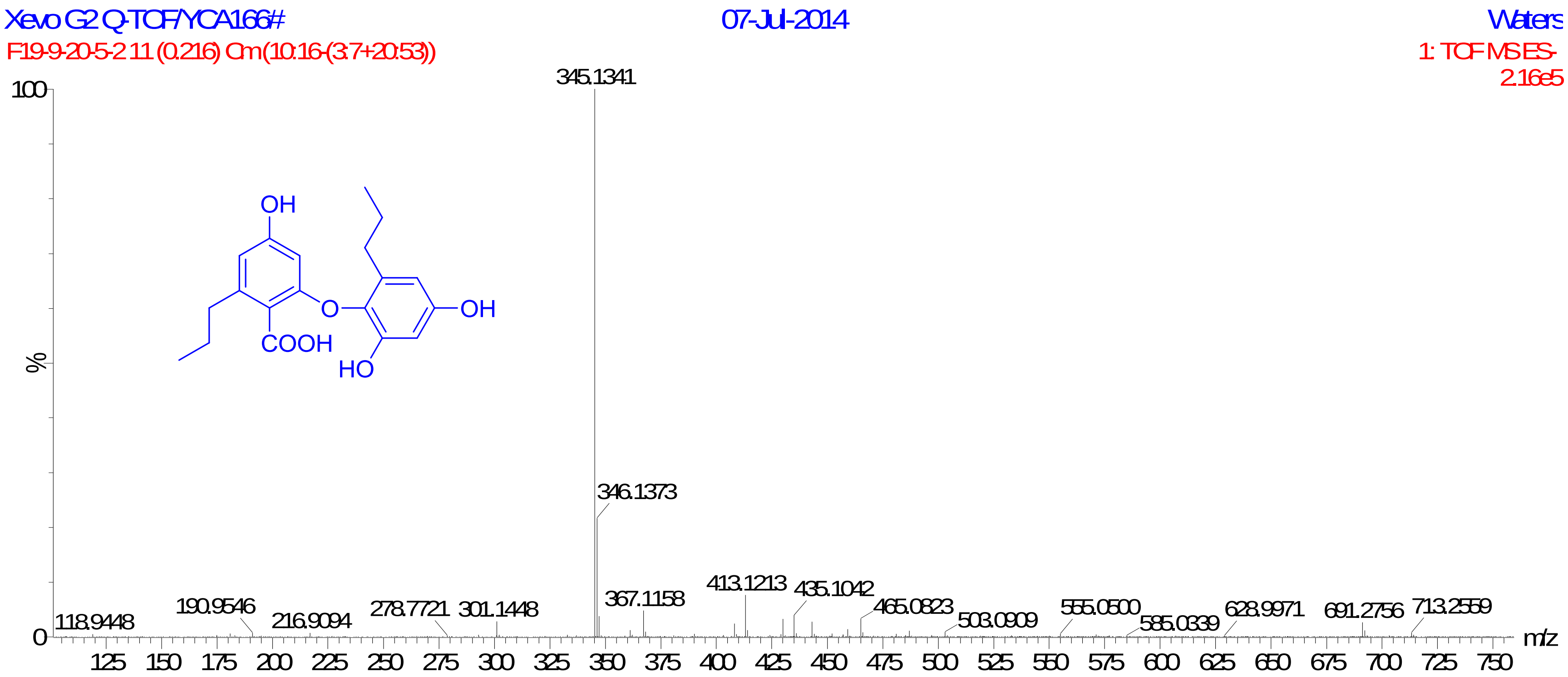

Figure S24. Negative mode HRESIMS data of compound **4**.

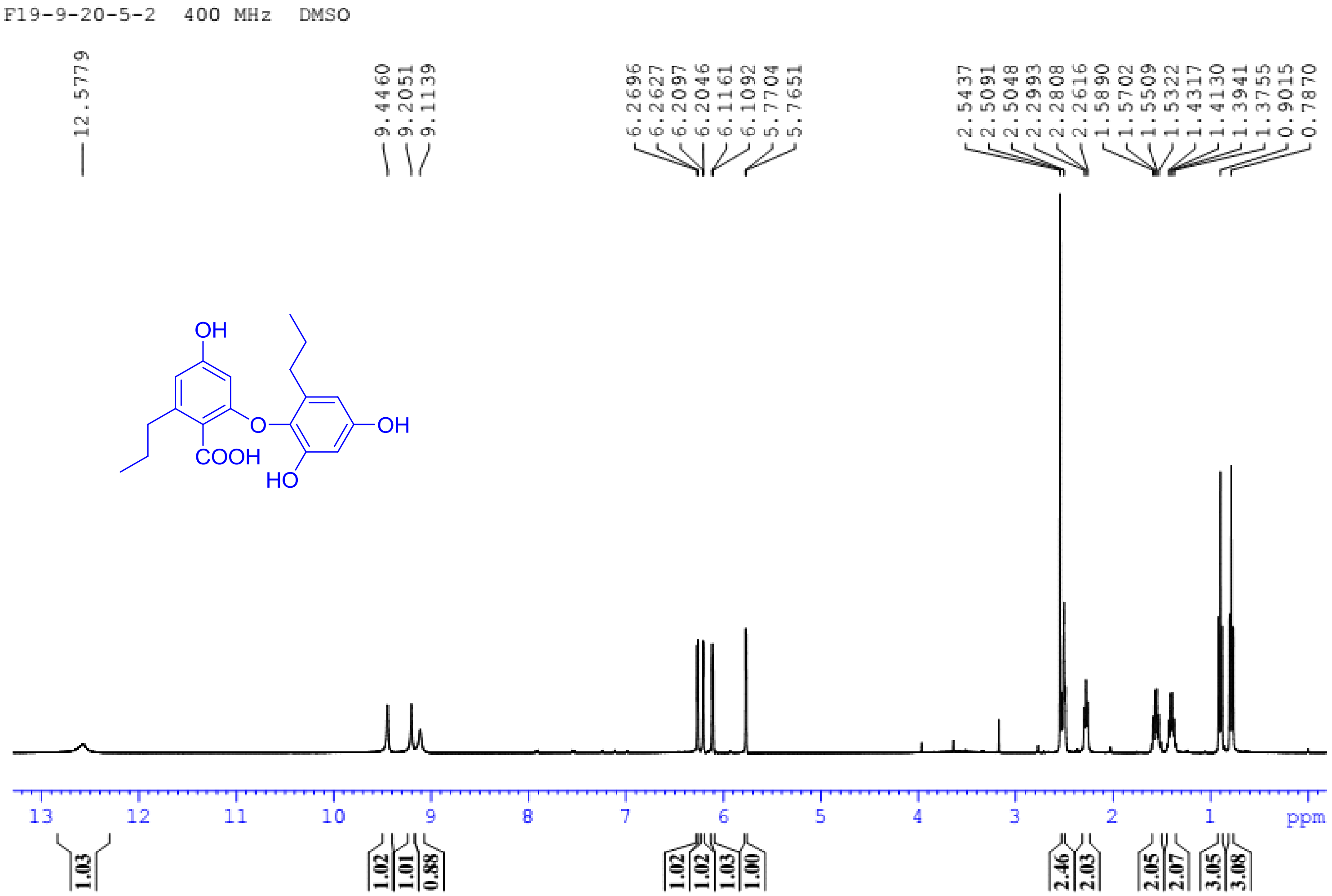

Figure S25. ^{1}H NMR spectrum of compound **4** in DMSO-d6 (400 MHz).

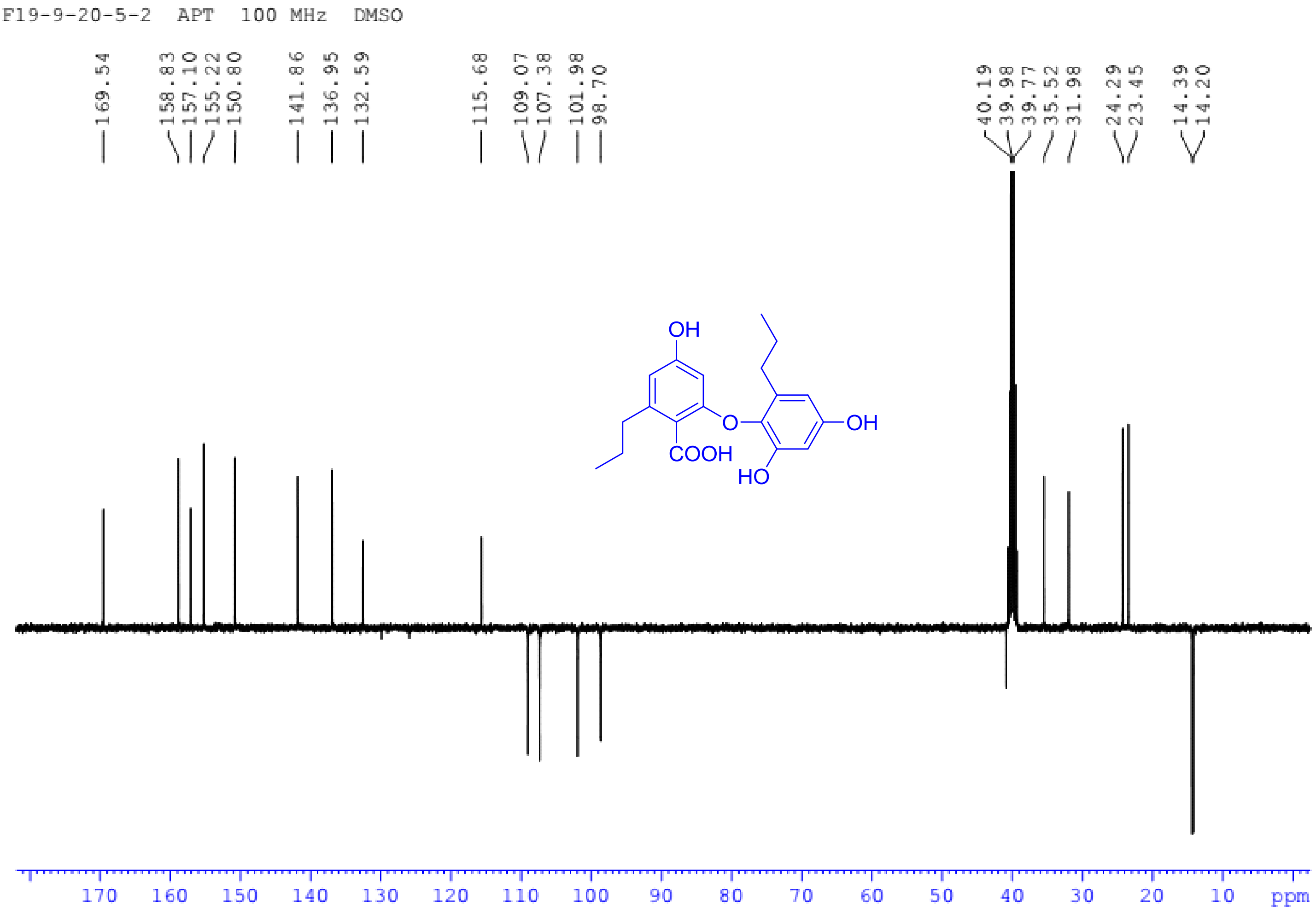

Figure S26. ^{13}C NMR spectrum of compound **4** in DMSO-d6 (100 MHz).

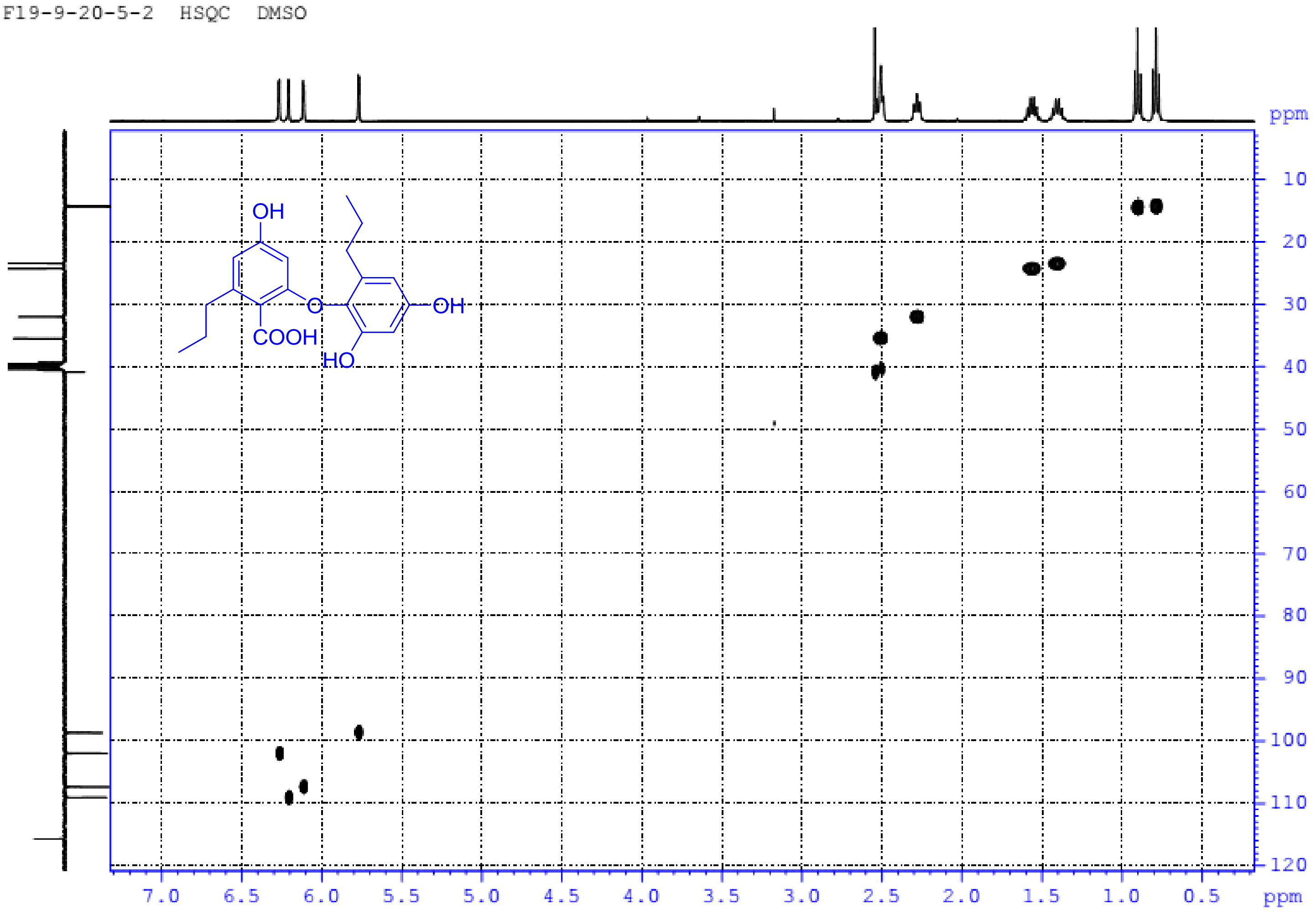

Figure S27. HSQC spectrum of compound **4** in DMSO-d6.

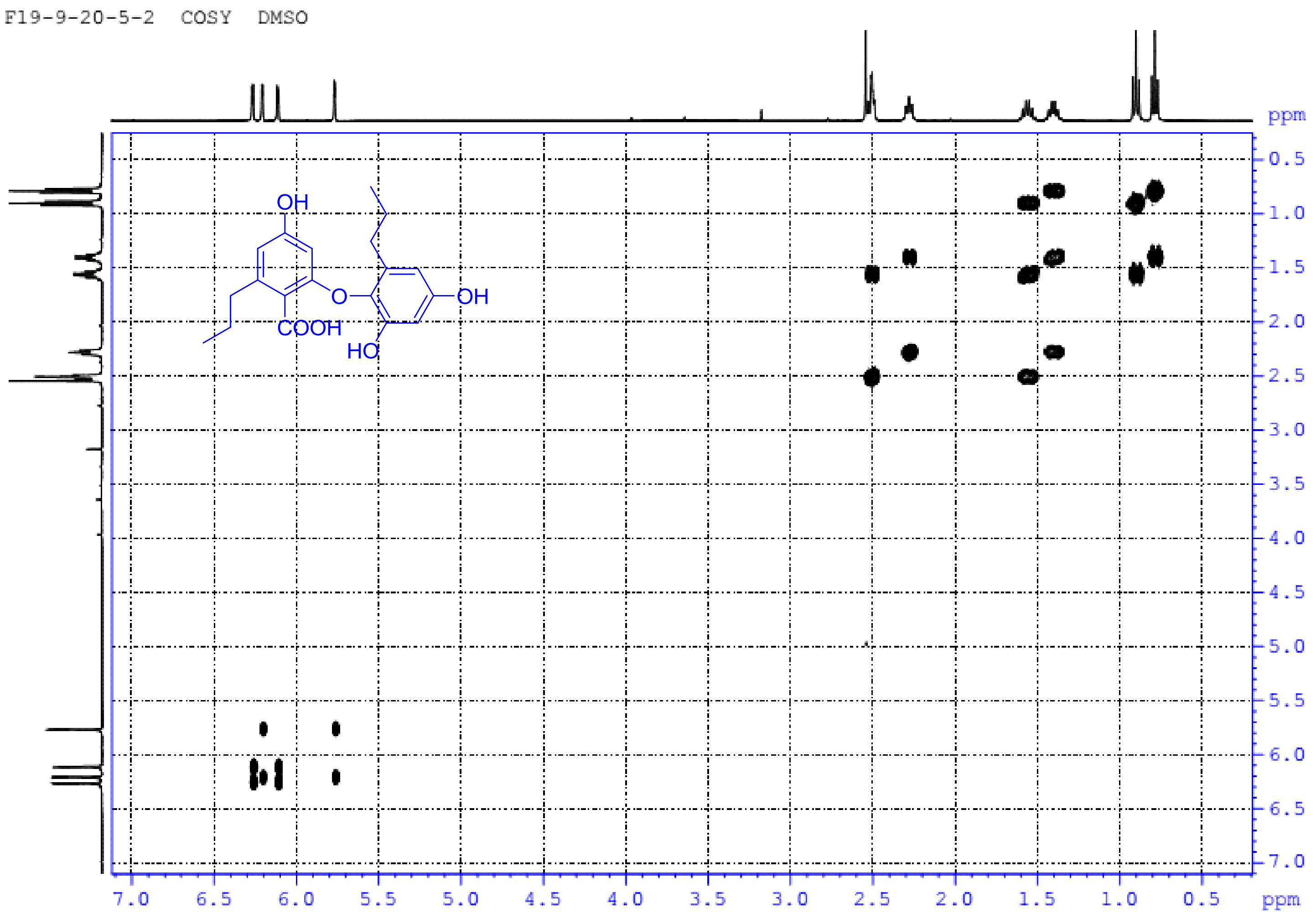

Figure S28. ^{1}H-^{1}H COSY spectrum of compound **4** in DMSO-d6.

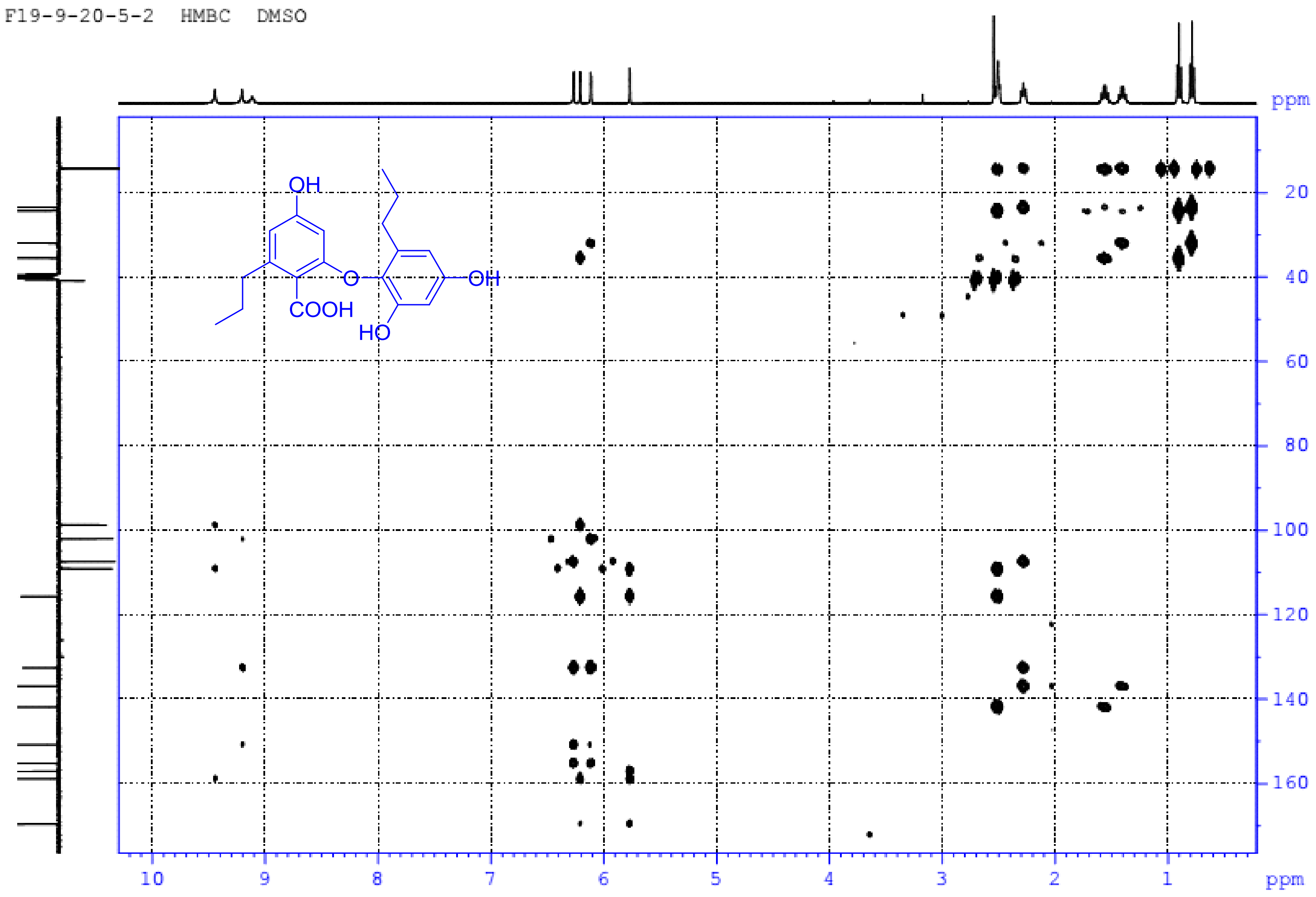

Figure S29. HMBC spectrum of compound **4** in DMSO-d6.

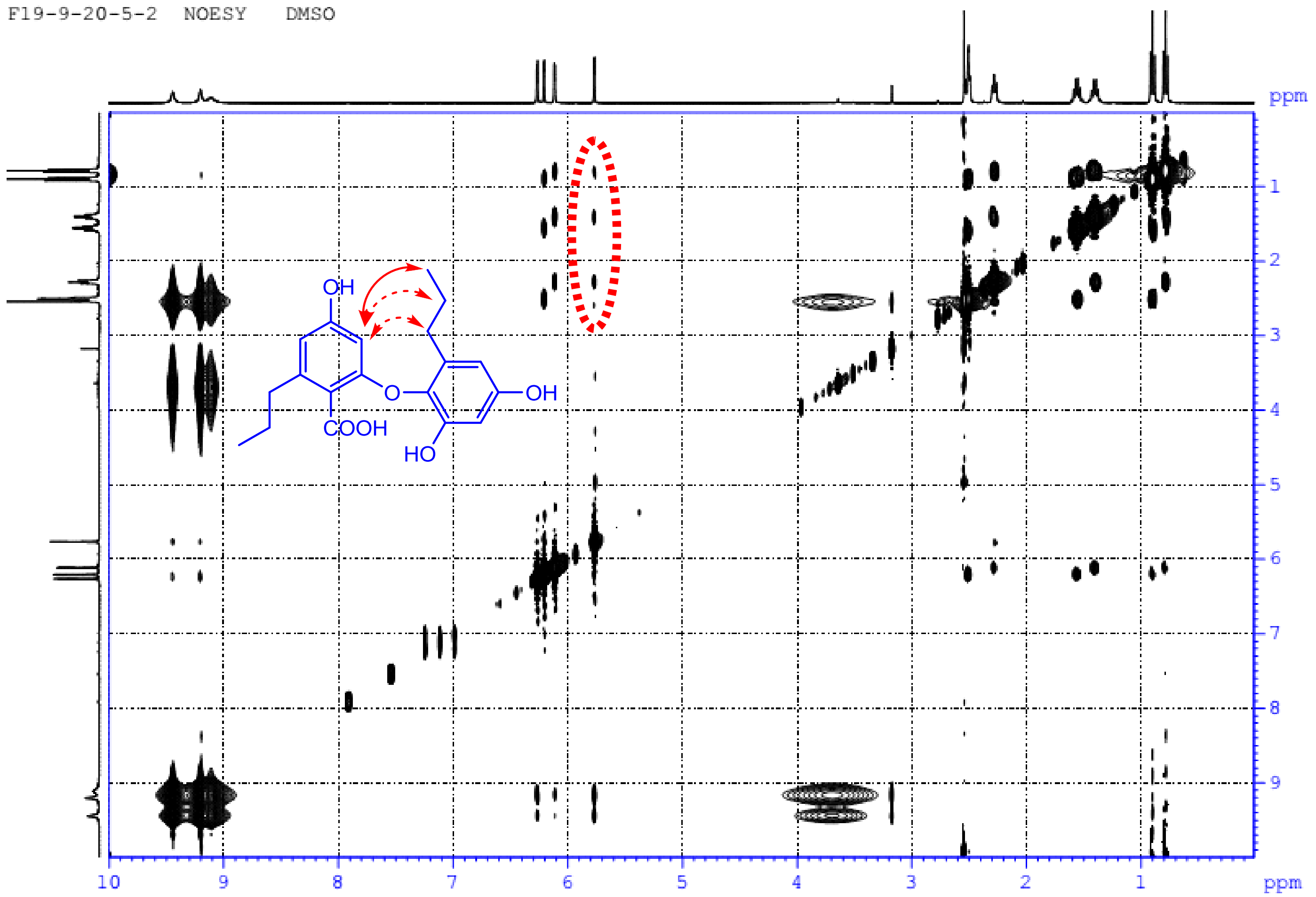

Figure S30. NOESY spectrum of compound **4** in DMSO-d6.

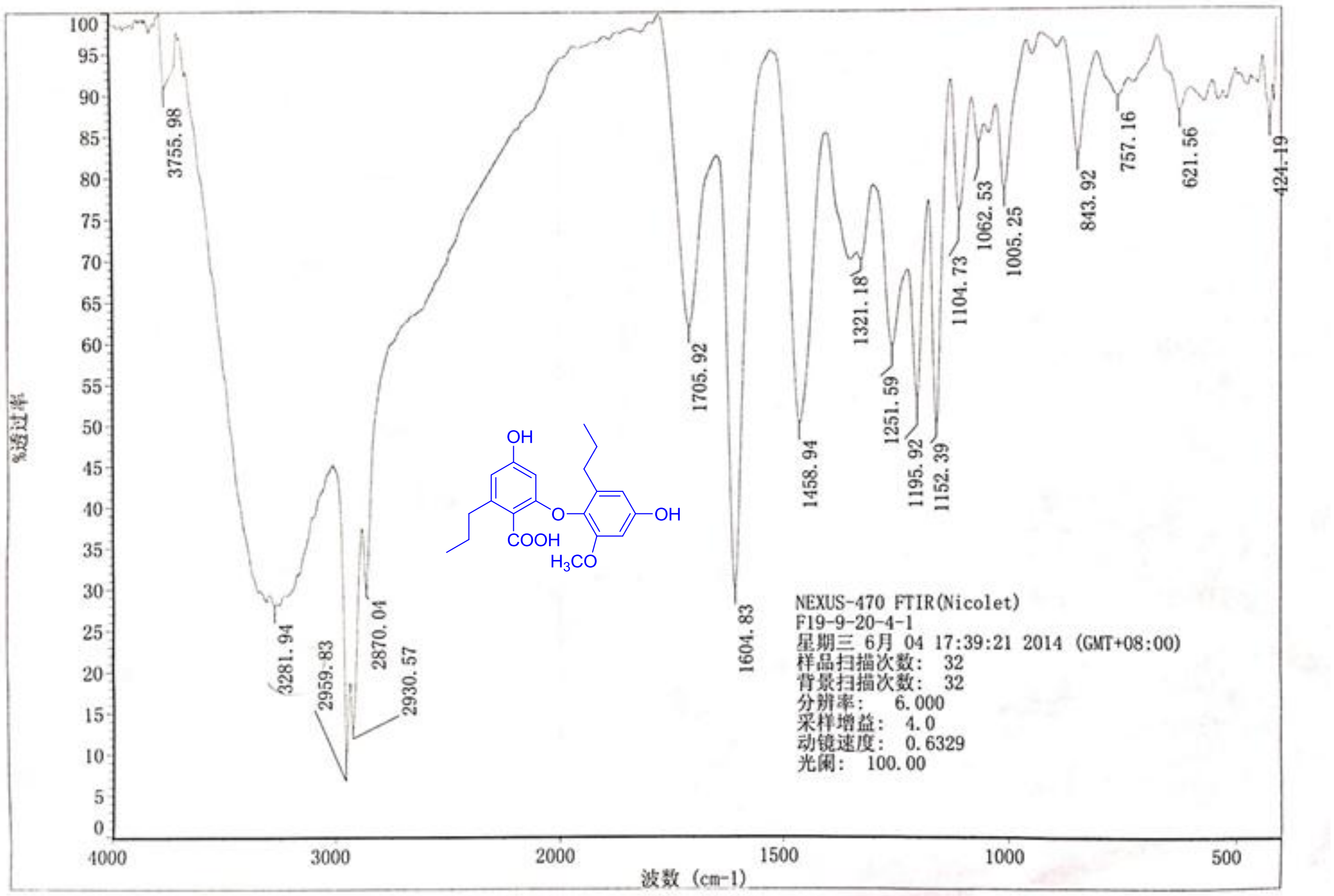

Figure S31. IR spectrum of compound **5**.

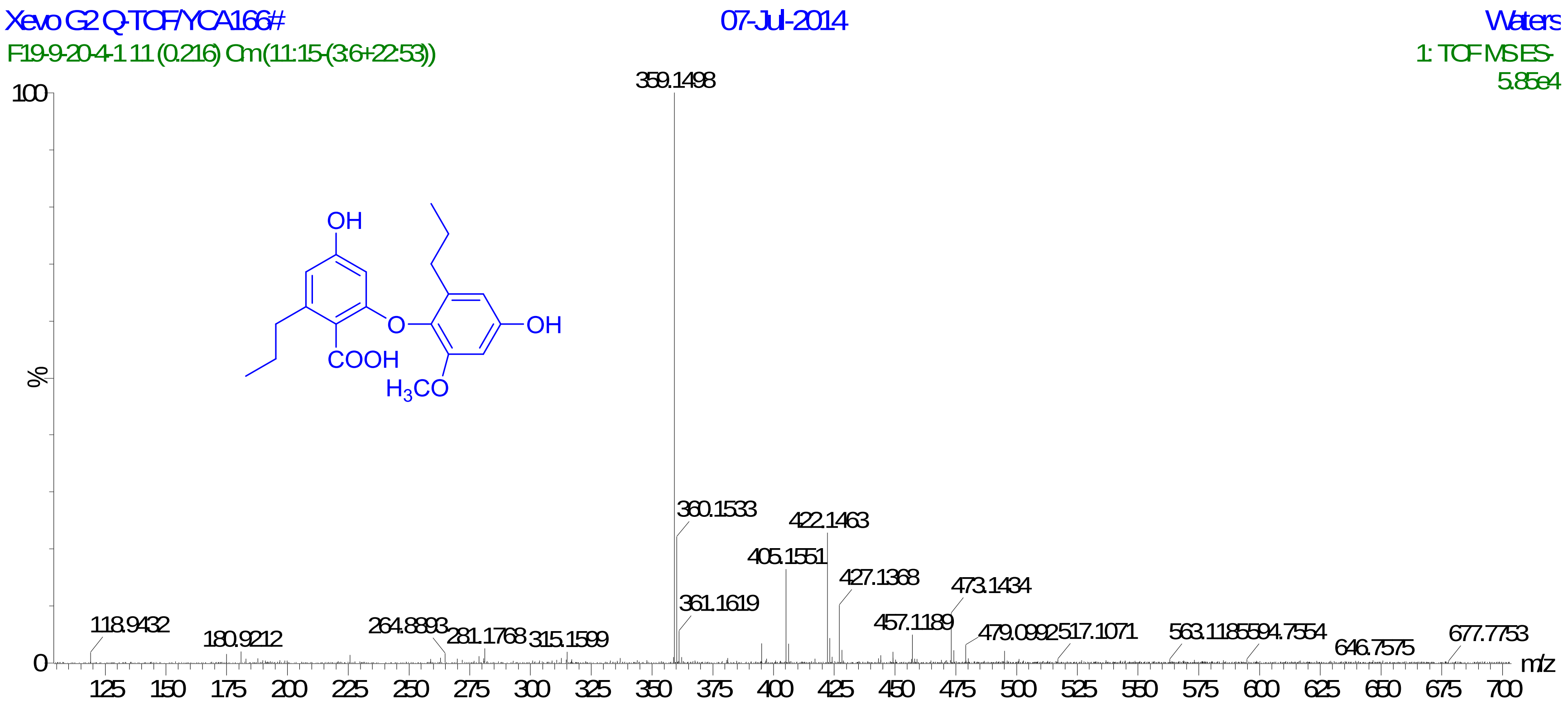

Figure S32. Negative mode HRESIMS data of compound **5**.

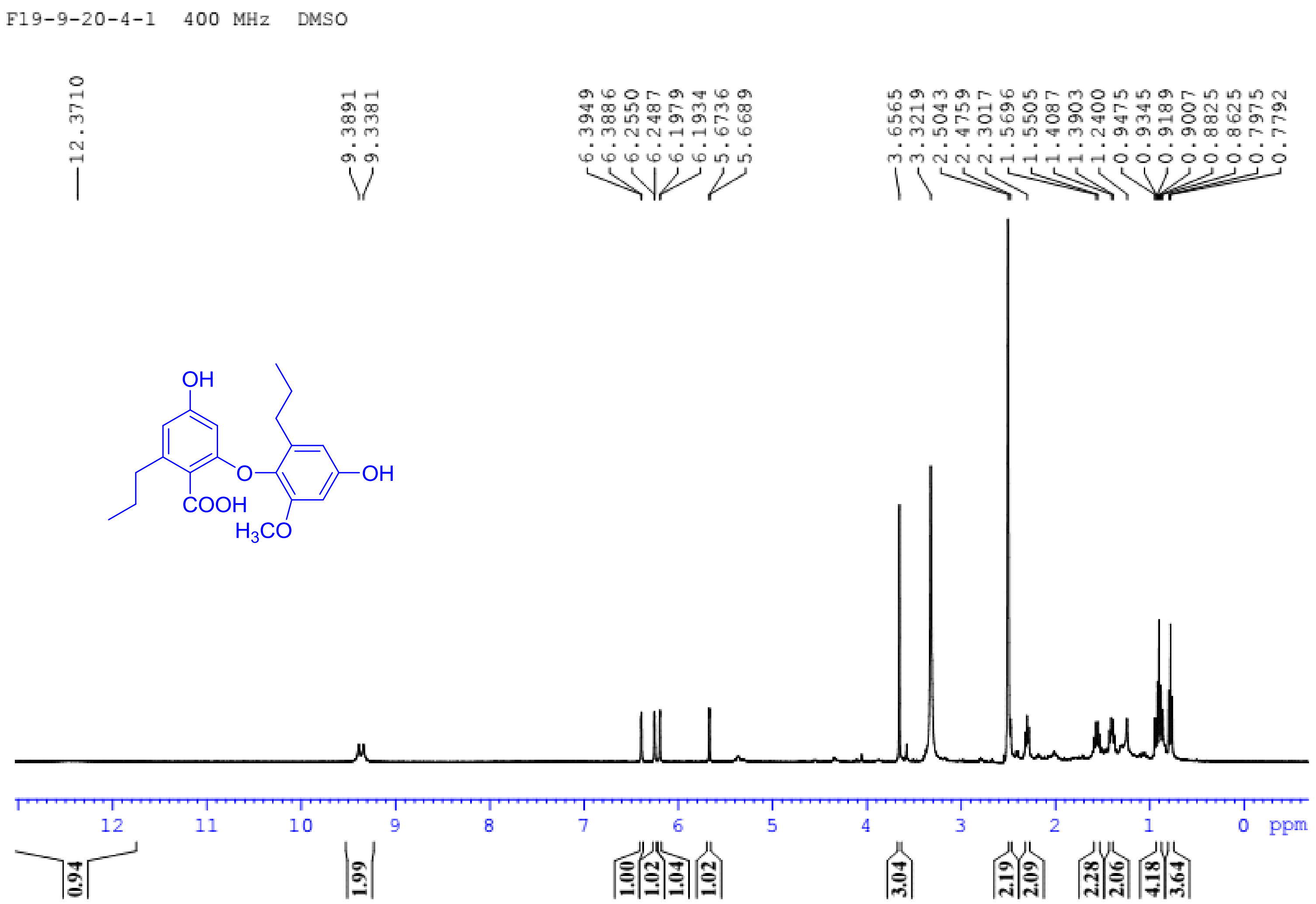

Figure S33. ^{1}H NMR spectrum of compound **5** in DMSO-d6 (400 MHz).

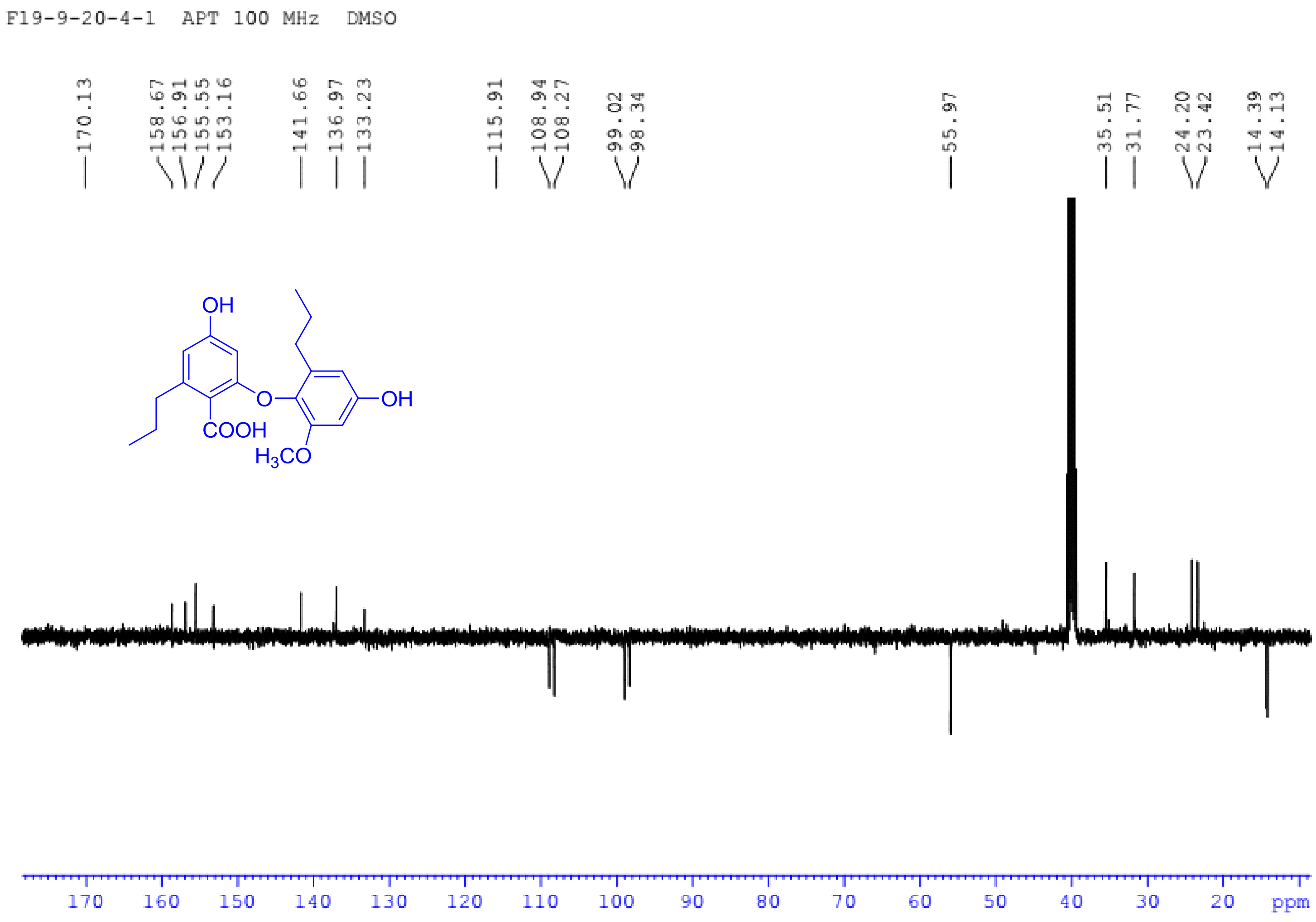

Figure S34. ^{13}C NMR spectrum of compound **5** in DMSO-d6 (100 MHz).

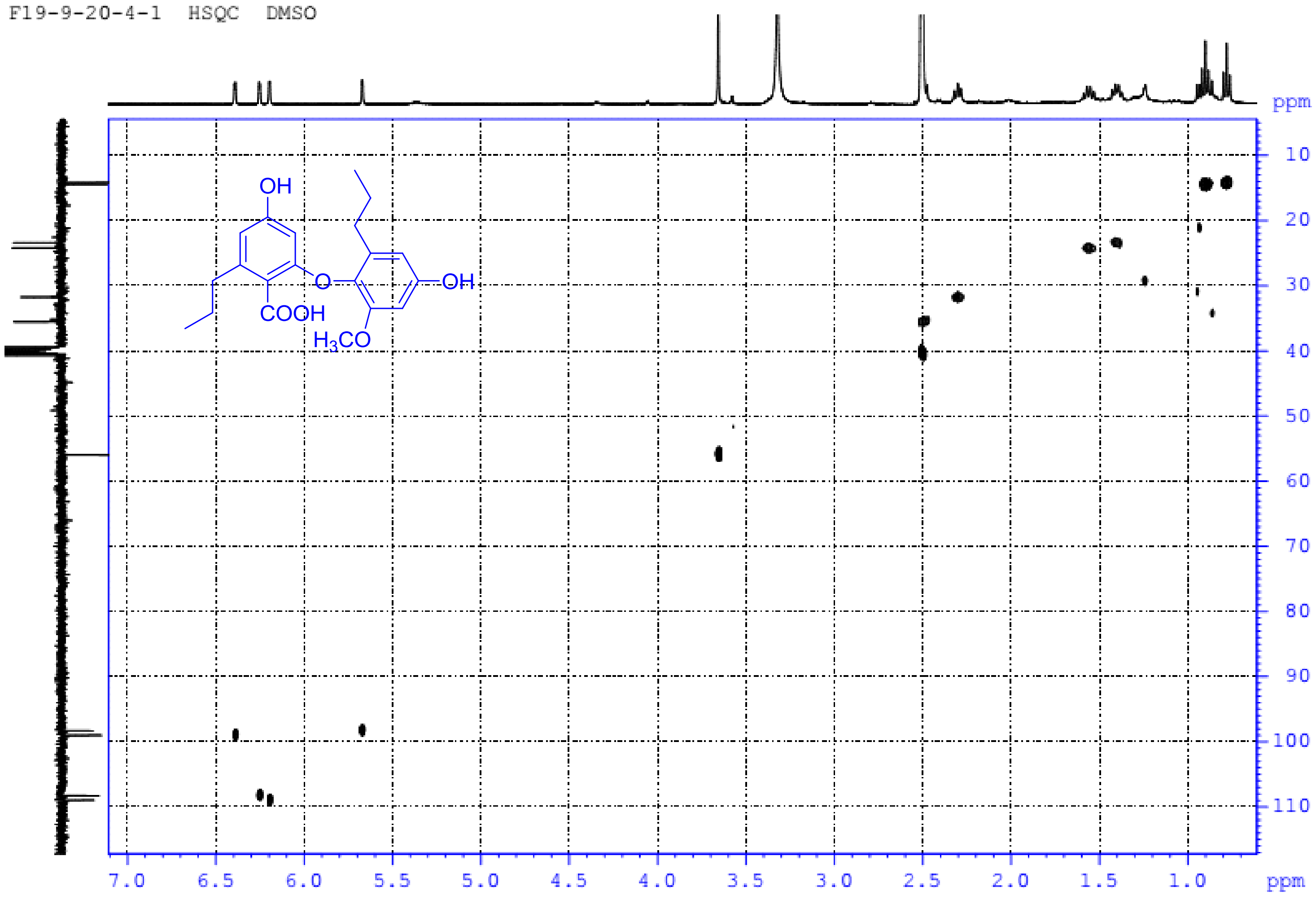

Figure S35. HSQC spectrum of compound **5** in DMSO-d6.

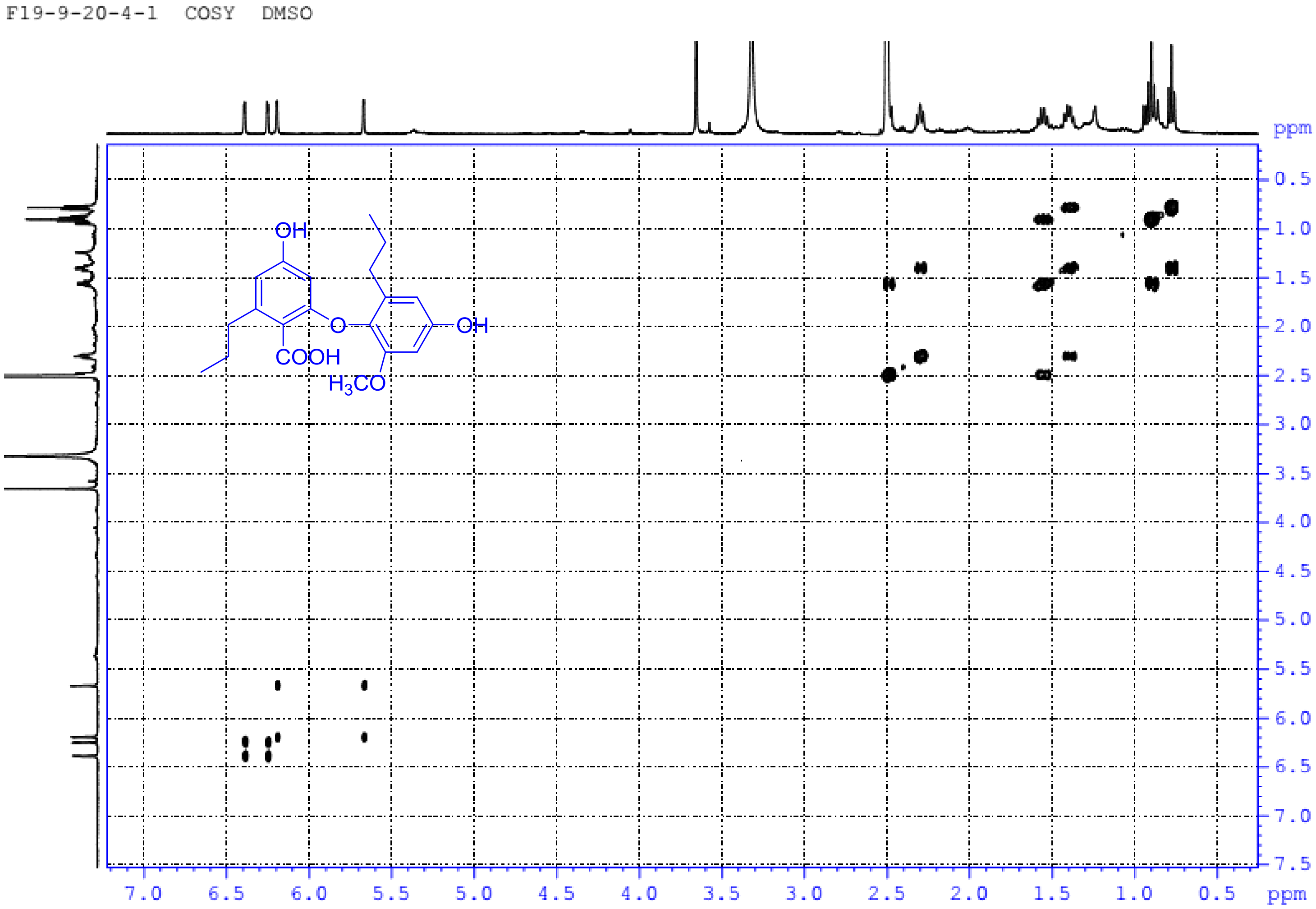

Figure S36. ^{1}H-^{1}H COSY spectrum of compound **5** in DMSO-d6.

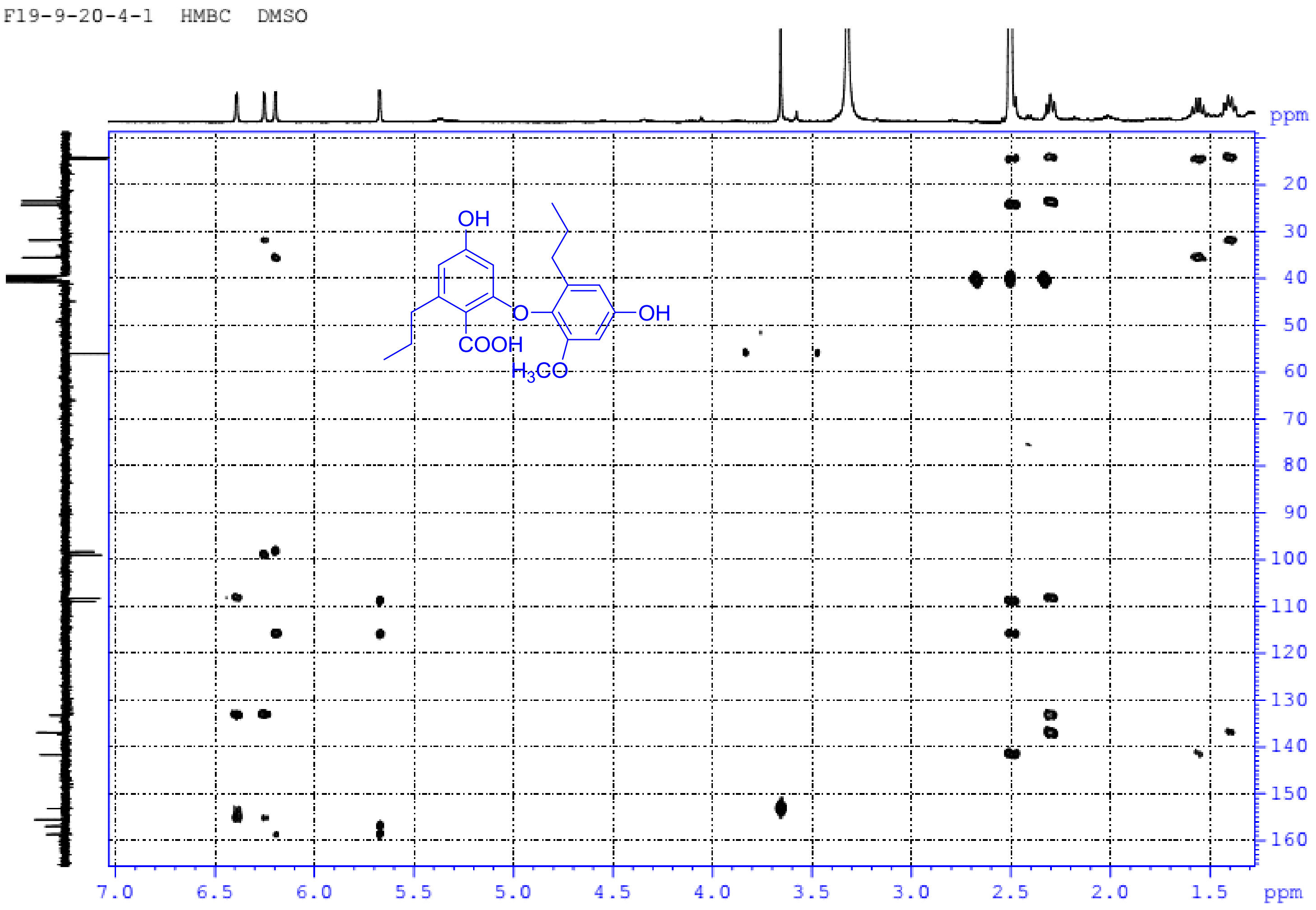

Figure S37. HMBC spectrum of compound **5** in DMSO-d6.

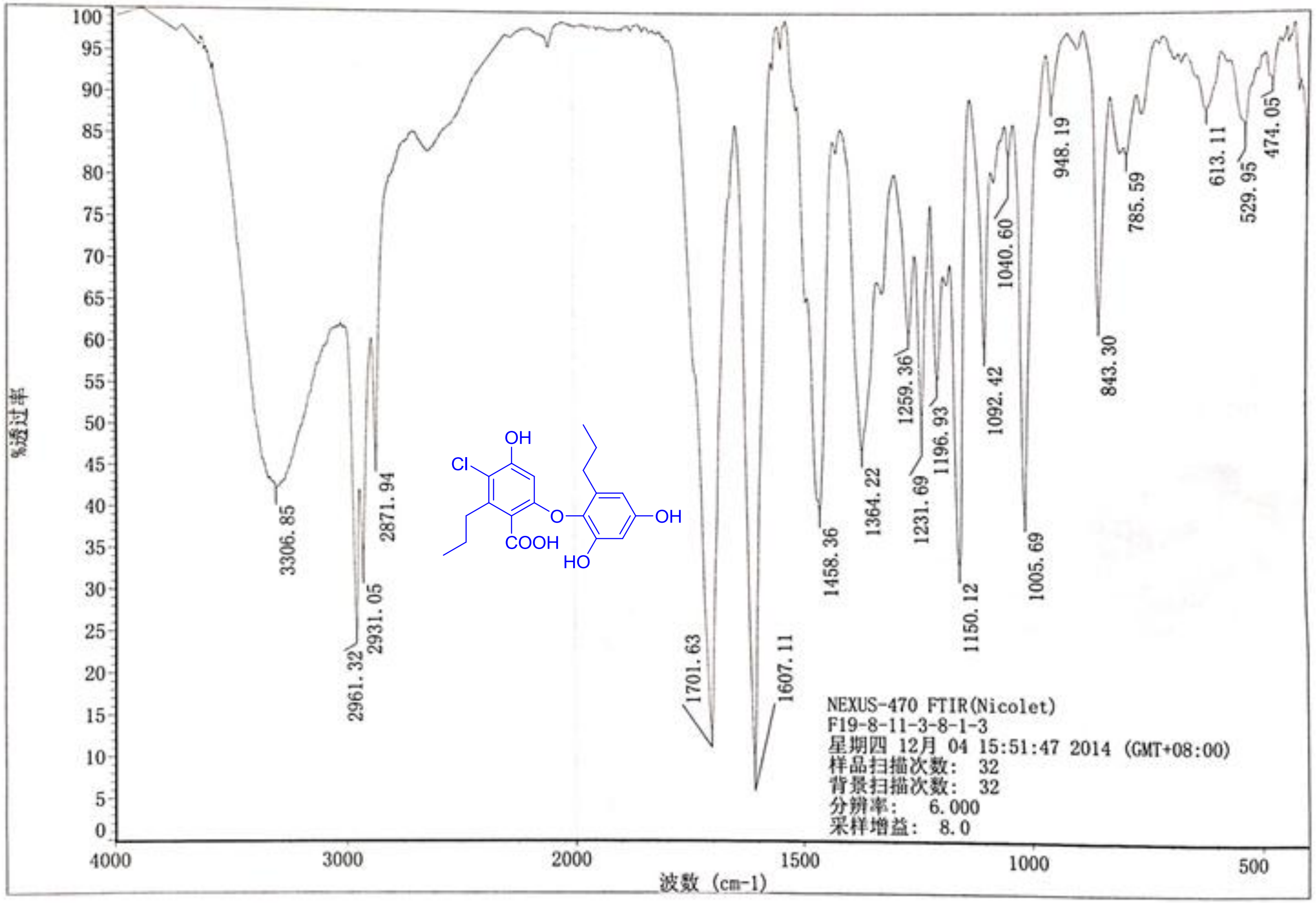

Figure S38. IR spectrum of compound **6**.

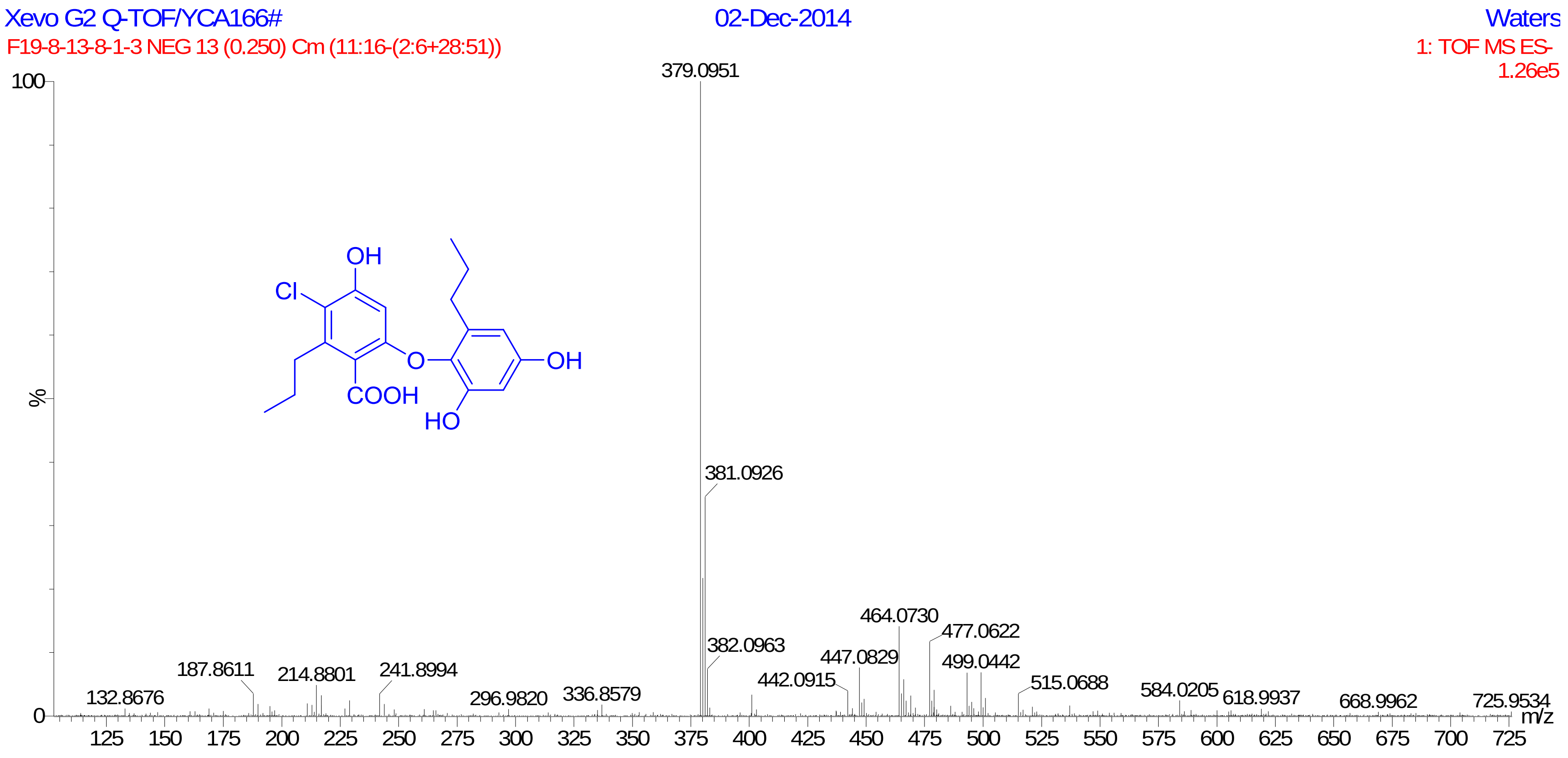

Figure S39. Negative mode HRESIMS data of compound **6**.

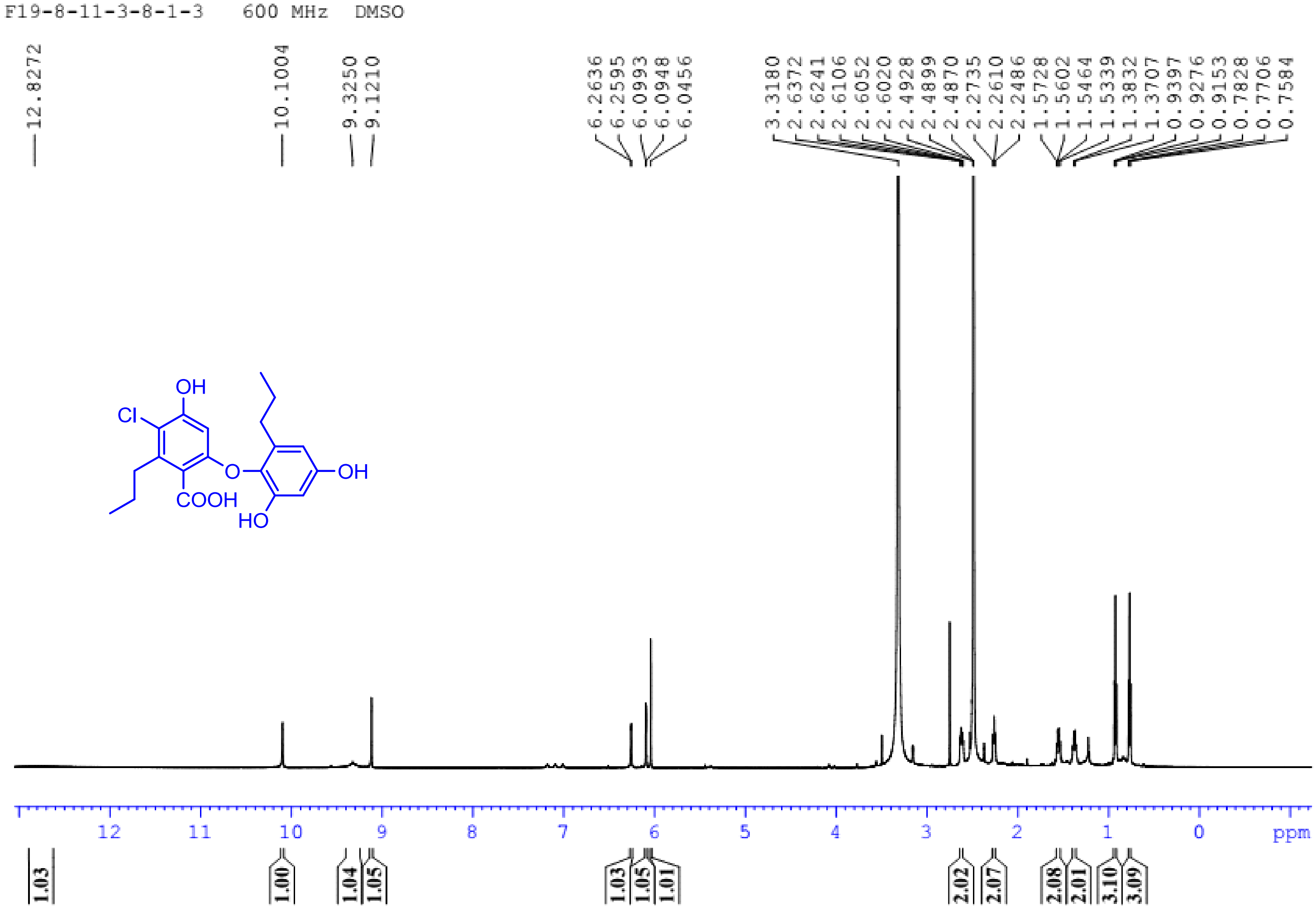

Figure S40. ^{1}H NMR spectrum of compound **6** in DMSO-d6 (600 MHz).

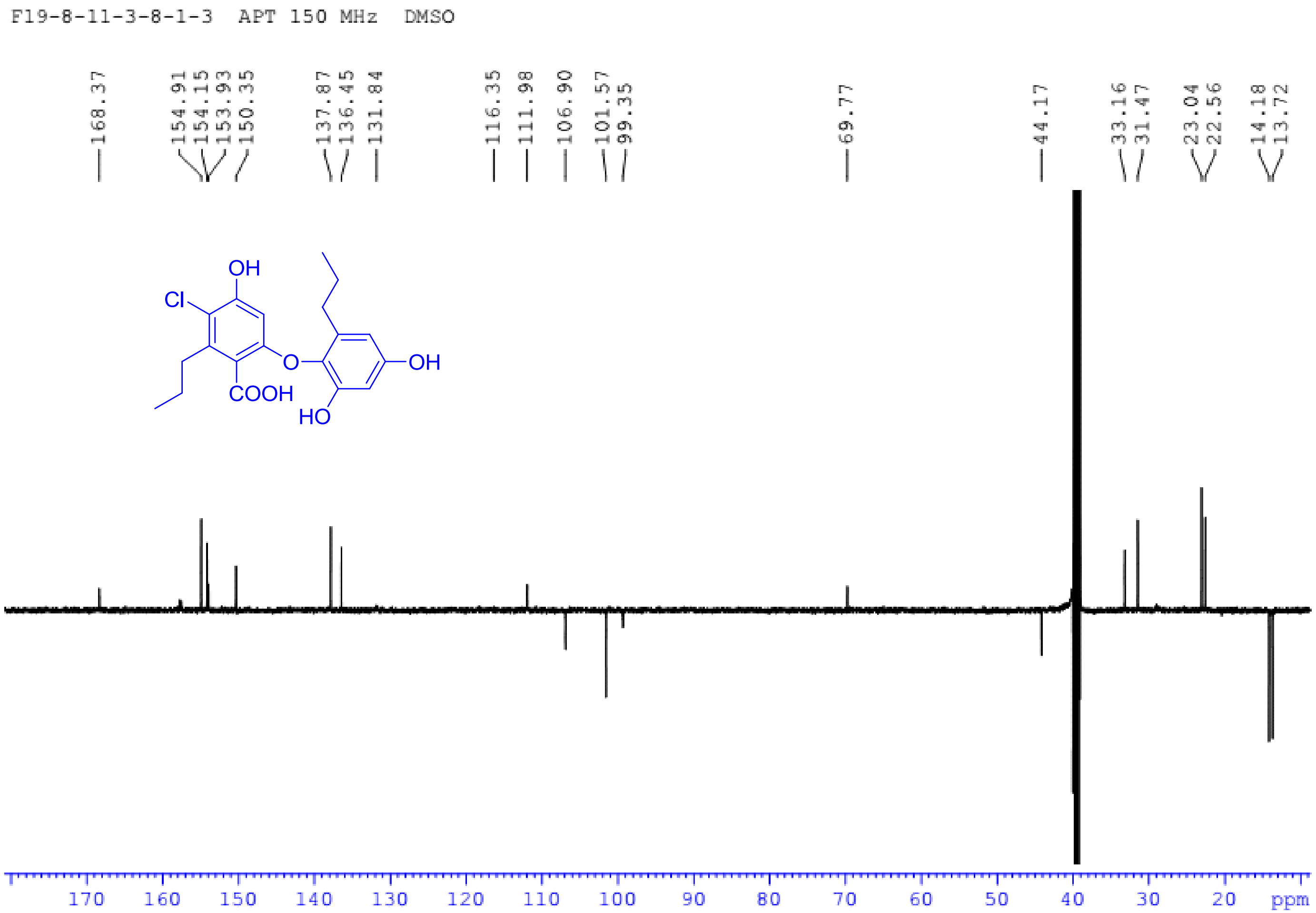

Figure S41. ^{13}C NMR spectrum of compound **6** in DMSO-d6 (150 MHz).

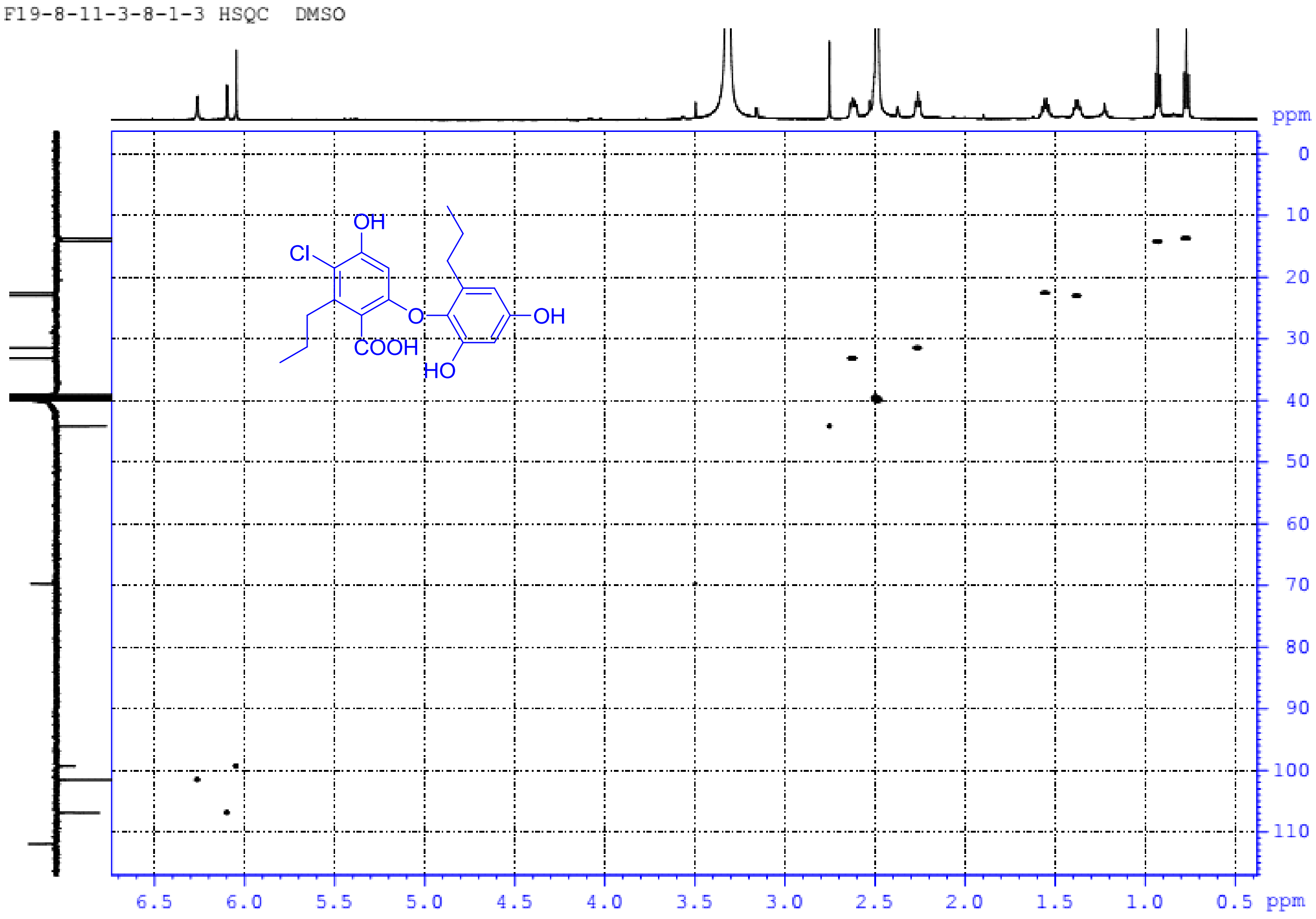

Figure S42. HSQC spectrum of compound **6** in DMSO-d6.

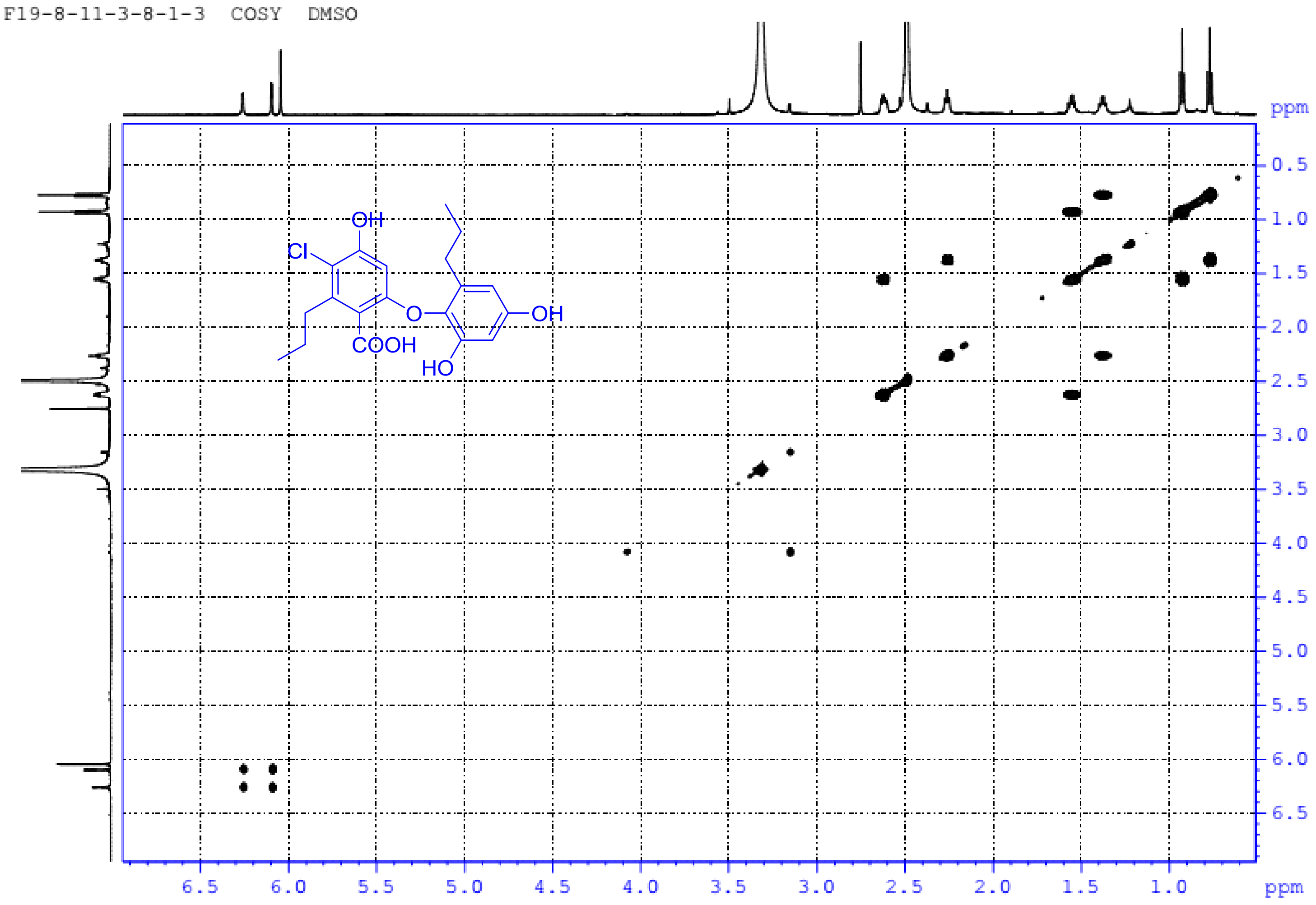

Figure S43. ^{1}H-^{1}H COSY spectrum of compound **6** in DMSO-d6.

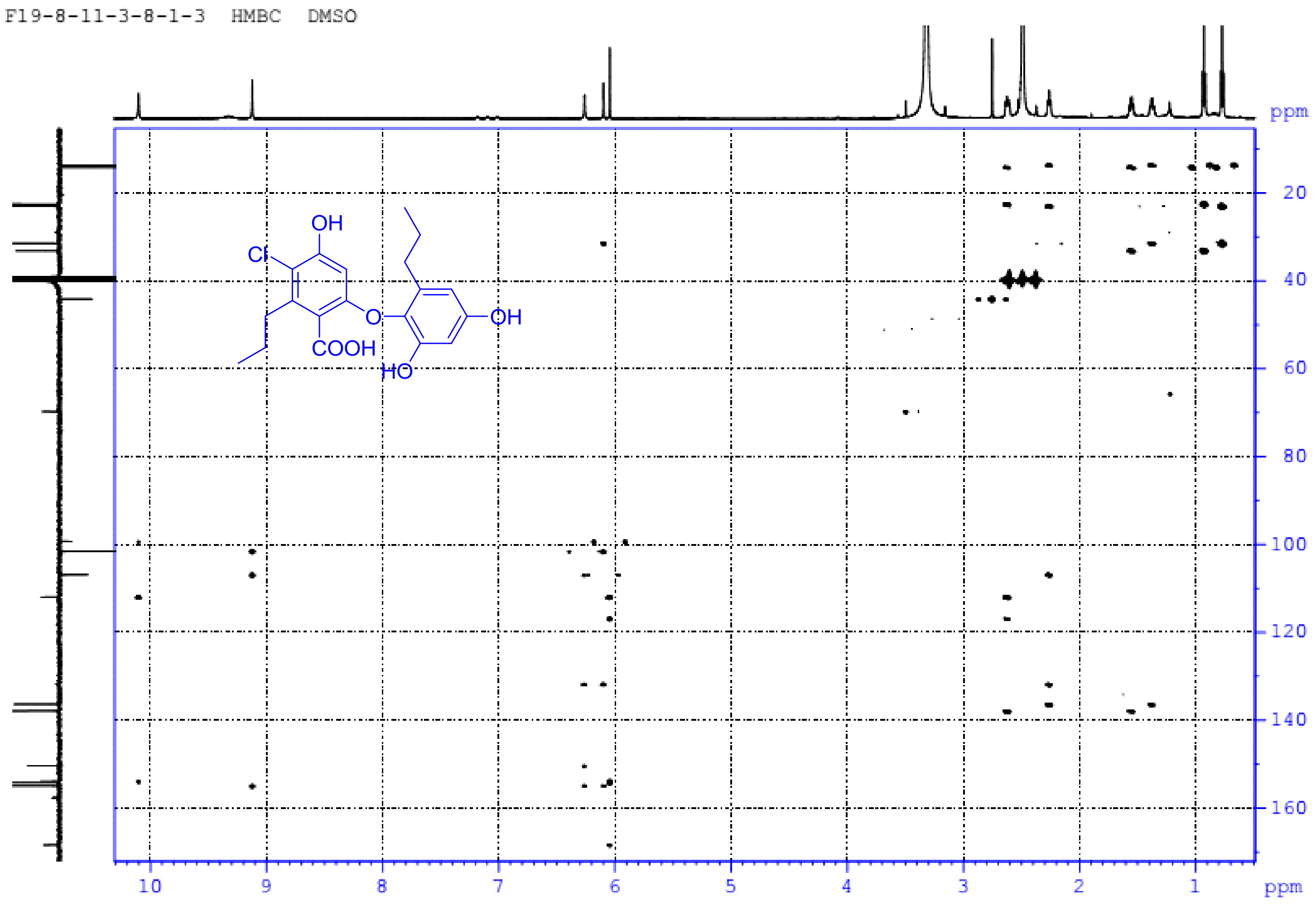

Figure S44. HMBC spectrum of compound **6** in DMSO-d6.

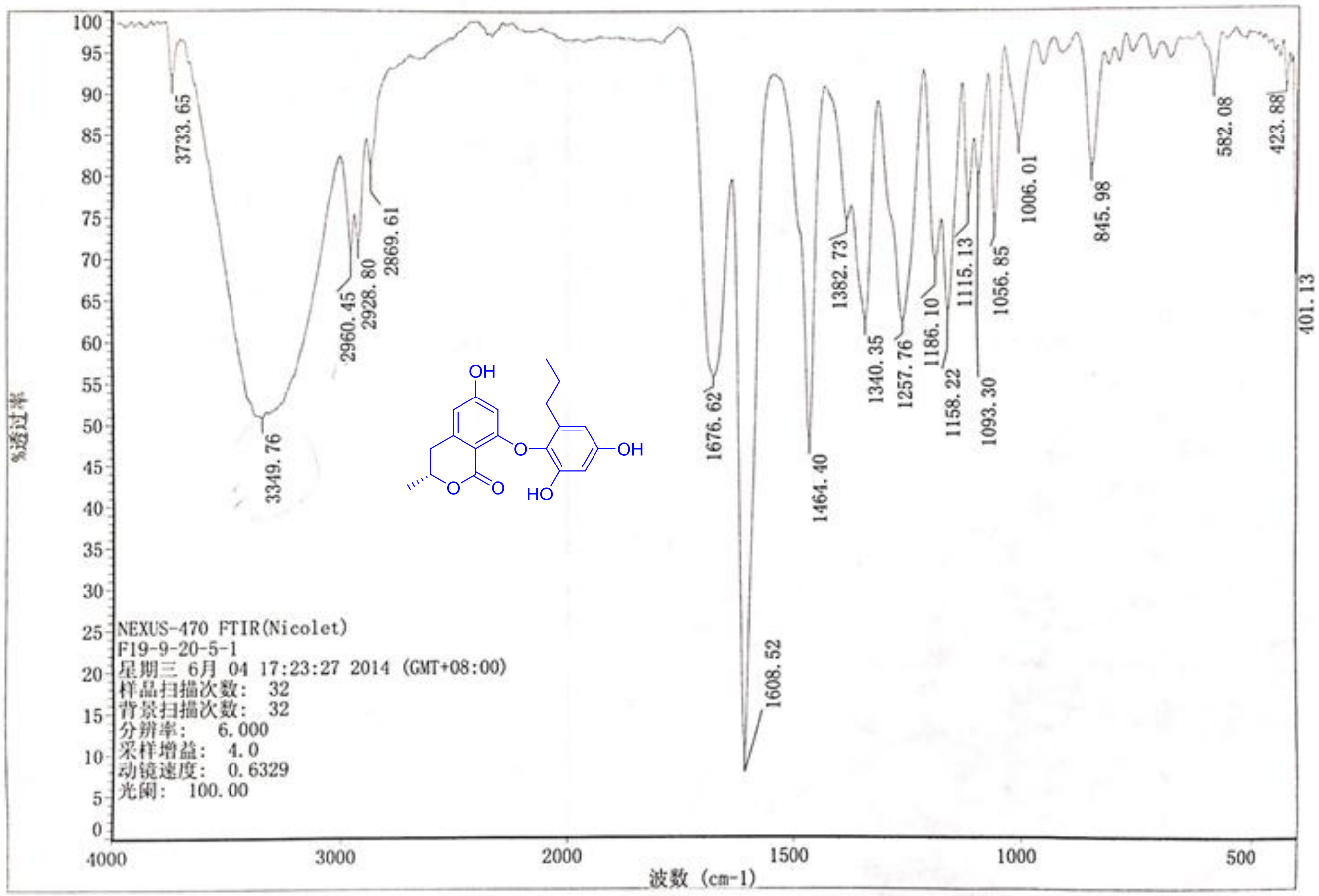

Figure S45. IR spectrum of compound **7**.

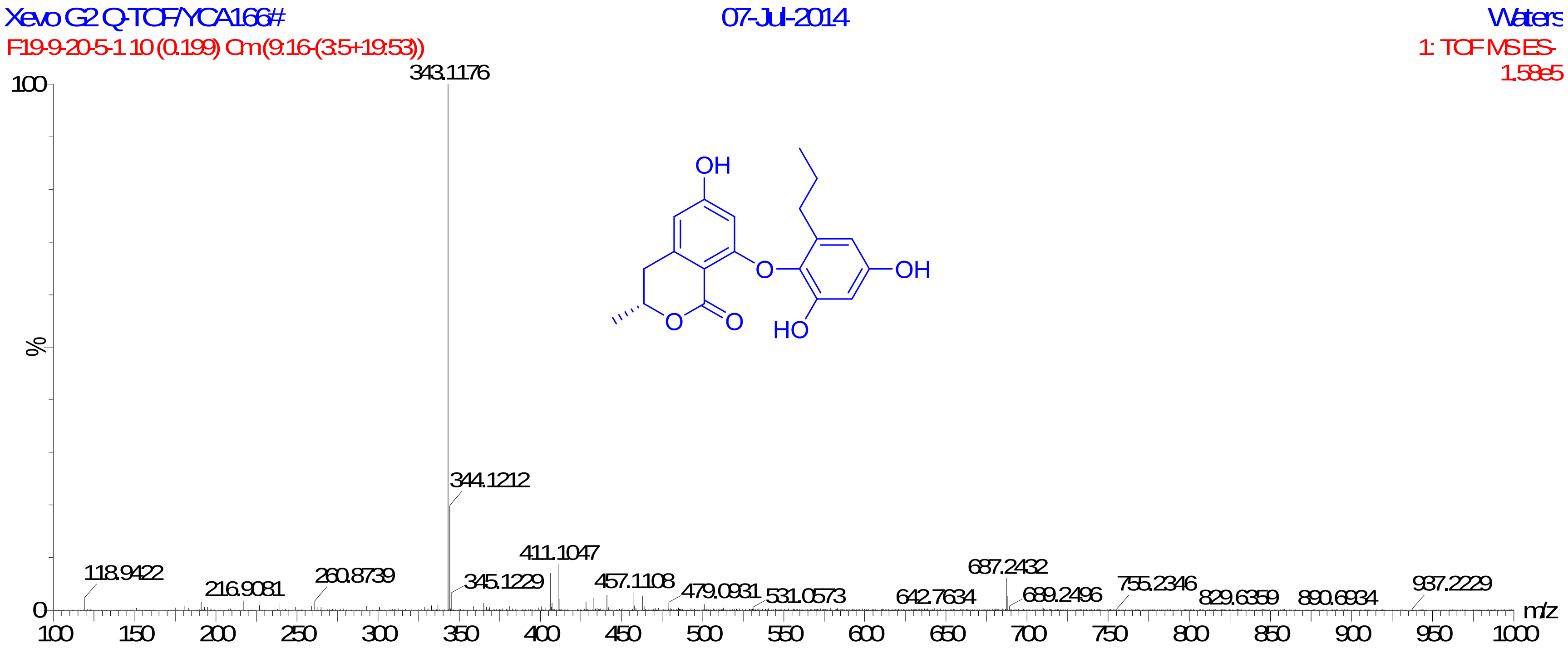

Figure S46. Negative mode HRESIMS data of compound **7**.

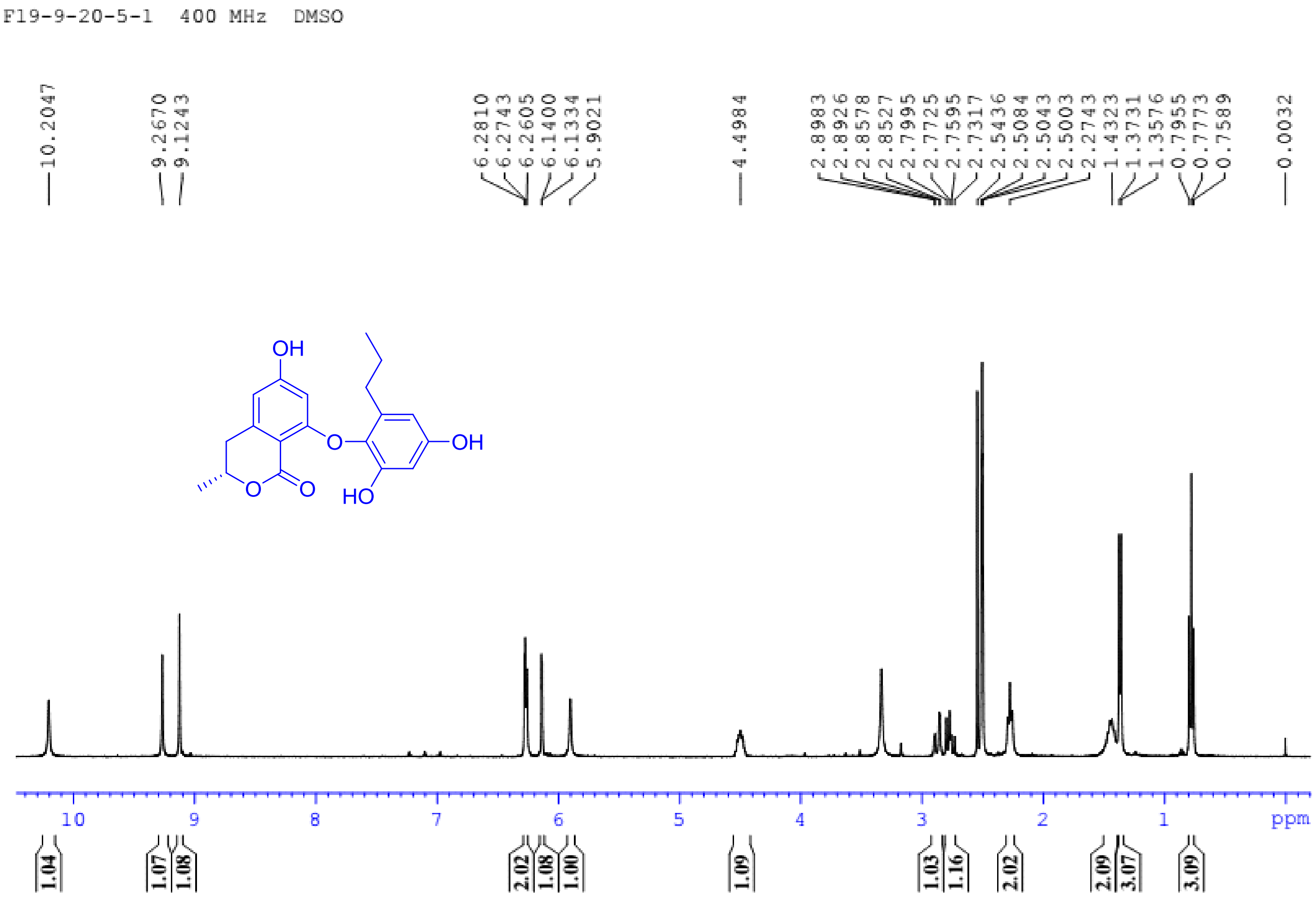

Figure S47. ^{1}H NMR spectrum of compound **7** in DMSO-d6 (400 MHz).

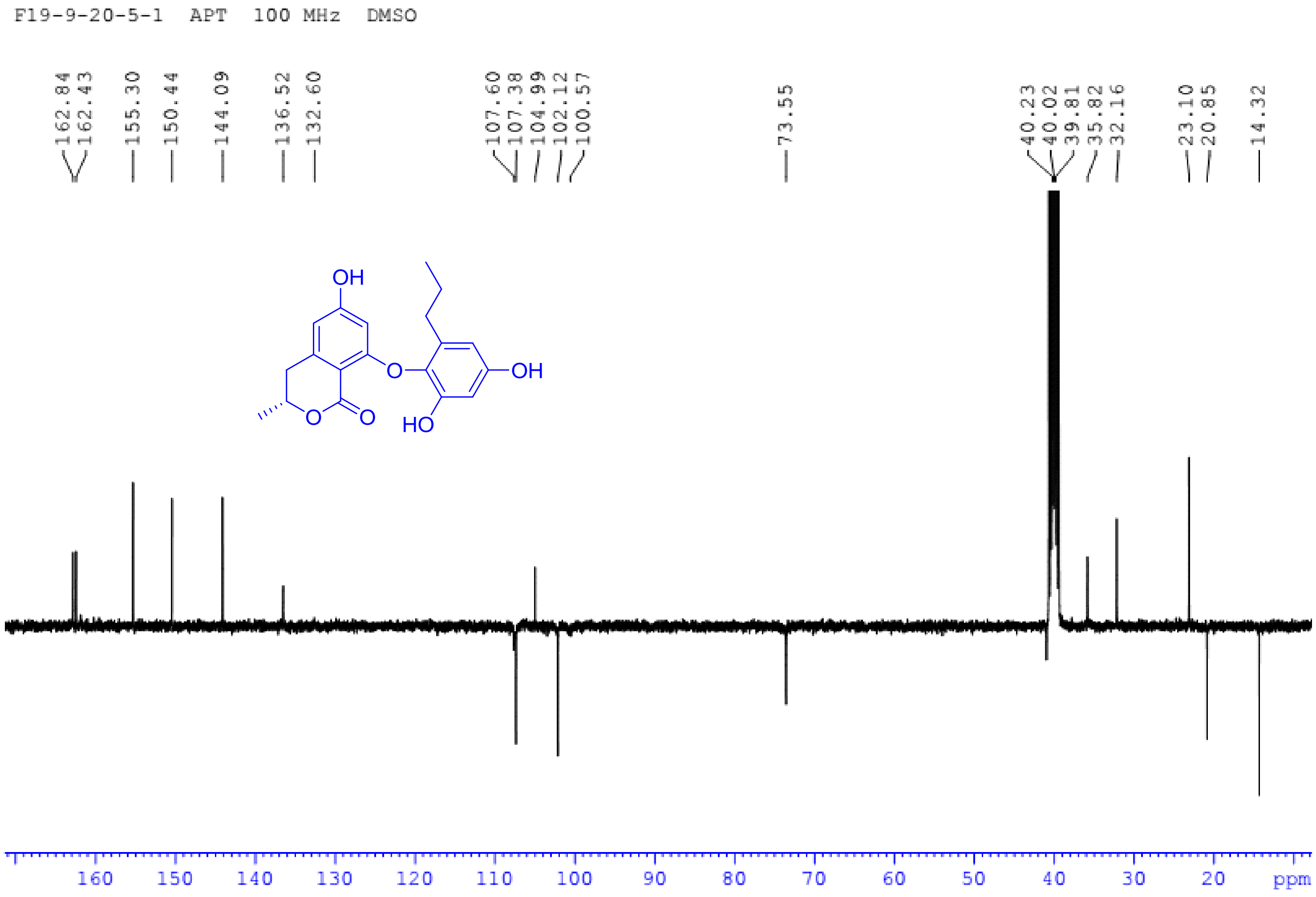

Figure S48. ^{13}C NMR spectrum of compound **7** in DMSO-d6 (100 MHz).

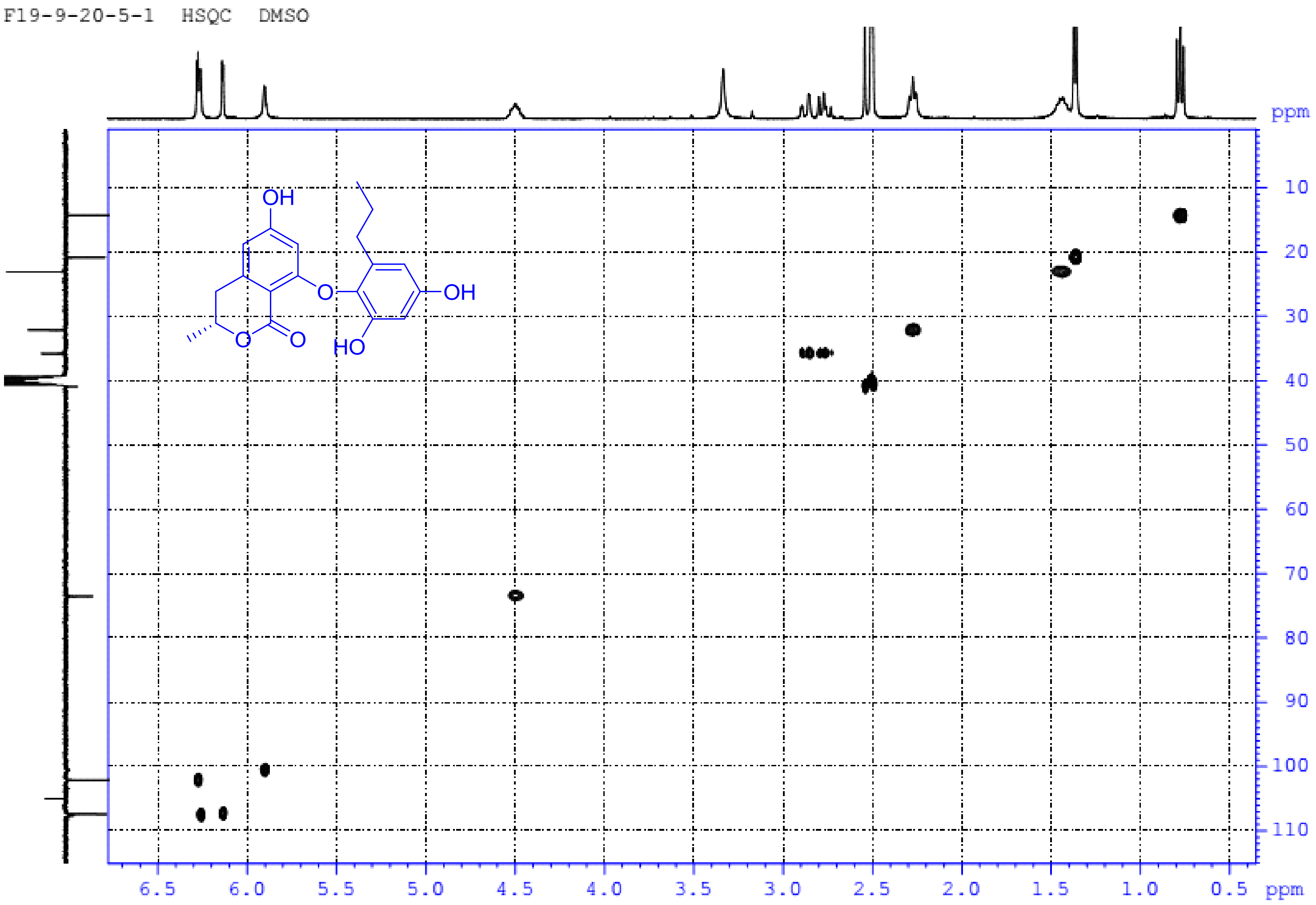

Figure S49. HSQC spectrum of compound **7** in DMSO-d6.

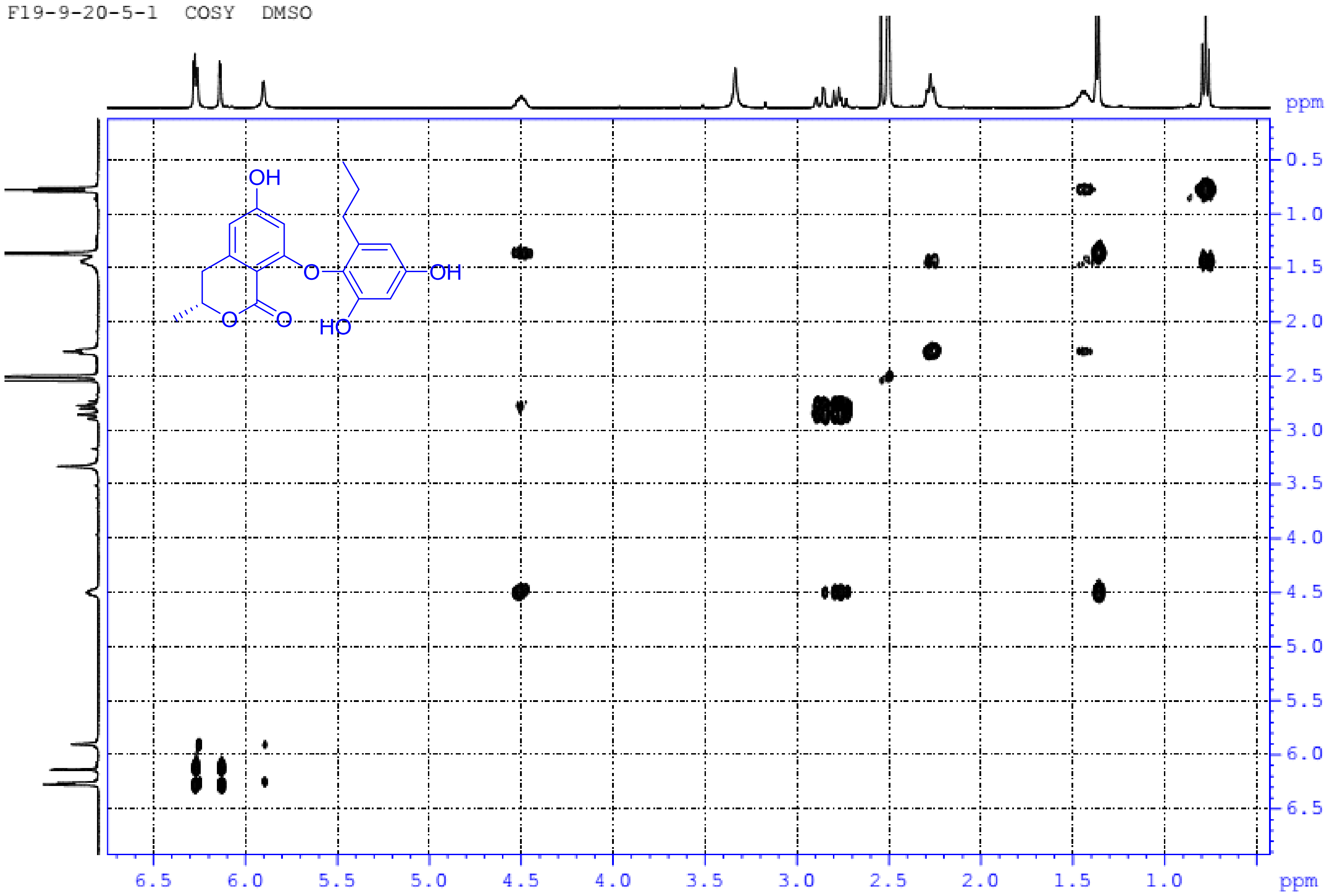

Figure S50. ^{1}H-^{1}H COSY spectrum of compound **7** in DMSO-d6.

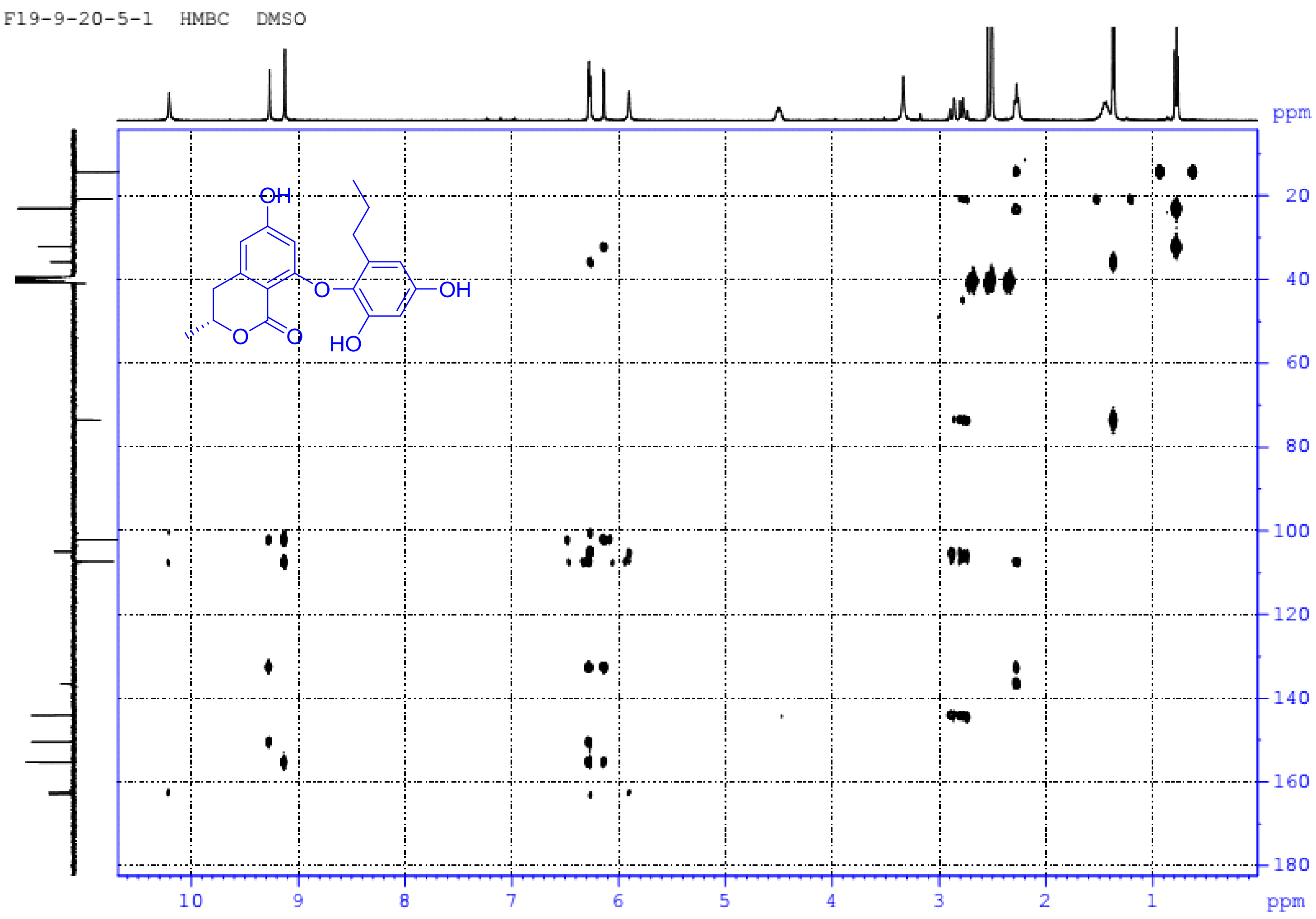

Figure S51. HMBC spectrum of compound **7** in DMSO-d6.

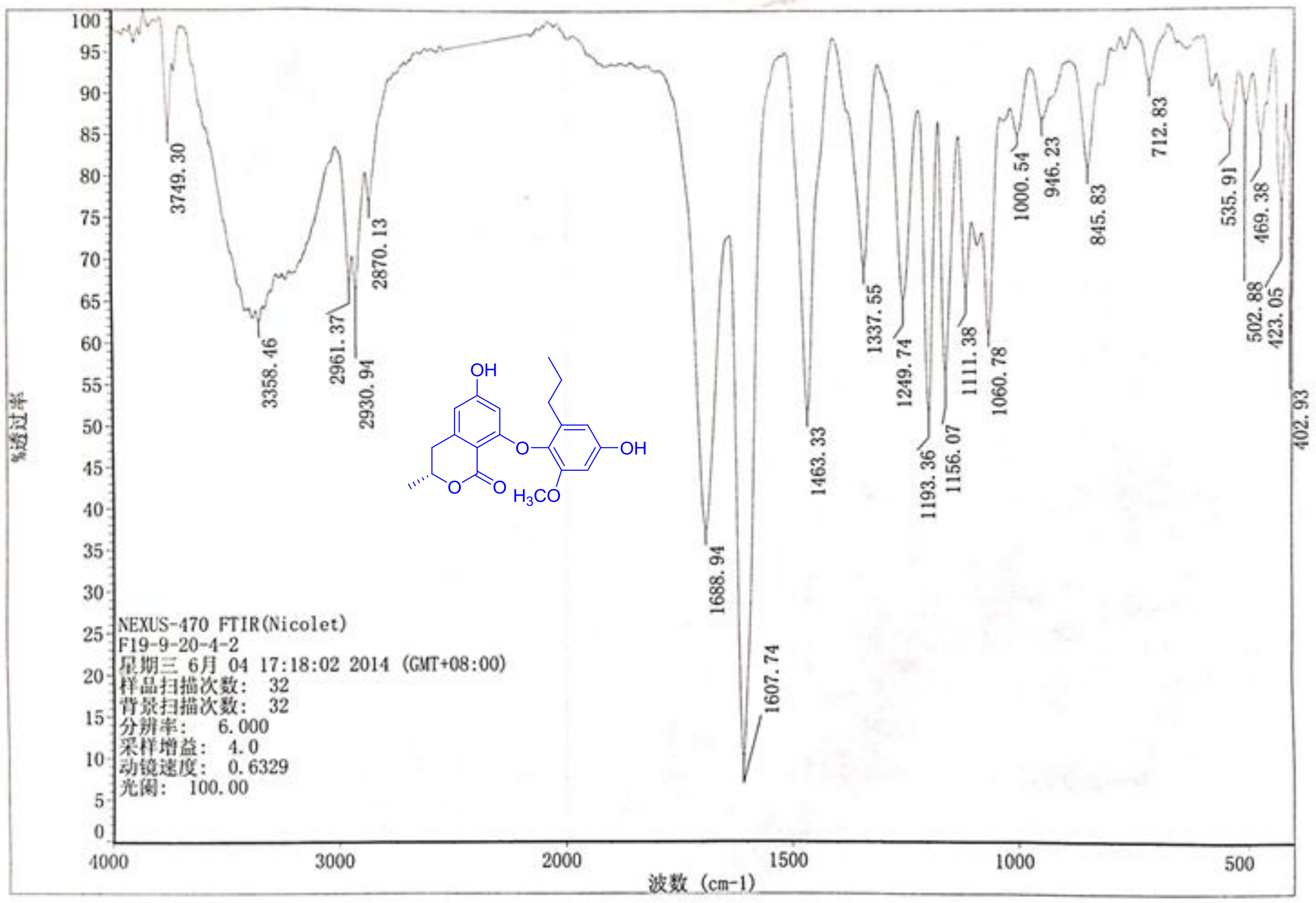

Figure S52. IR spectrum of compound **8**.

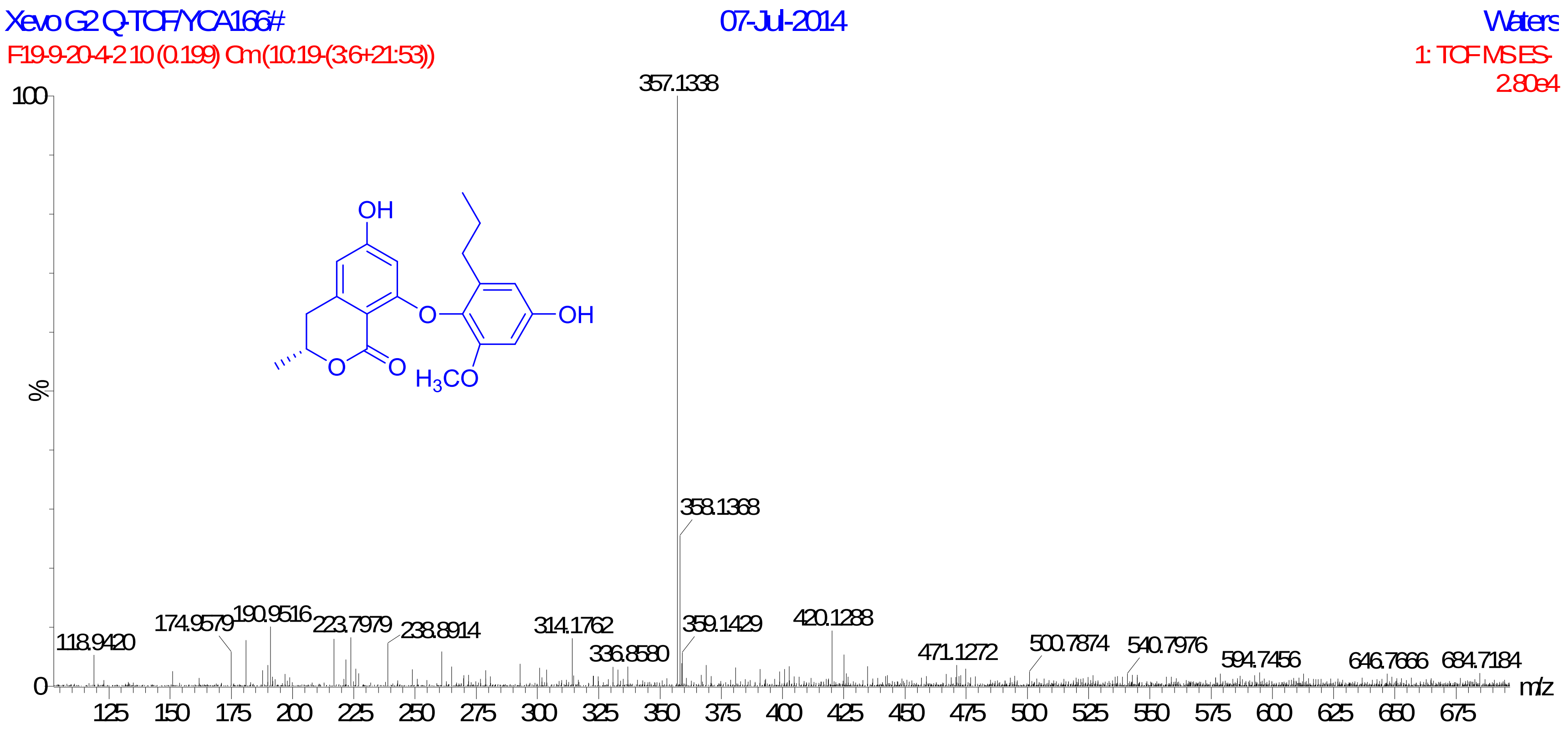

Figure S53. Negative mode HRESIMS data of compound **8**.

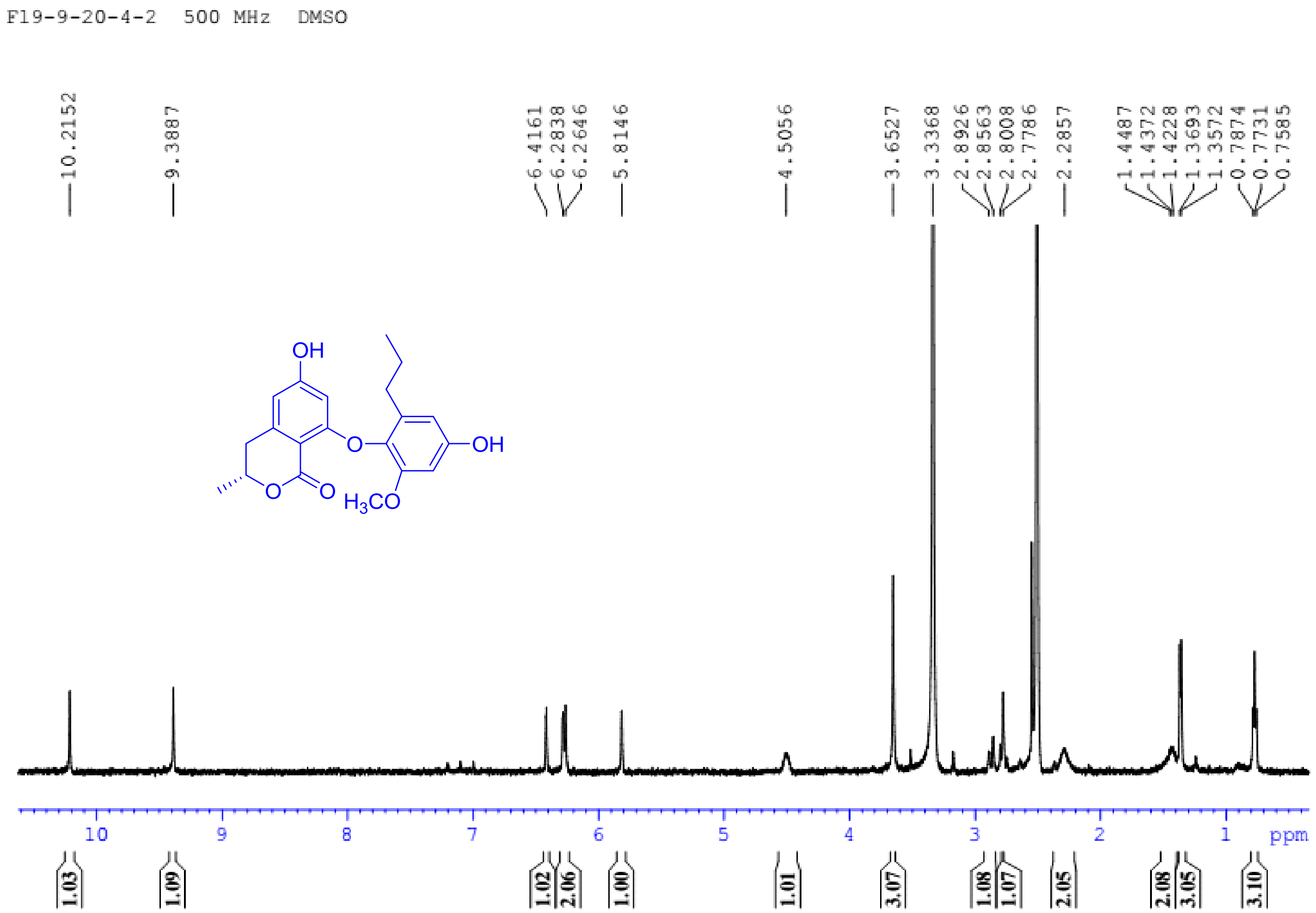

Figure S54. ^{1}H NMR spectrum of compound **8** in DMSO-d6 (500 MHz).

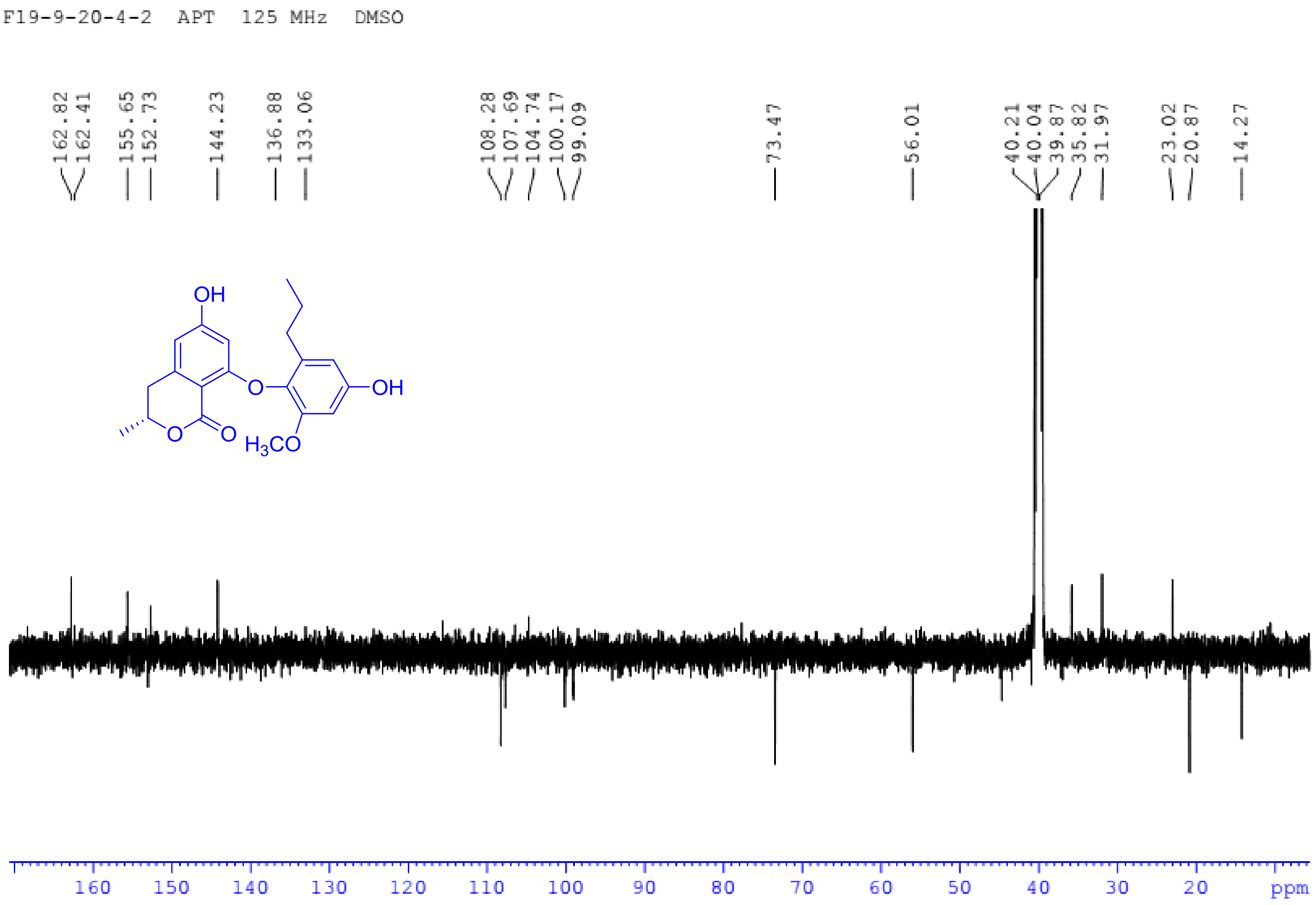

Figure S55. ^{13}C NMR spectrum of compound **8** in DMSO-d6 (125 MHz).

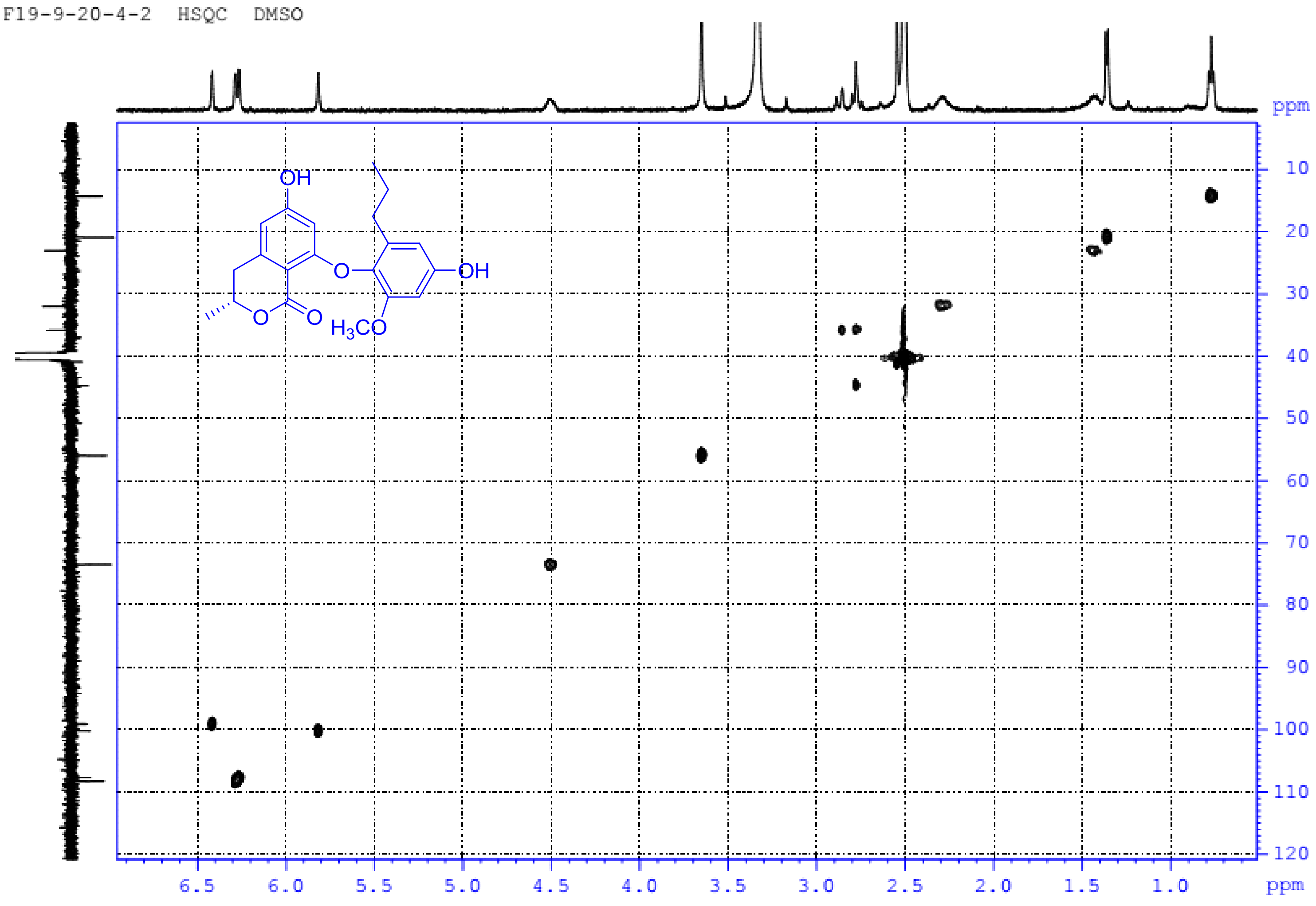

Figure S56. HSQC spectrum of compound **8** in DMSO-d6.

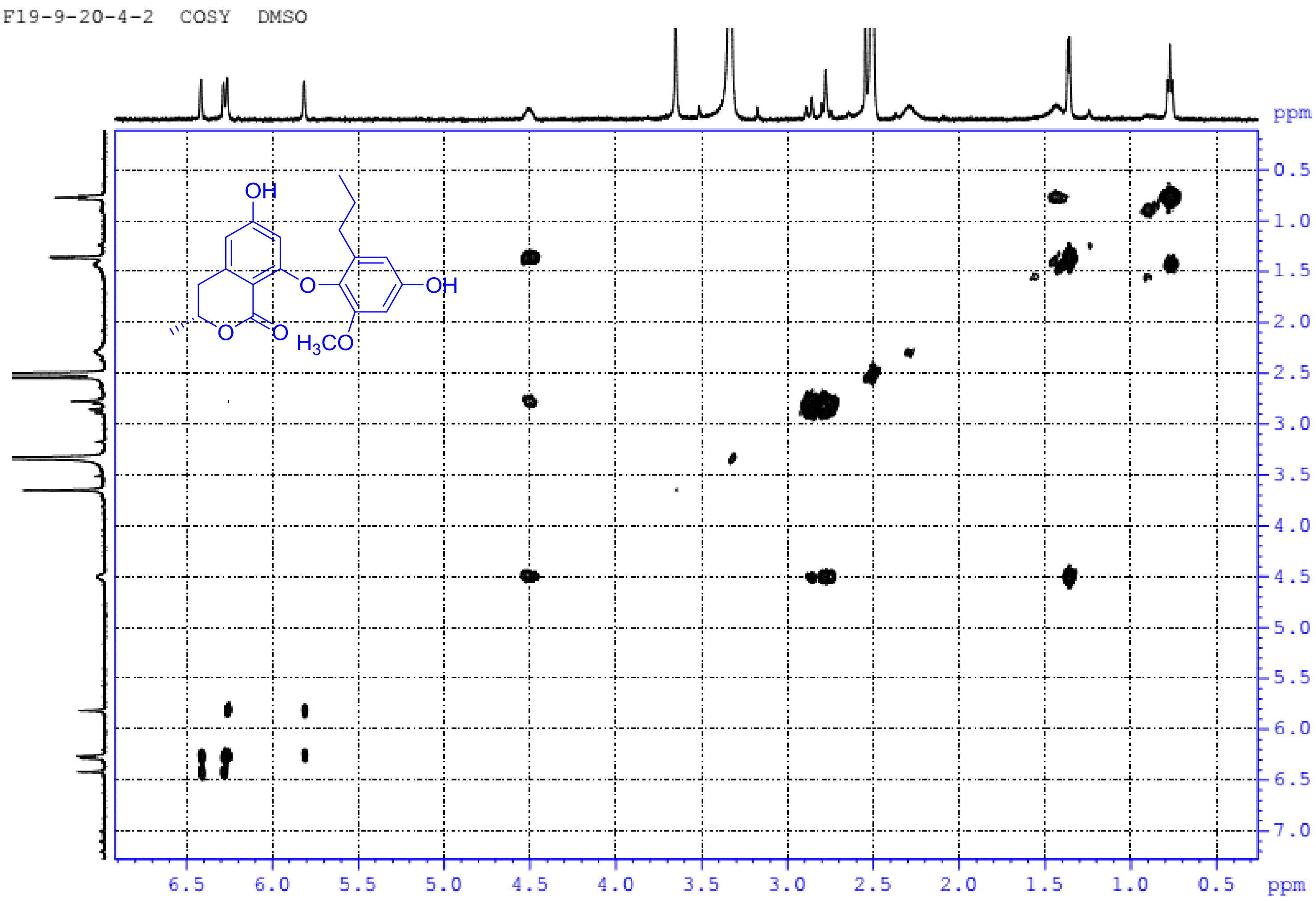

Figure S57. ^{1}H-^{1}H COSY spectrum of compound **8** in DMSO-d6.

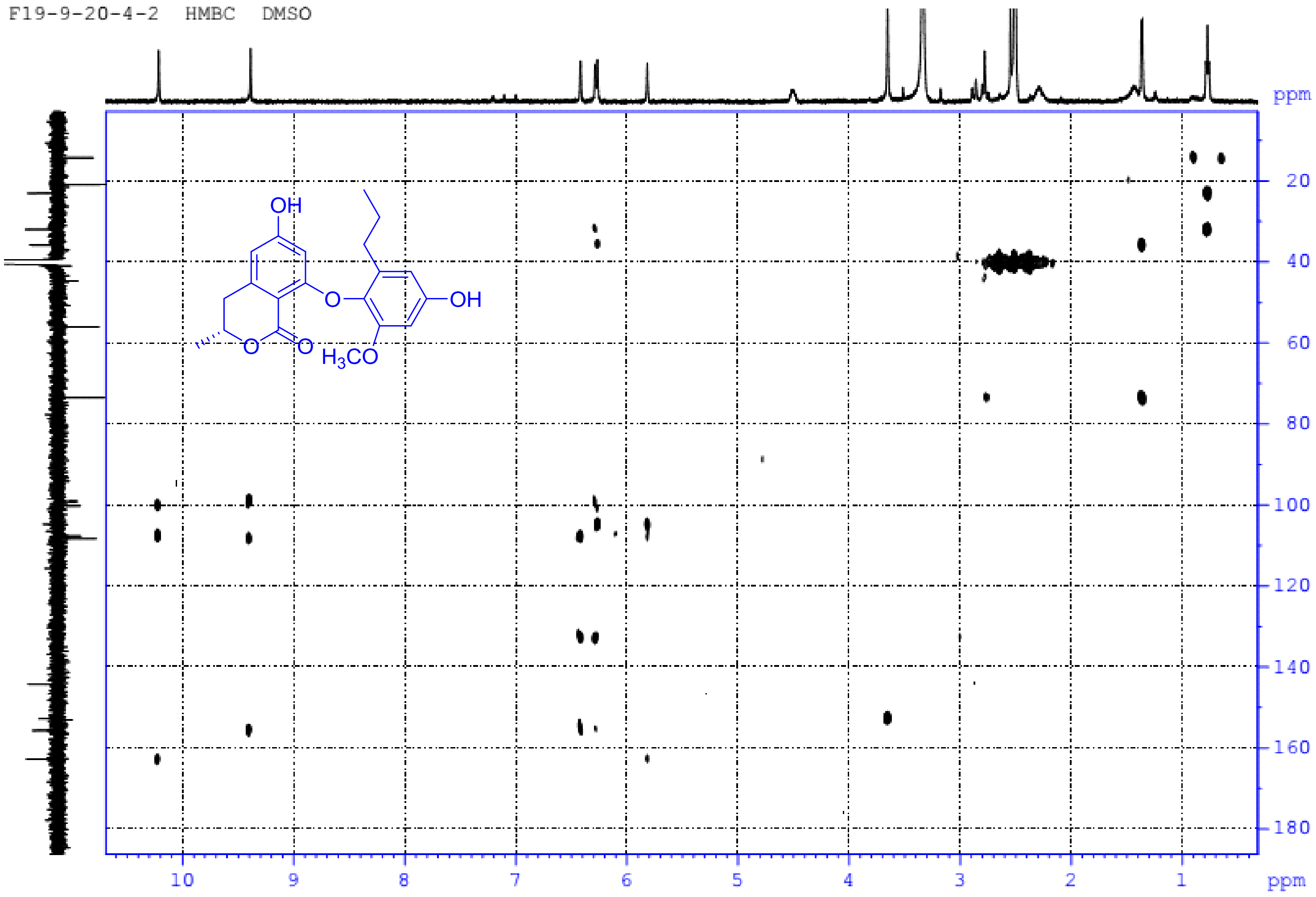

Figure S58. HMBC spectrum of compound **8** in DMSO-d6.

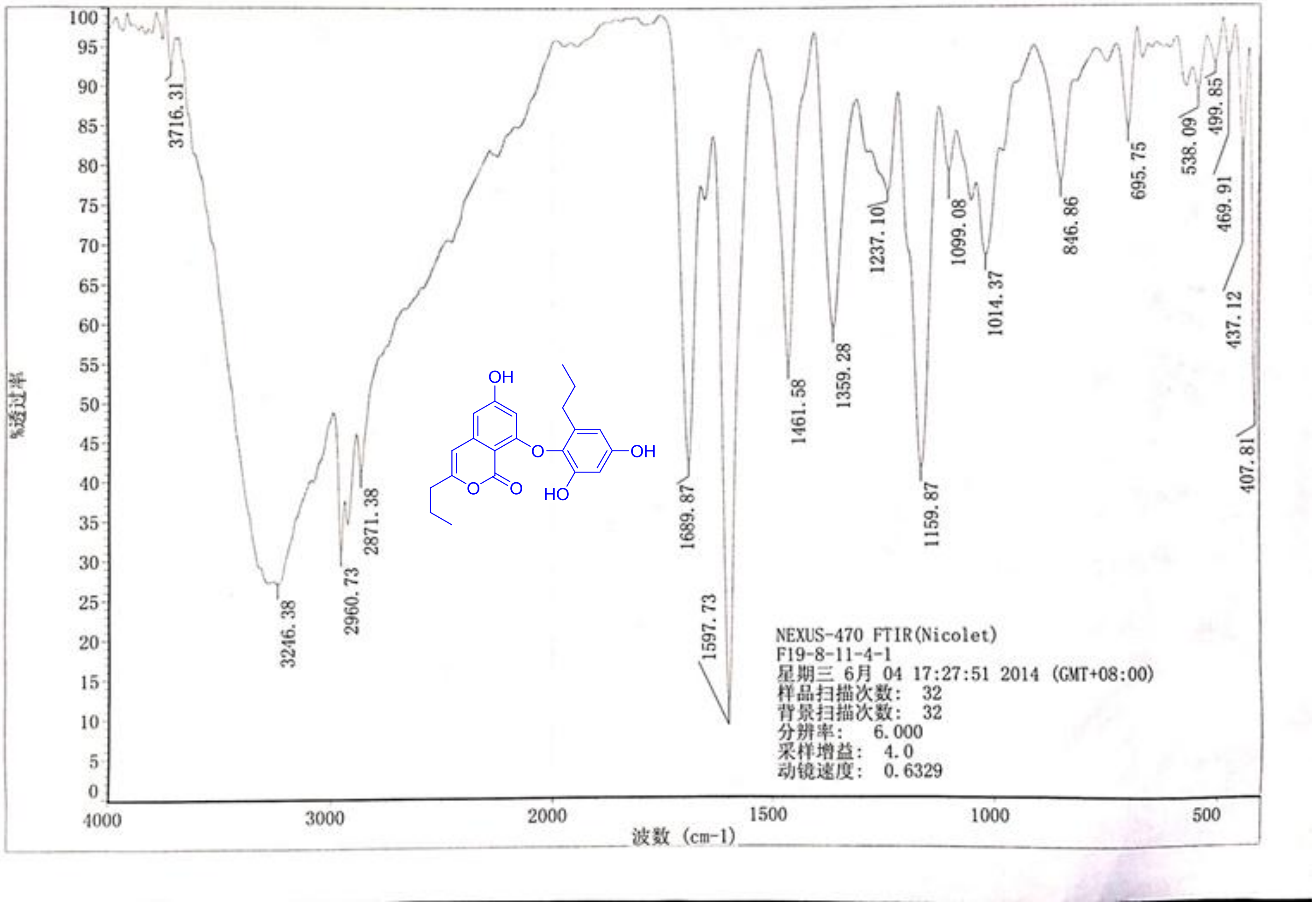

Figure S59. IR spectrum of compound **9**.

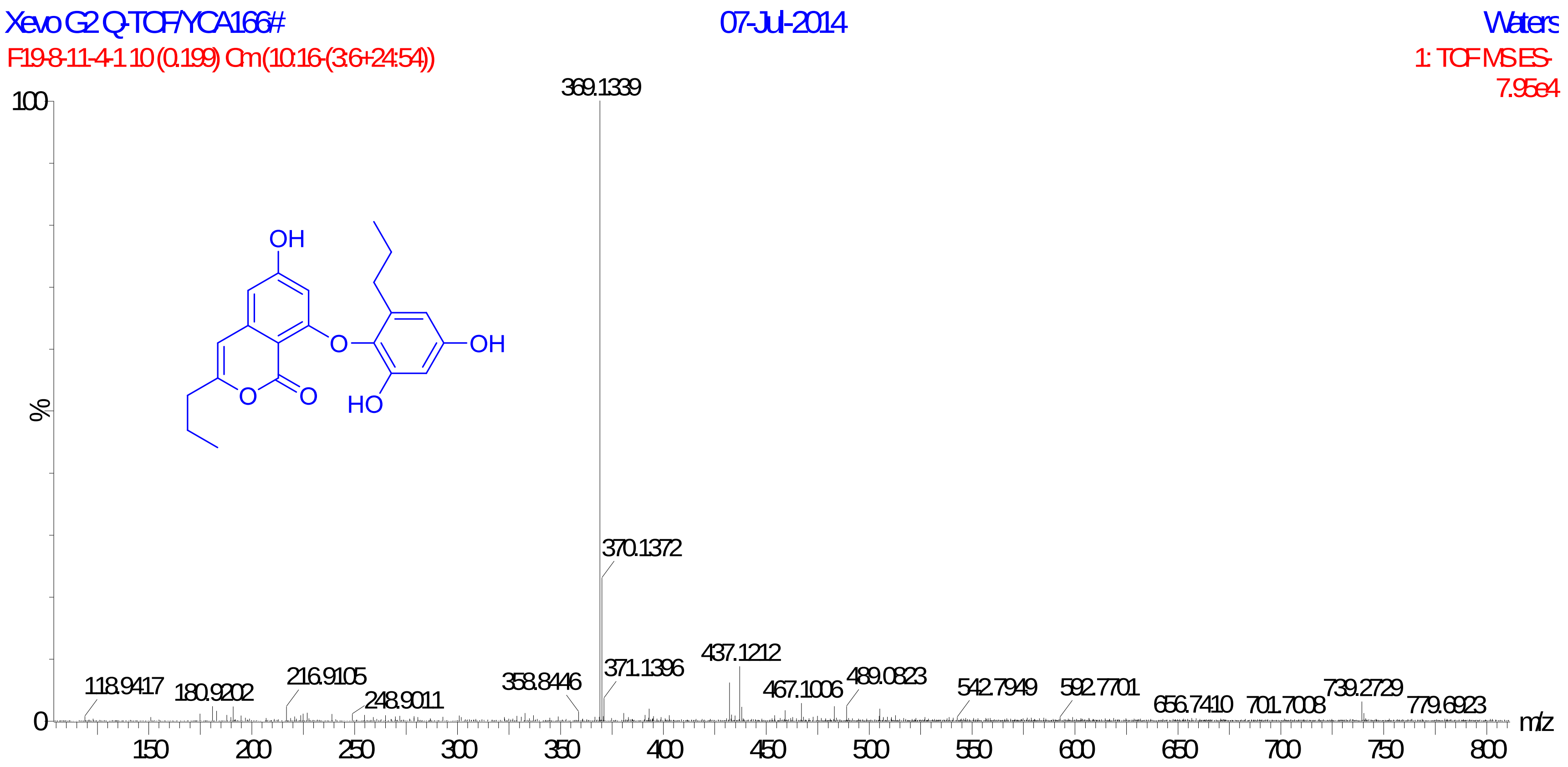

Figure S60. Negative mode HRESIMS data of compound **9**.

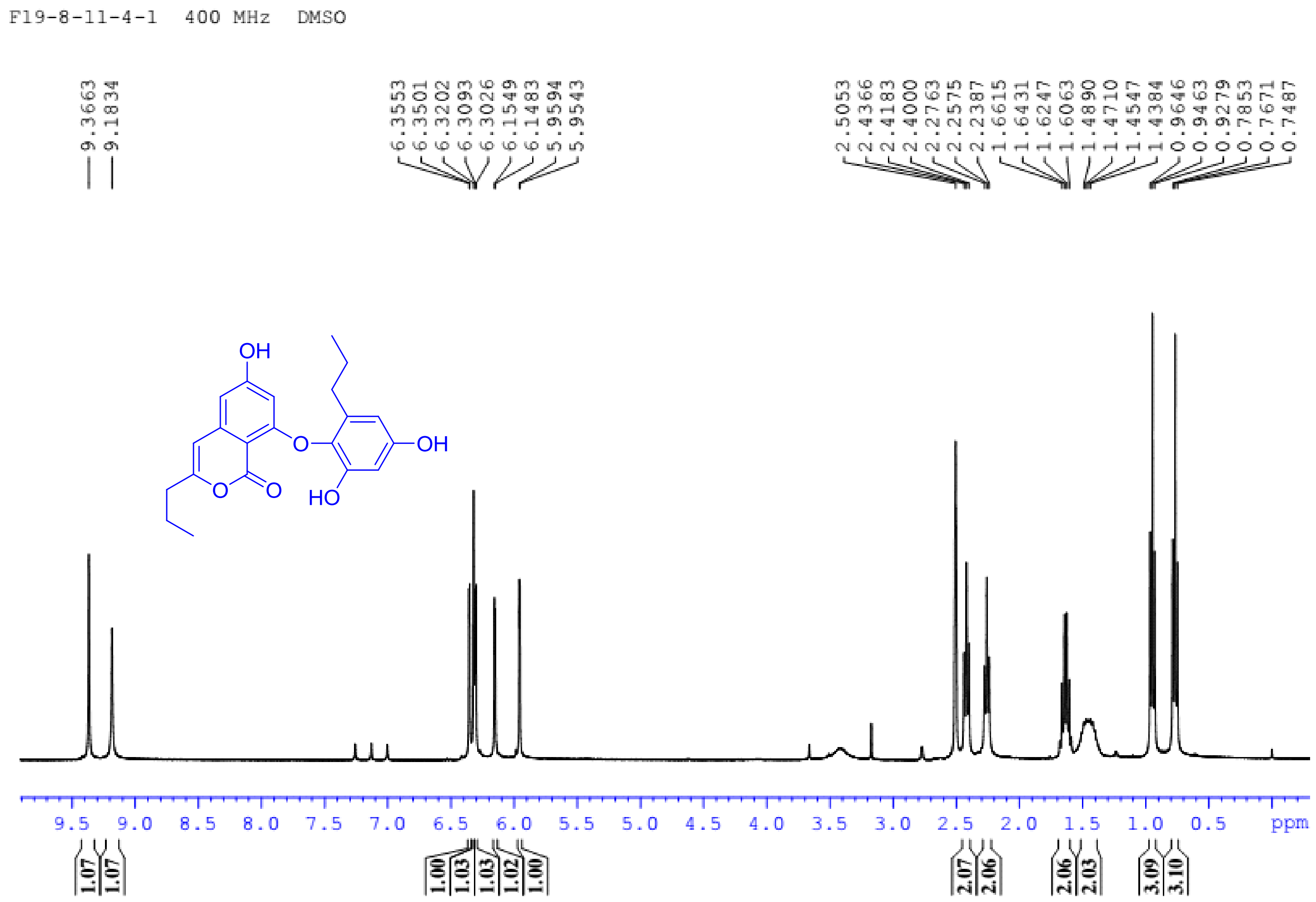

Figure S61. ^{1}H NMR spectrum of compound **9** in DMSO-d6 (400 MHz).

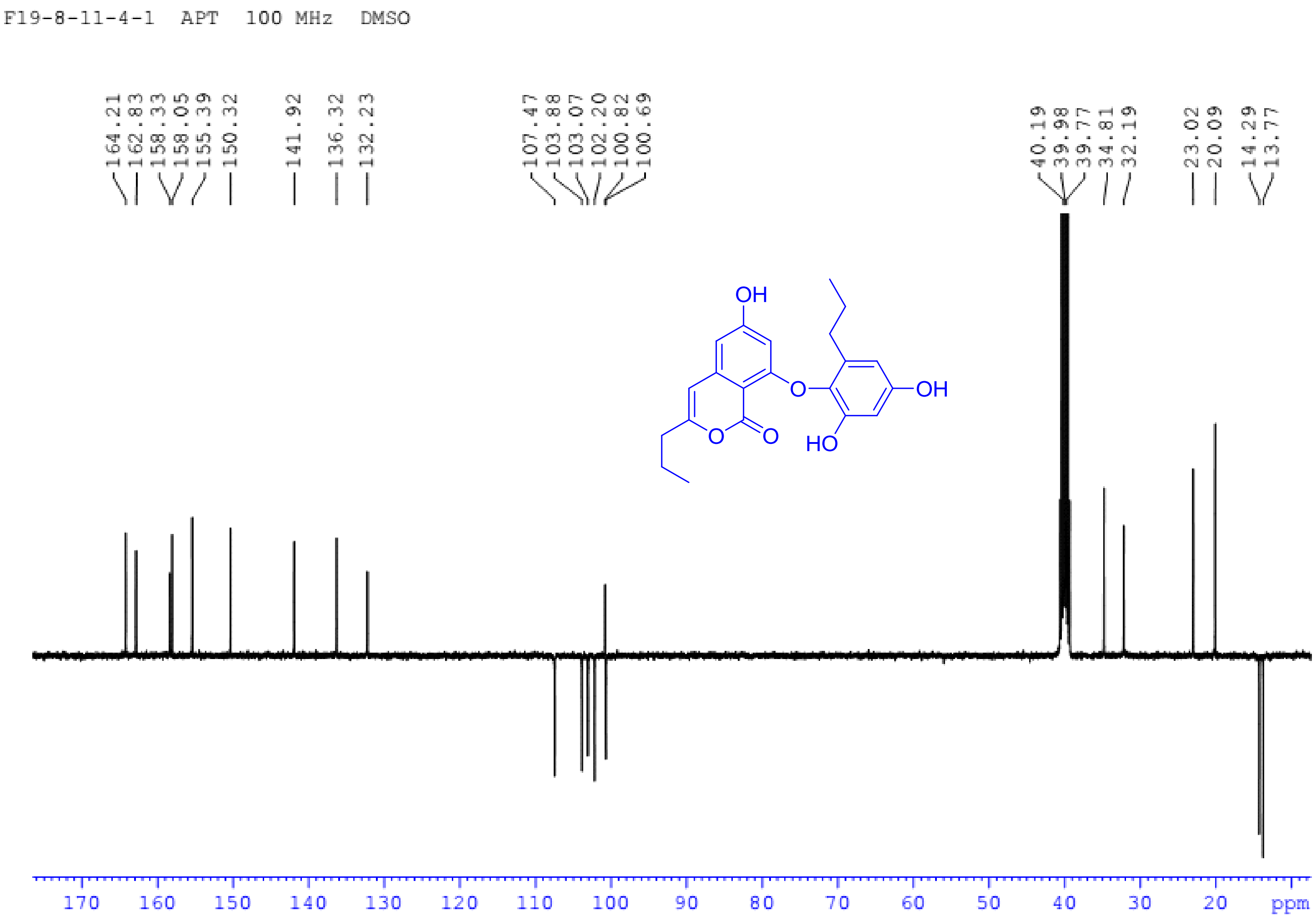

Figure S62. ^{13}C NMR spectrum of compound **9** in DMSO-d6 (100 MHz).

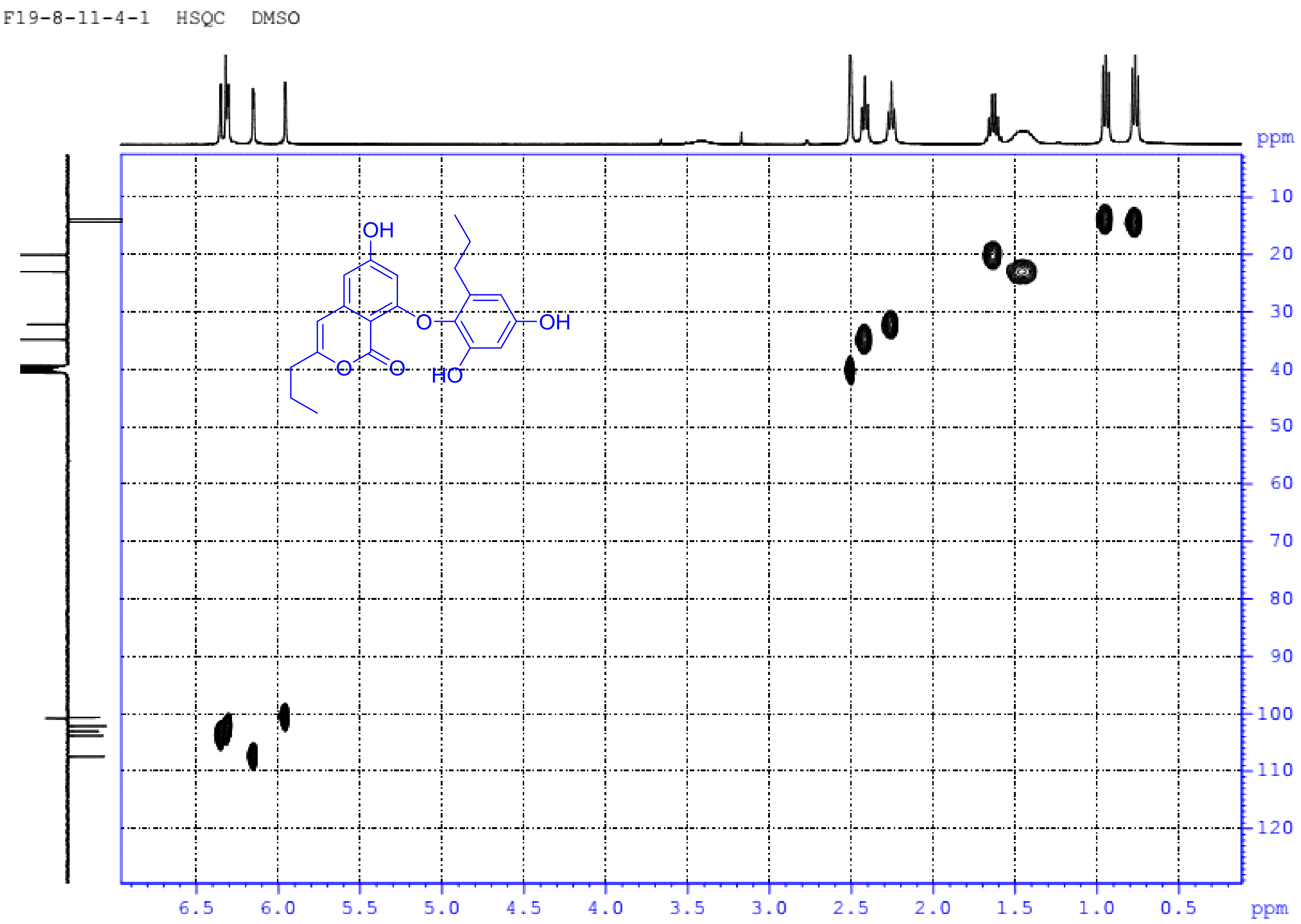

Figure S63. HSQC spectrum of compound **9** in DMSO-d6.

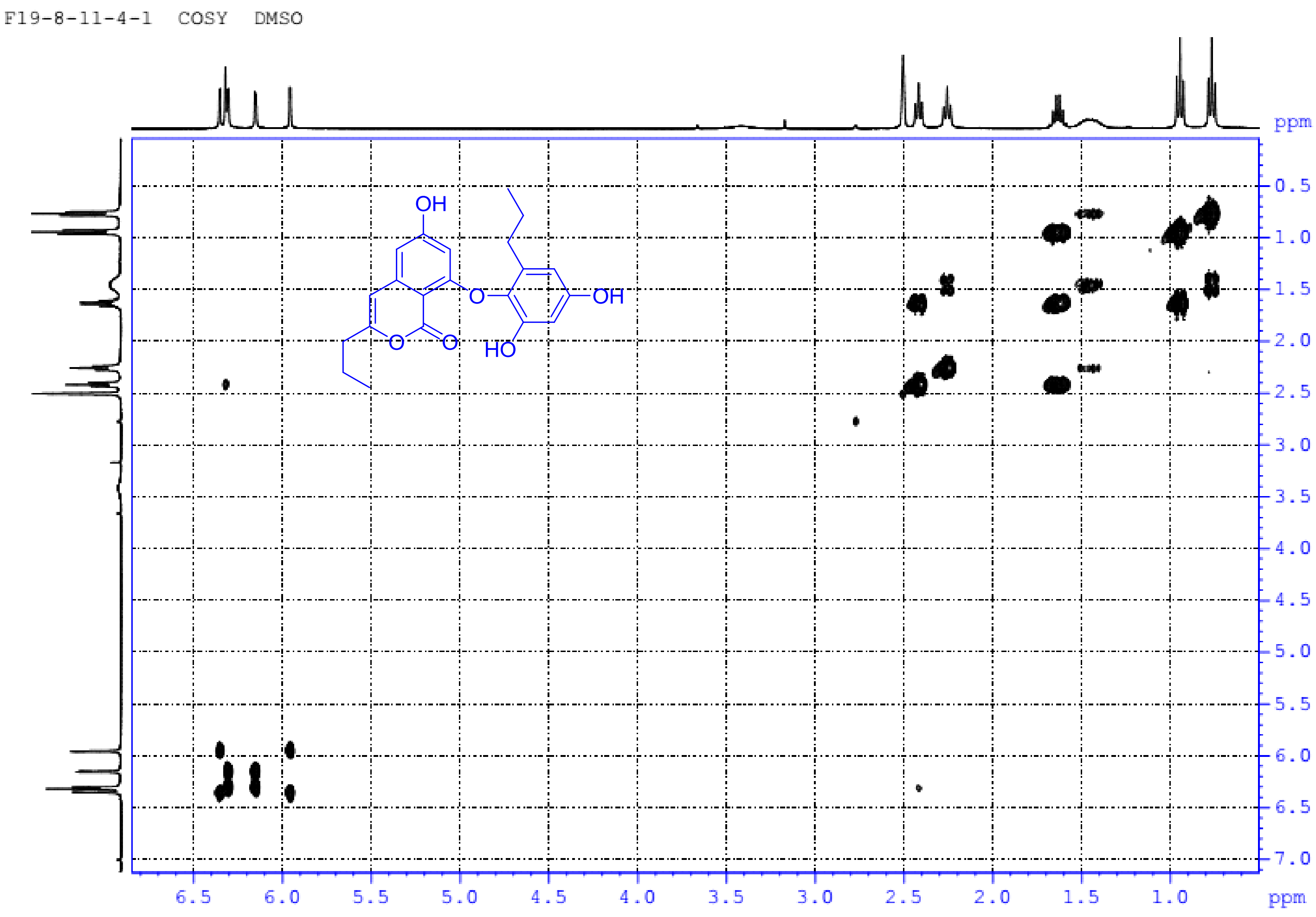

Figure S64. ^{1}H-^{1}H COSY spectrum of compound **9** in DMSO-d6.

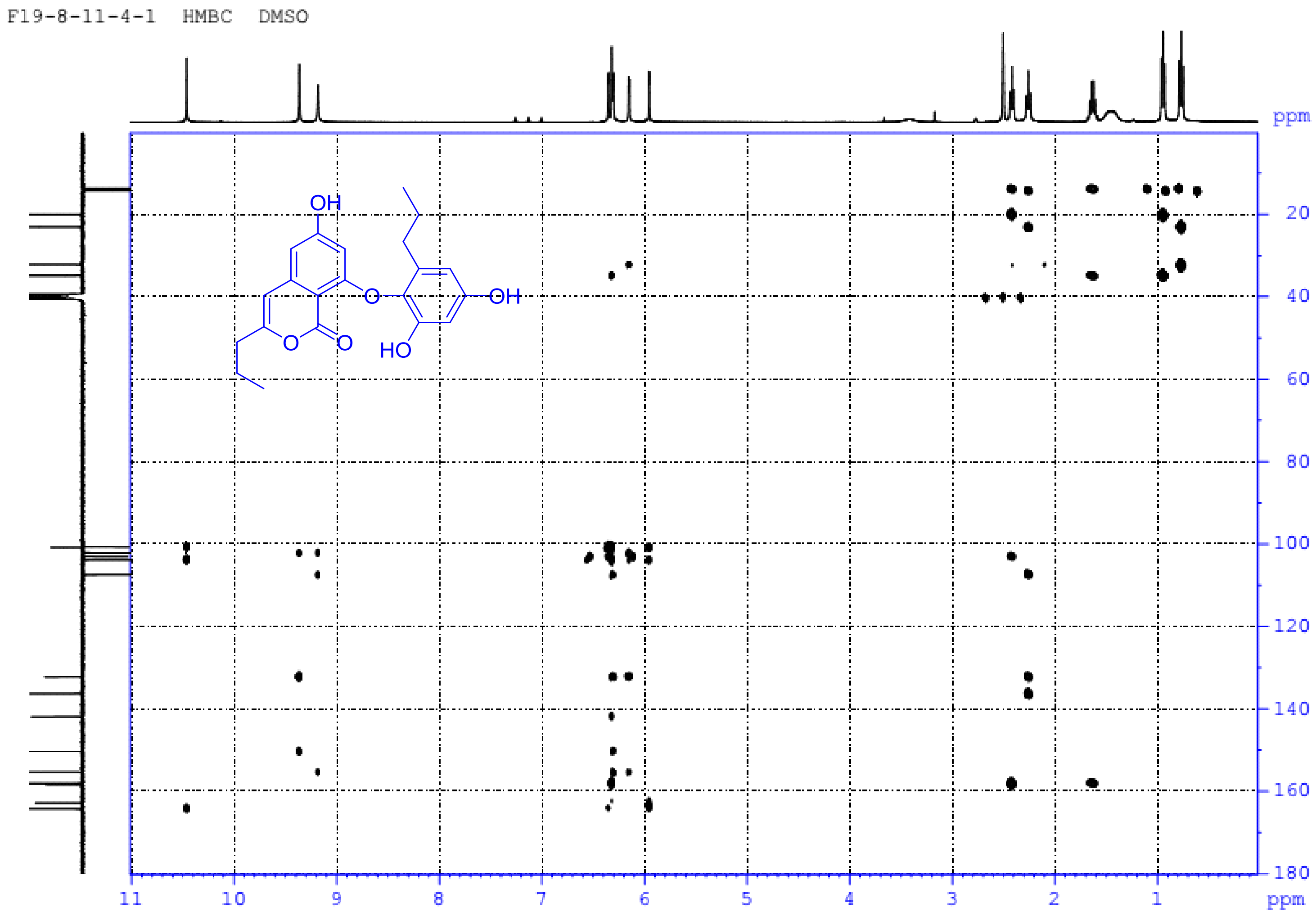

Figure S65. HMBC spectrum of compound **9** in DMSO-d6.

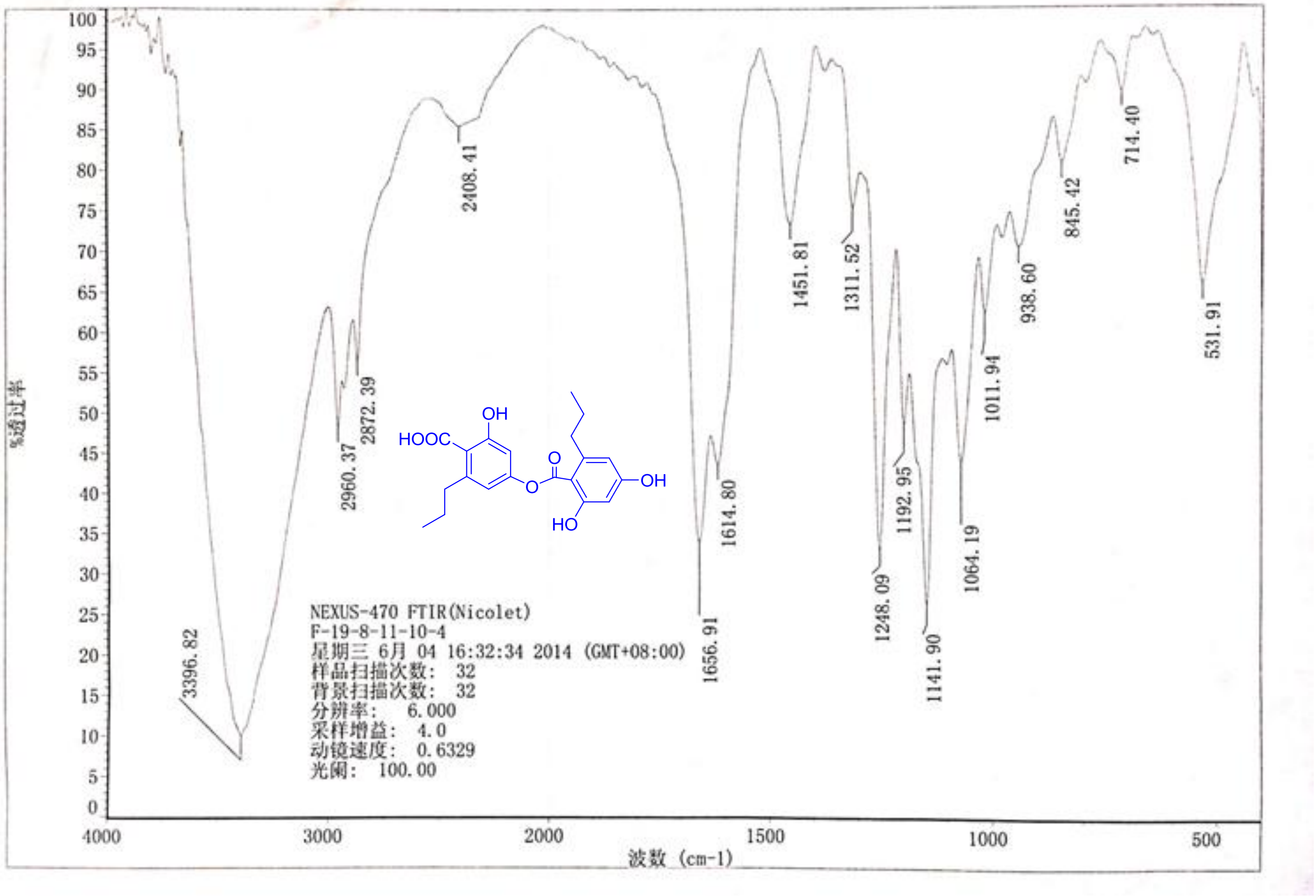

Figure S66. IR spectrum of compound **10**.

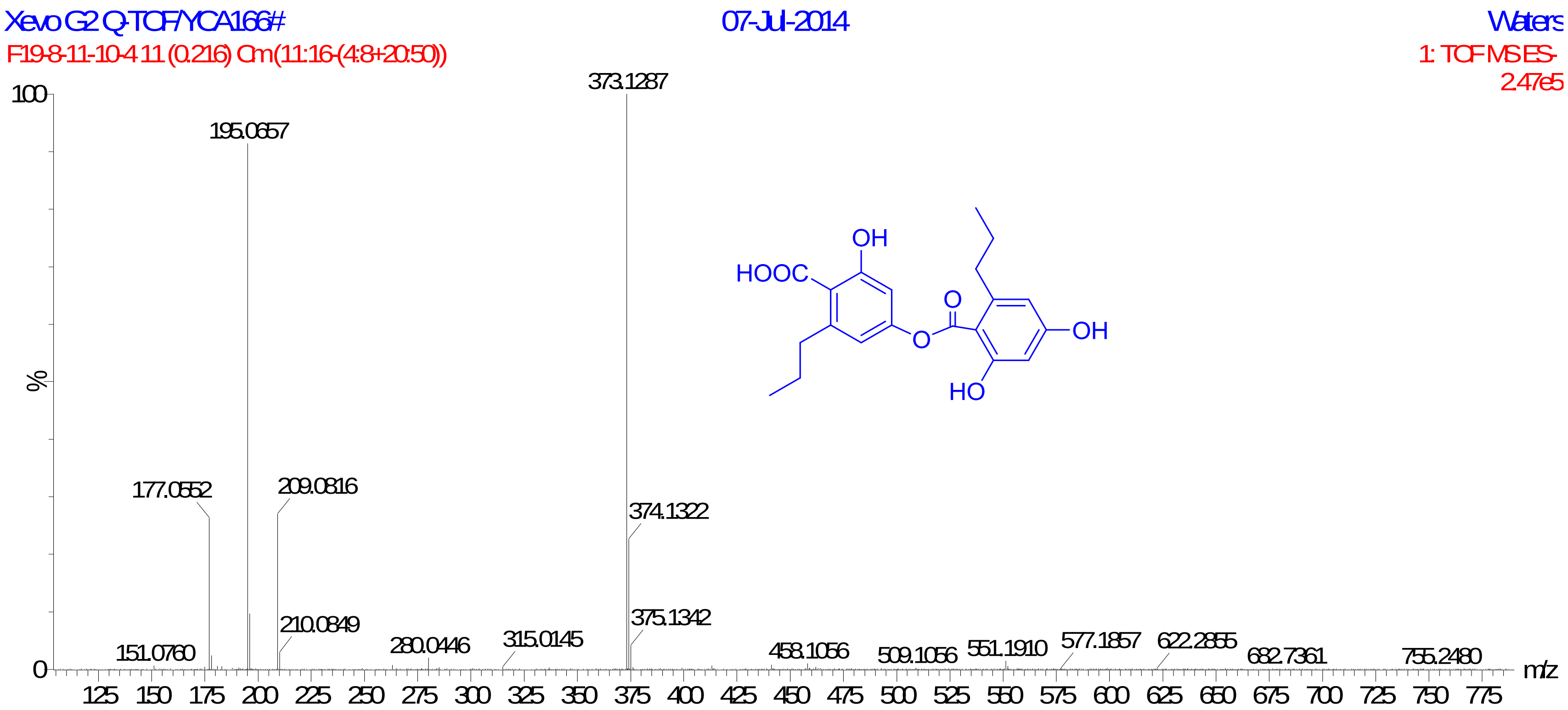

Figure S67. Negative mode HRESIMS data of compound **10**.

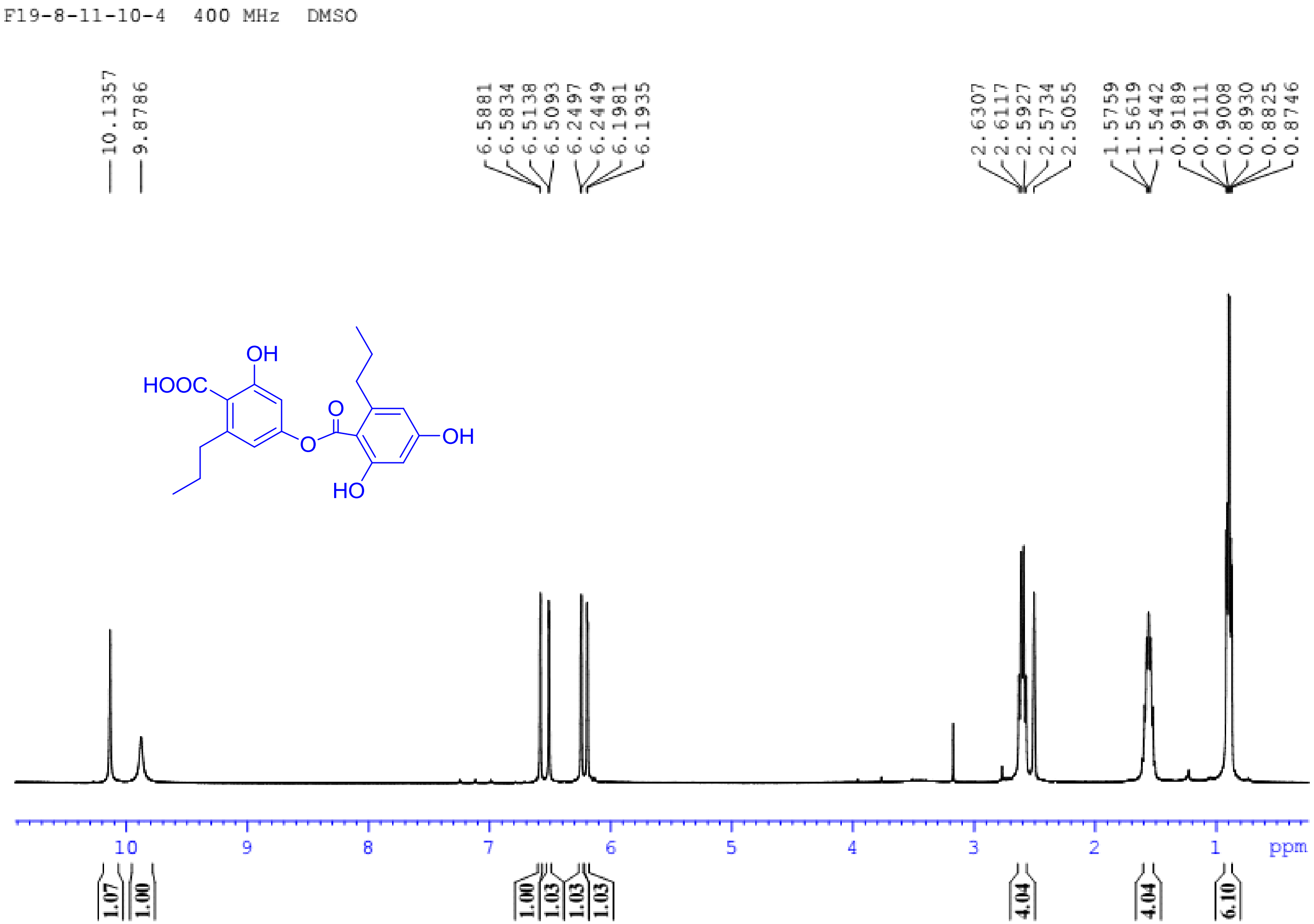

Figure S68. ^{1}H NMR spectrum of compound **10** in DMSO-d6 (400 MHz).

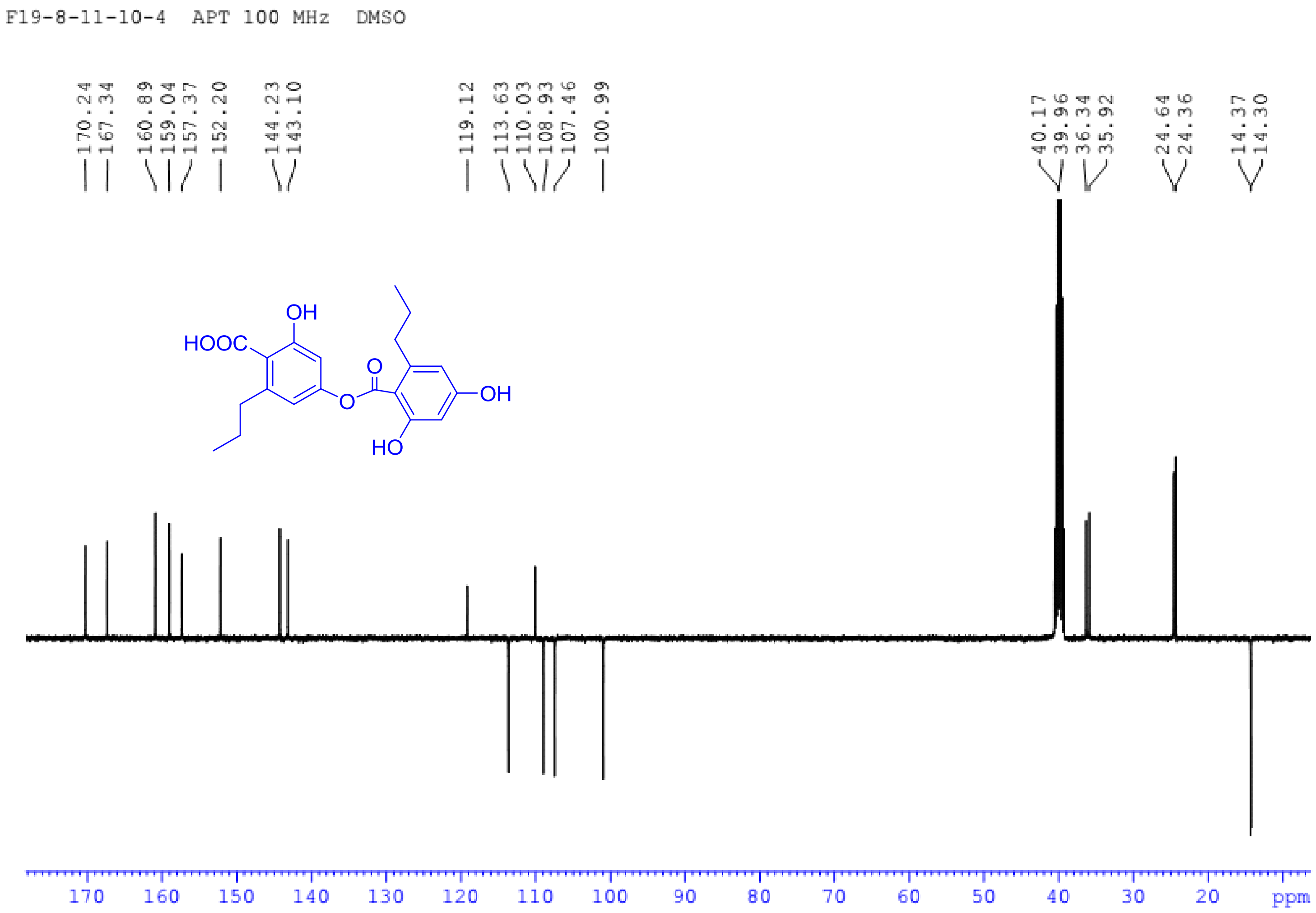

Figure S69. ^{13}C NMR spectrum of compound **10** in DMSO-d6 (100 MHz).

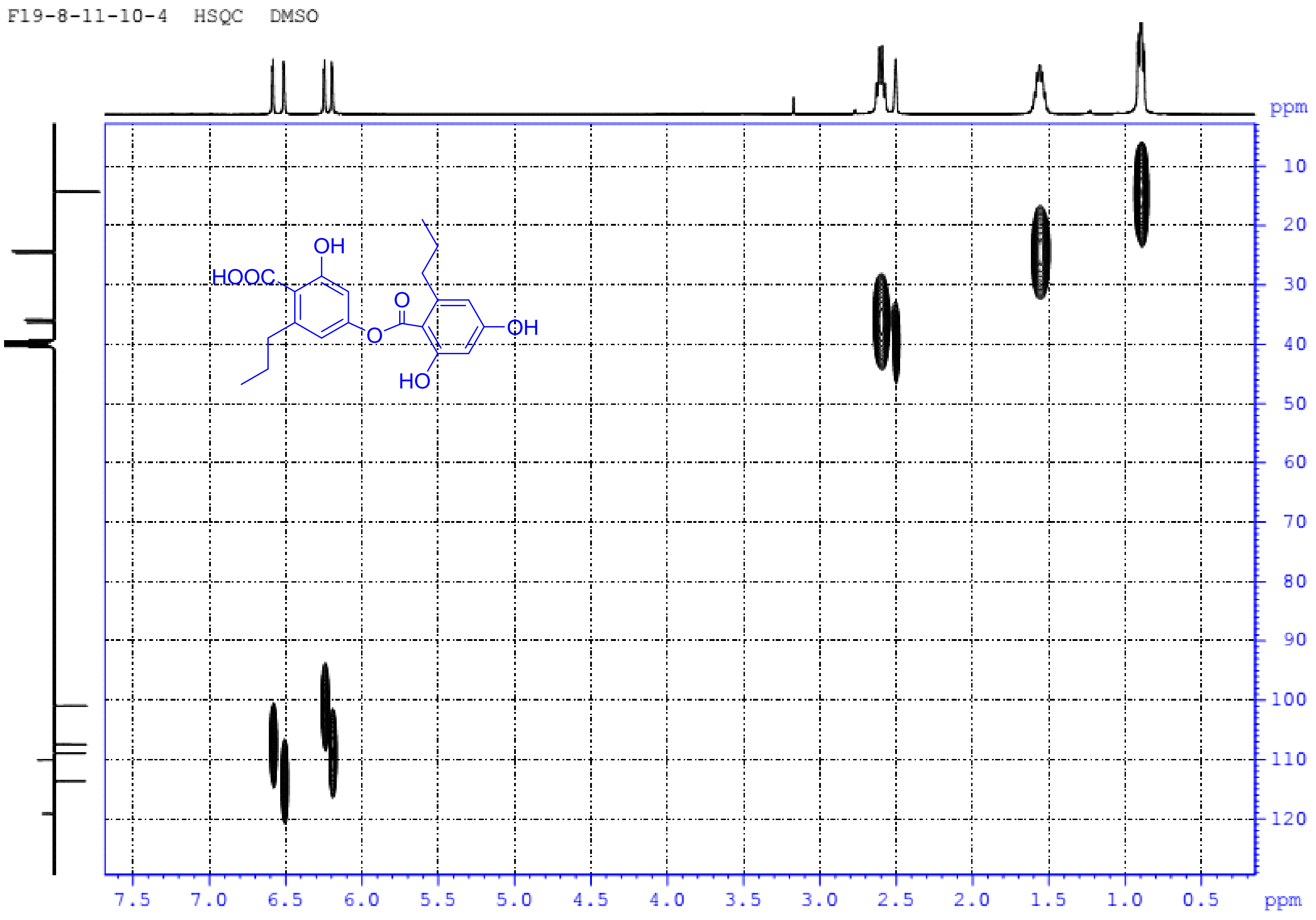

Figure S70. HSQC spectrum of compound **10** in DMSO-d6.

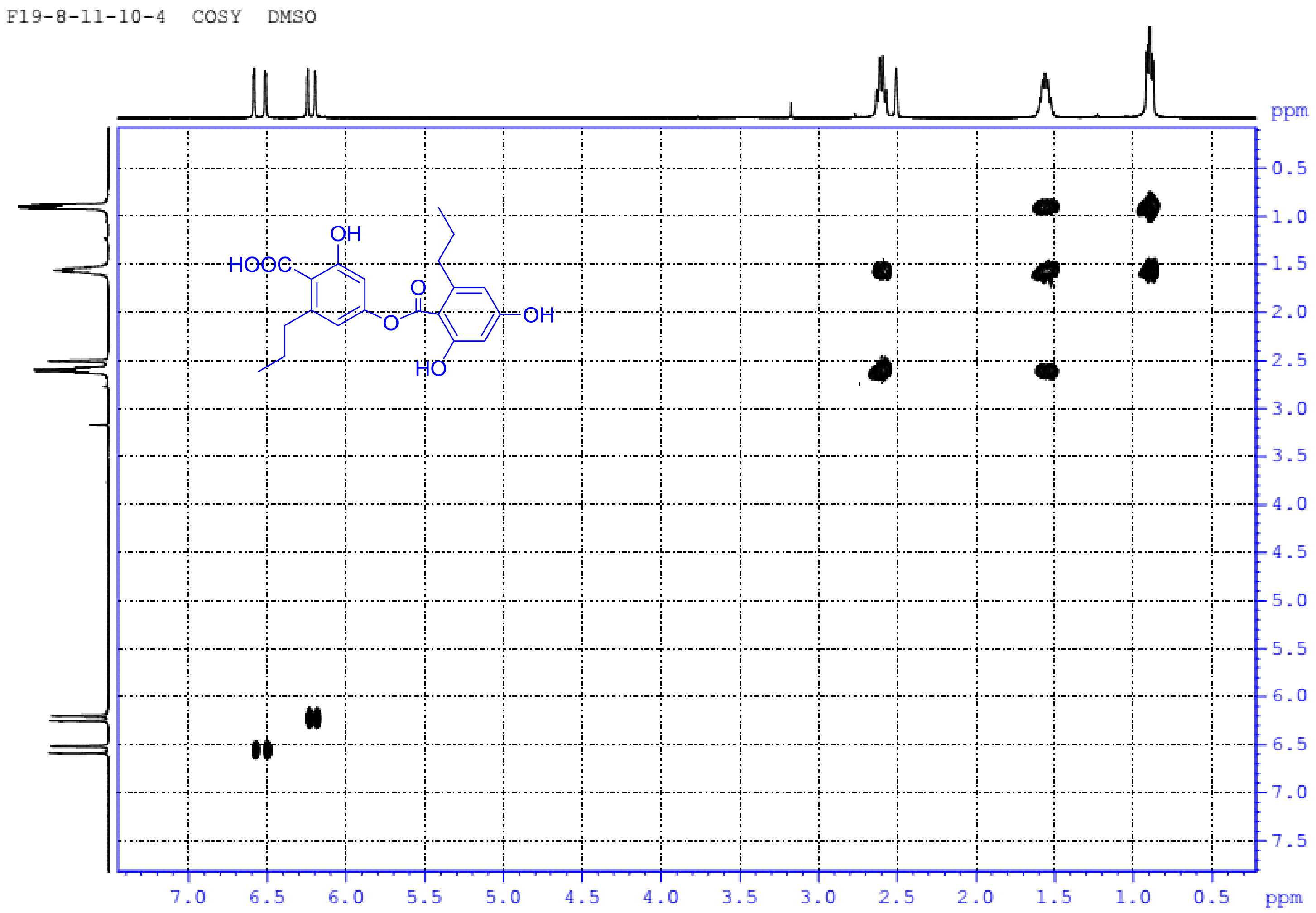

Figure S71. ^{1}H-^{1}H COSY spectrum of compound **10** in DMSO-d6.

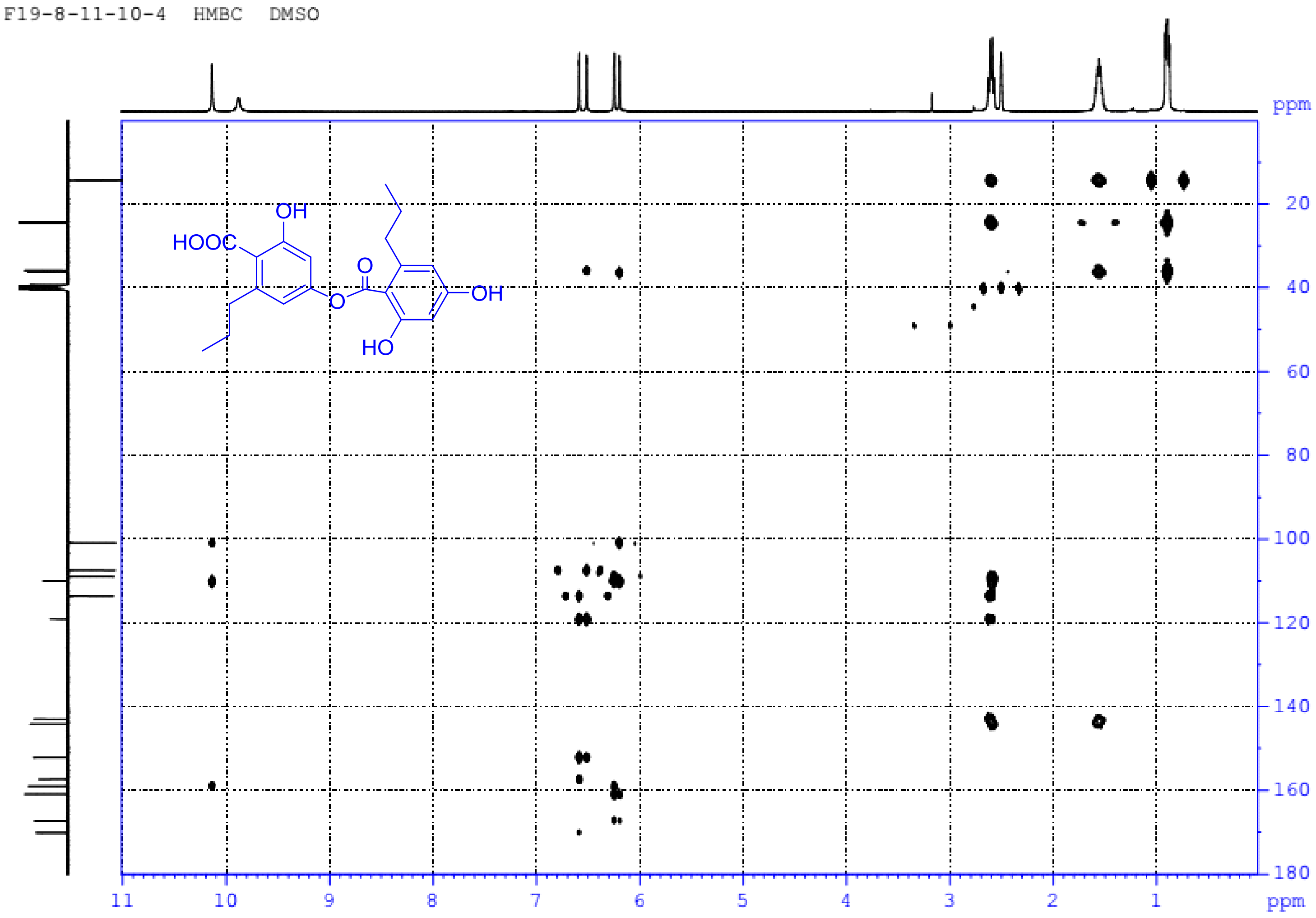

Figure S72. HMBC spectrum of compound **10** in DMSO-d6.

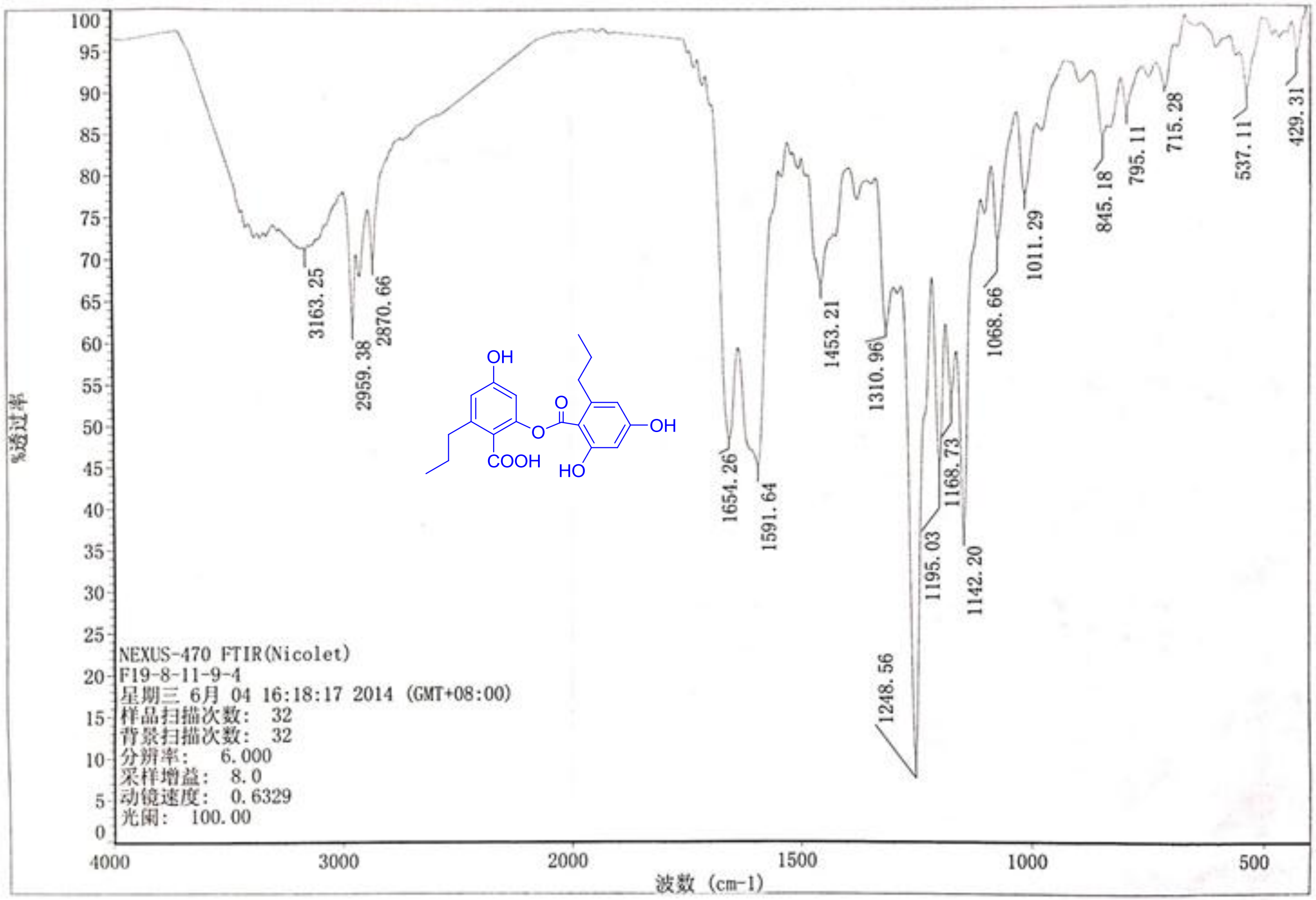

Figure S73. IR spectrum of compound **11**.

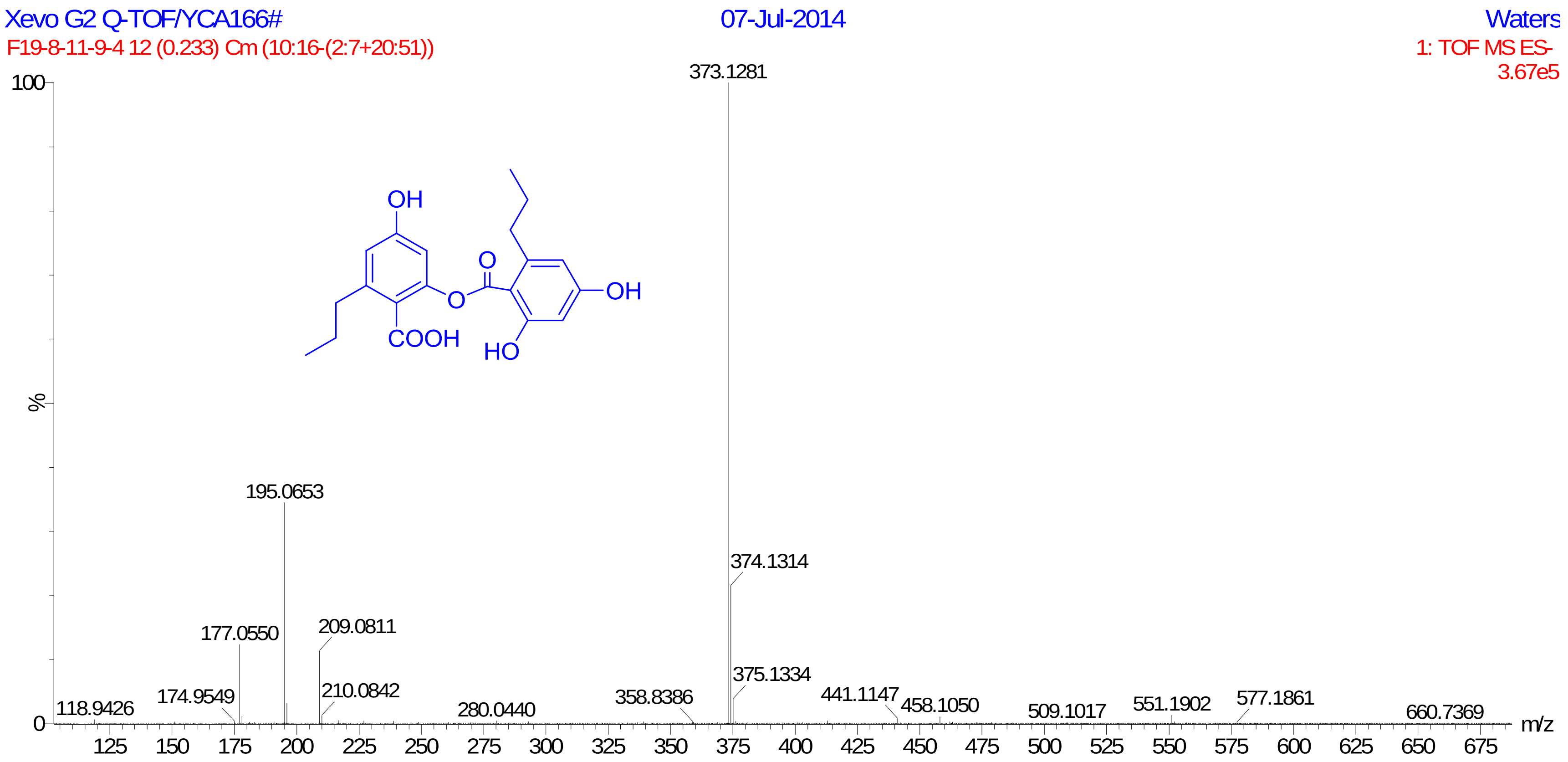

Figure S74. Negative mode HRESIMS data of compound **11**.

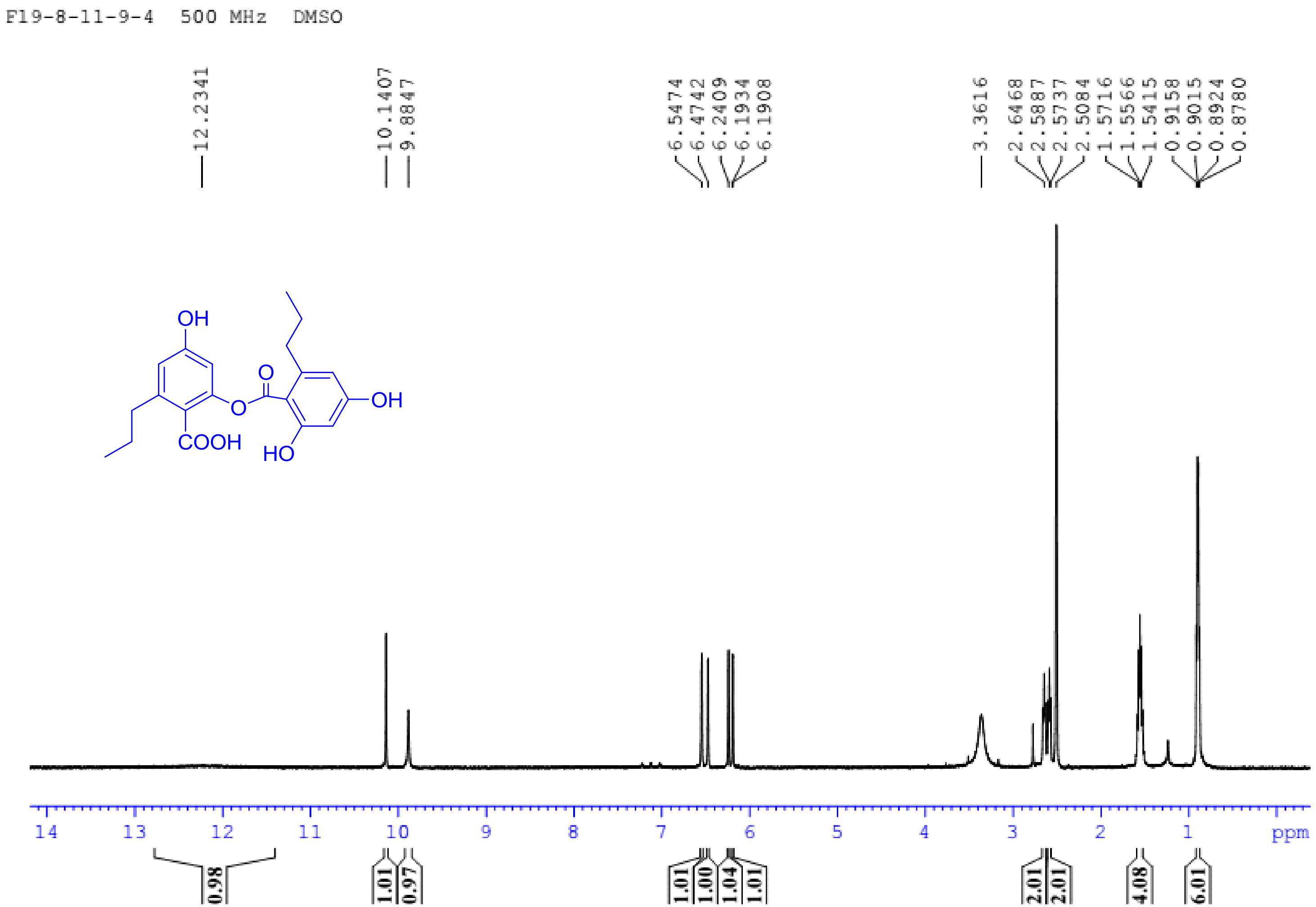

Figure S75. ^{1}H NMR spectrum of compound **11** in DMSO-d6 (500 MHz).

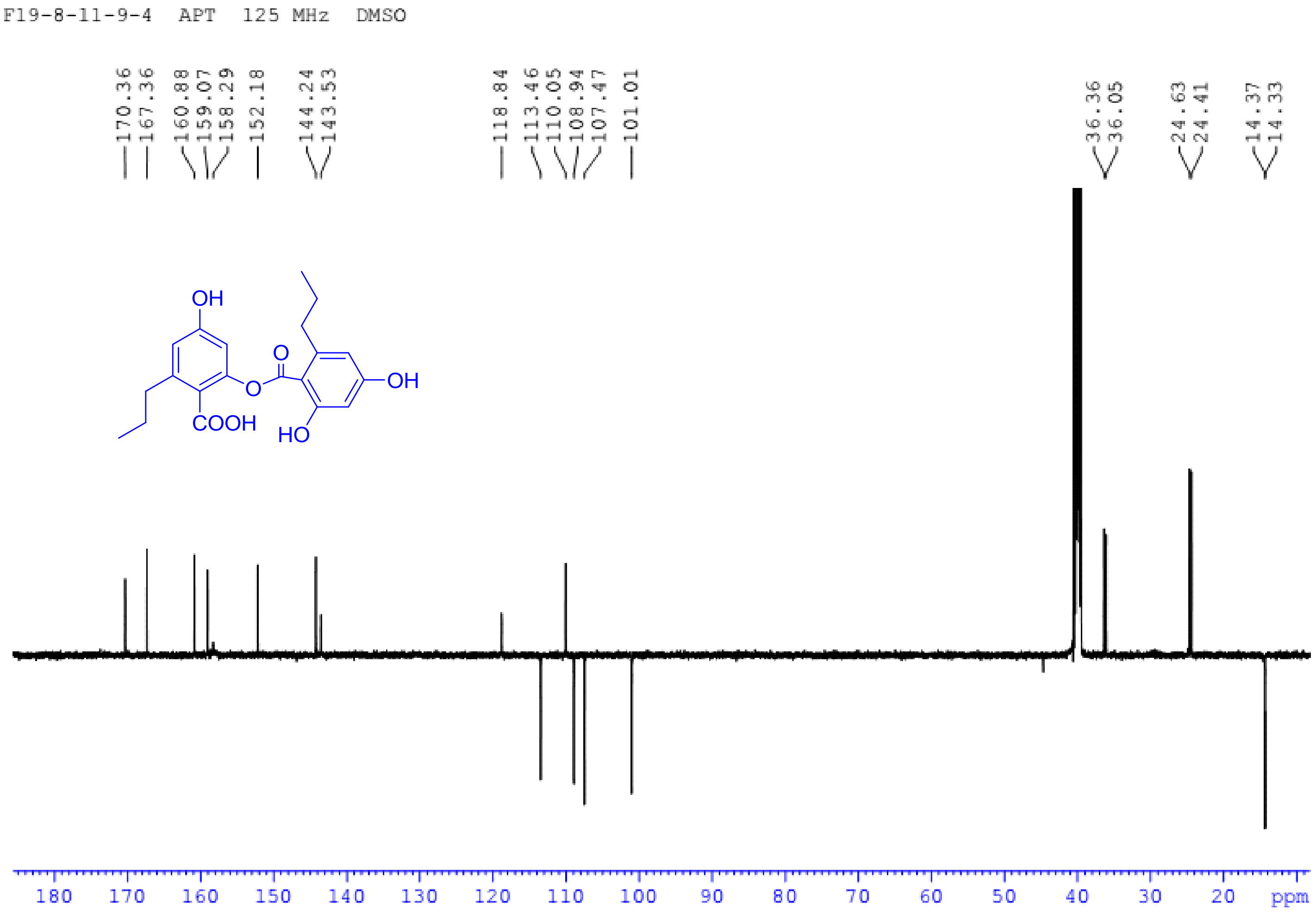

Figure S76. ^{13}C NMR spectrum of compound **11** in DMSO-d6 (100MHz).

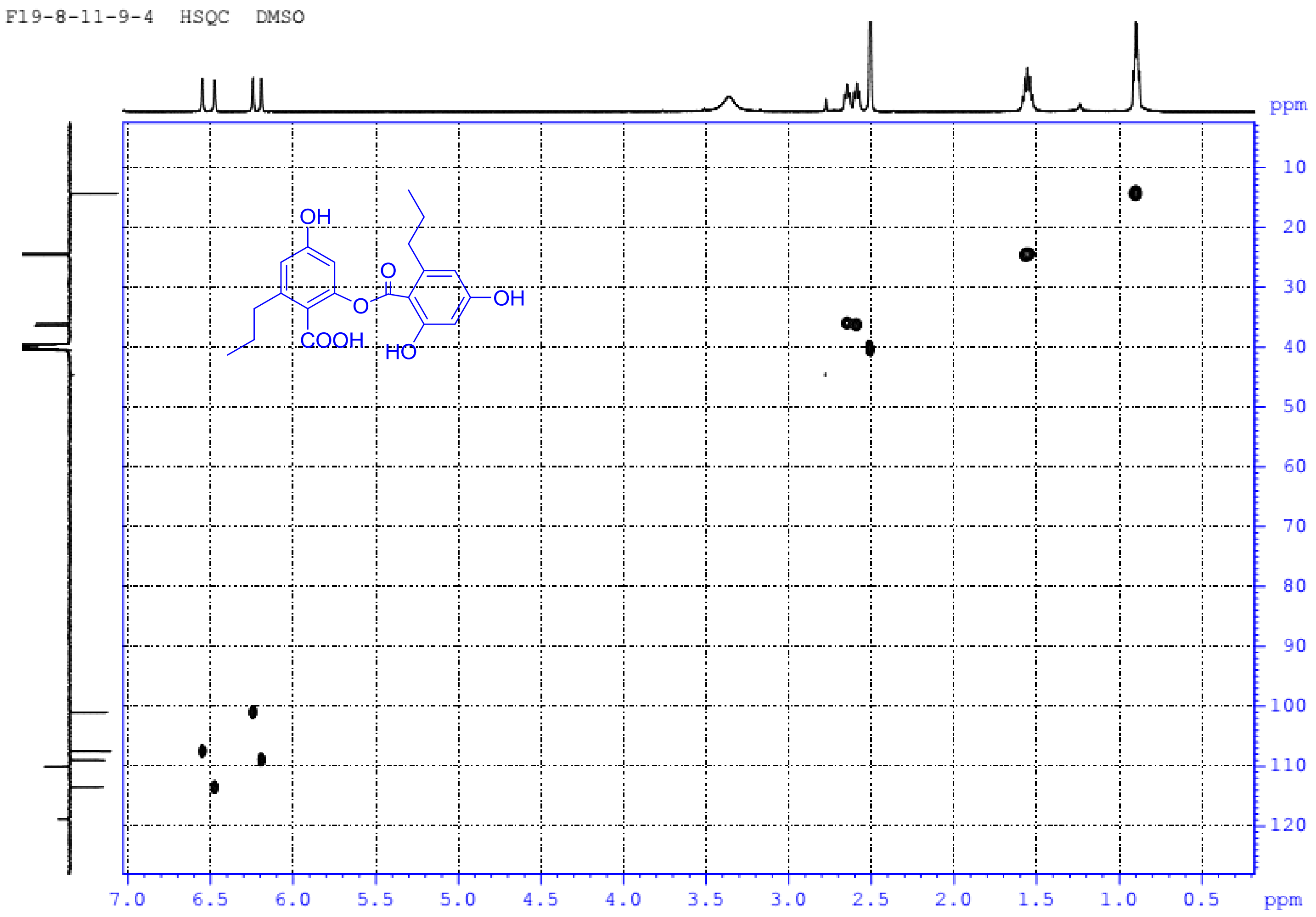

Figure S77. HSQC spectrum of compound **11** in DMSO-d6.

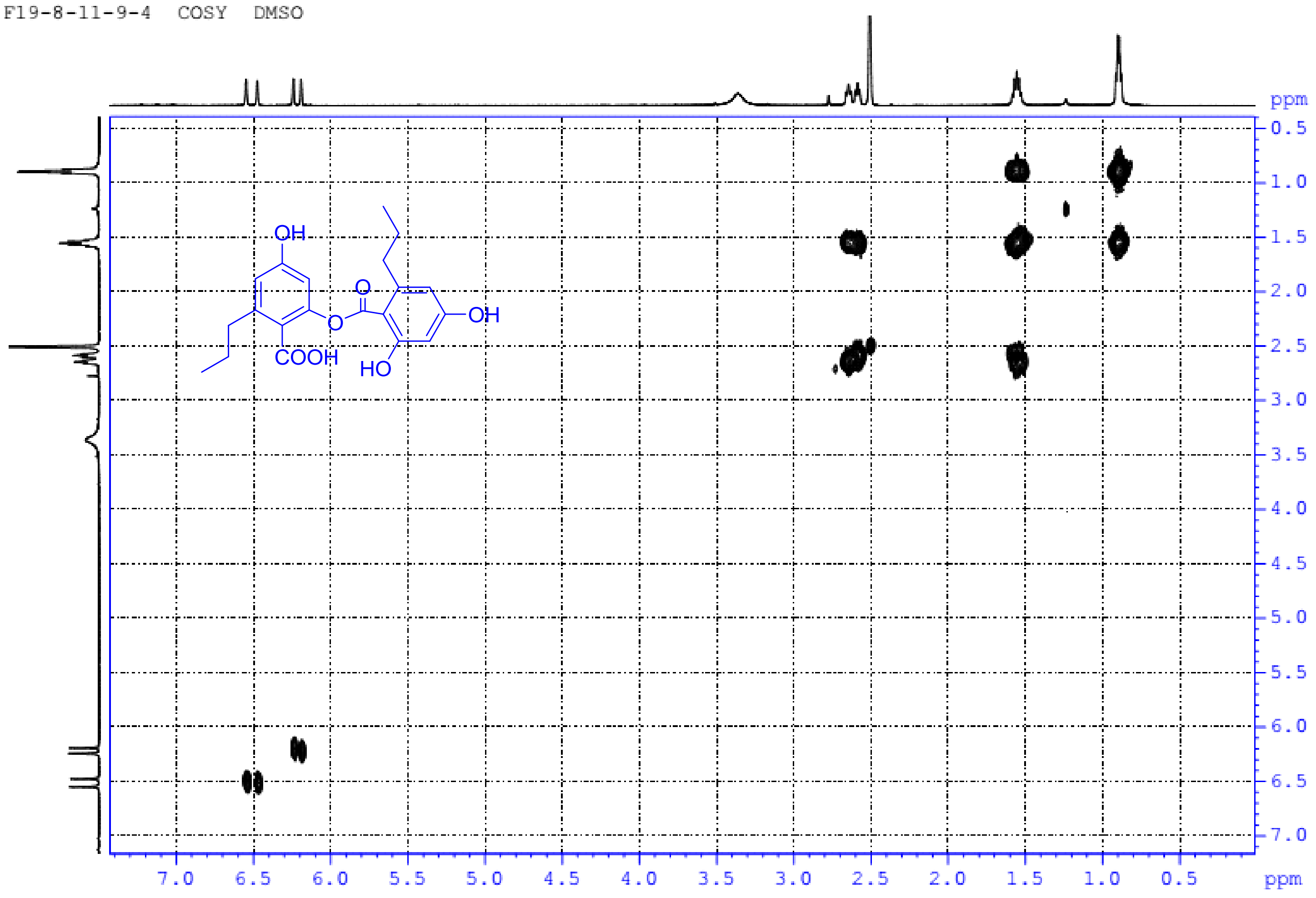

Figure S78. ^{1}H-^{1}H COSY spectrum of compound **11** in DMSO-d6.

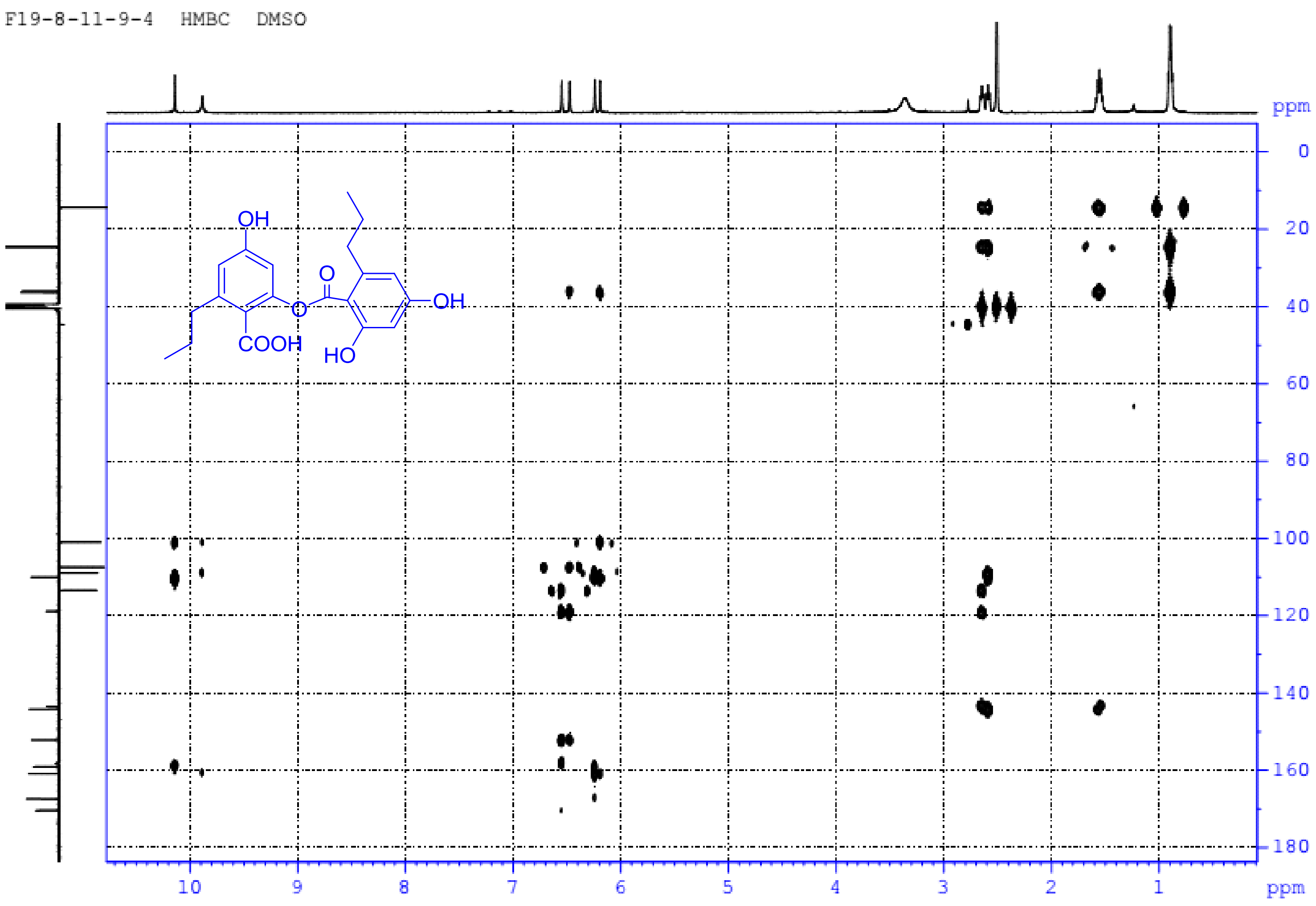

Figure S79. HMBC spectrum of compound **11** in DMSO-d6.

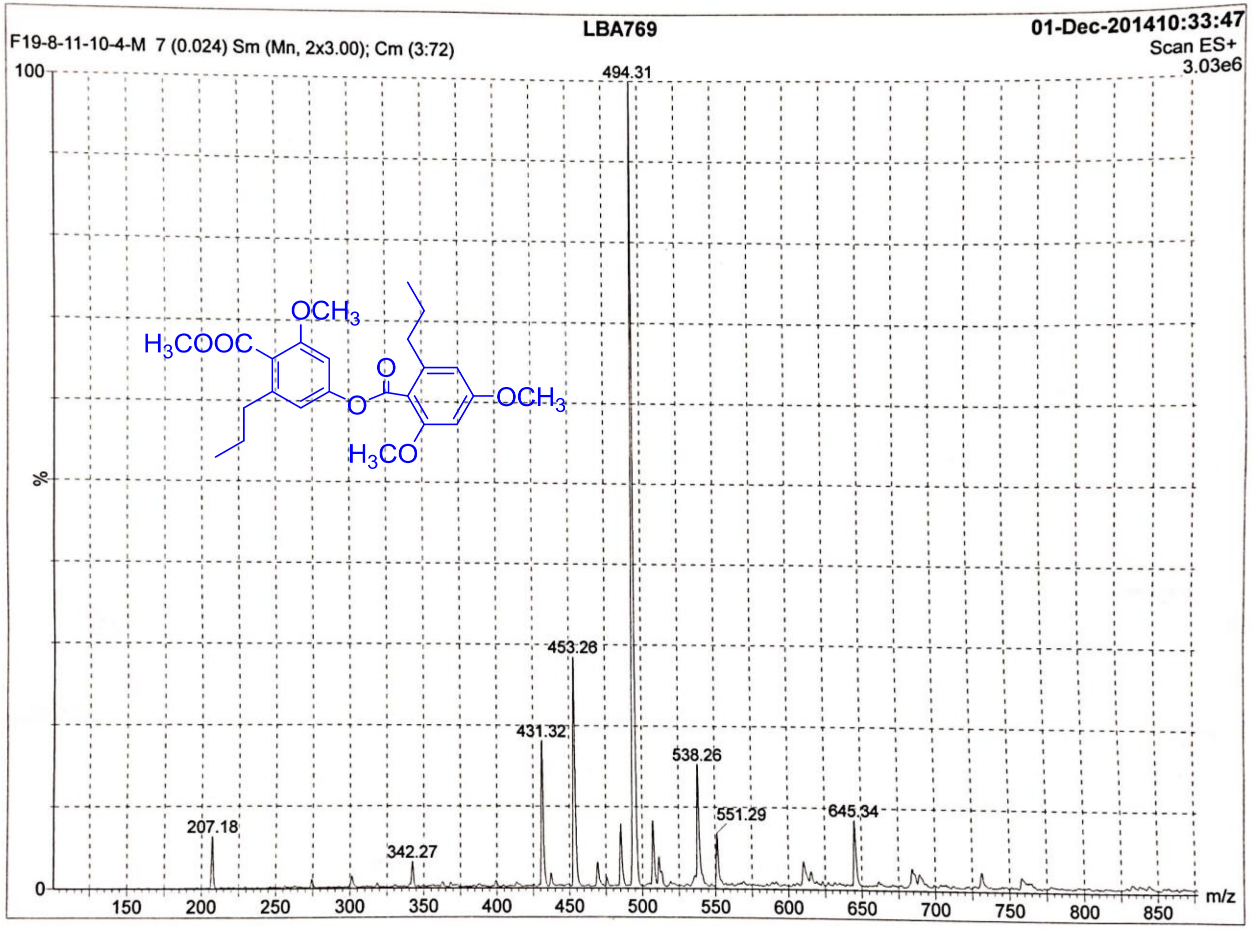

Figure S80. Negative mode ESIMS data of compound **10a**.

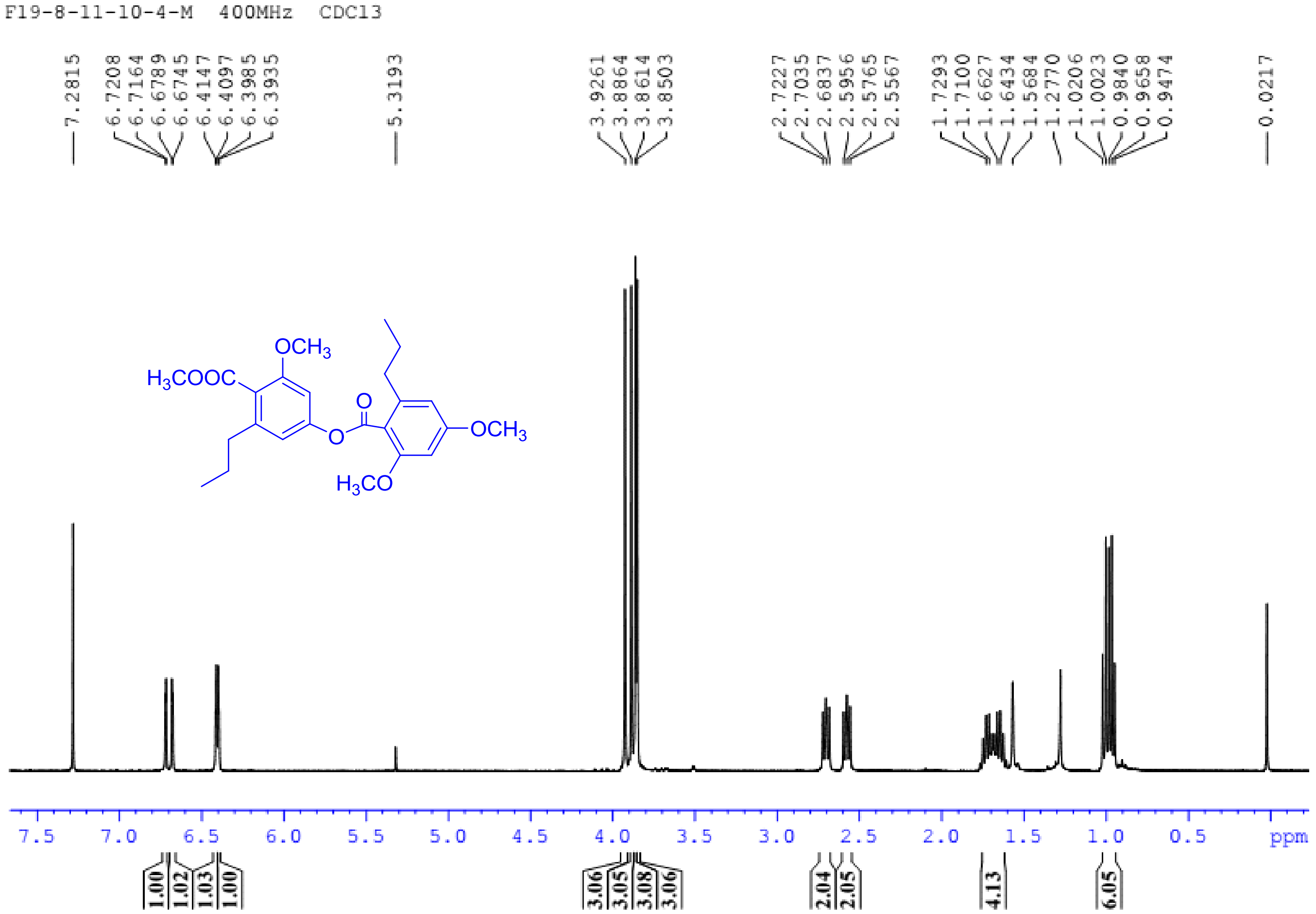

Figure S81. ^{1}H NMR spectrum of compound **10a** in $CDCl_3$ (400 MHz).

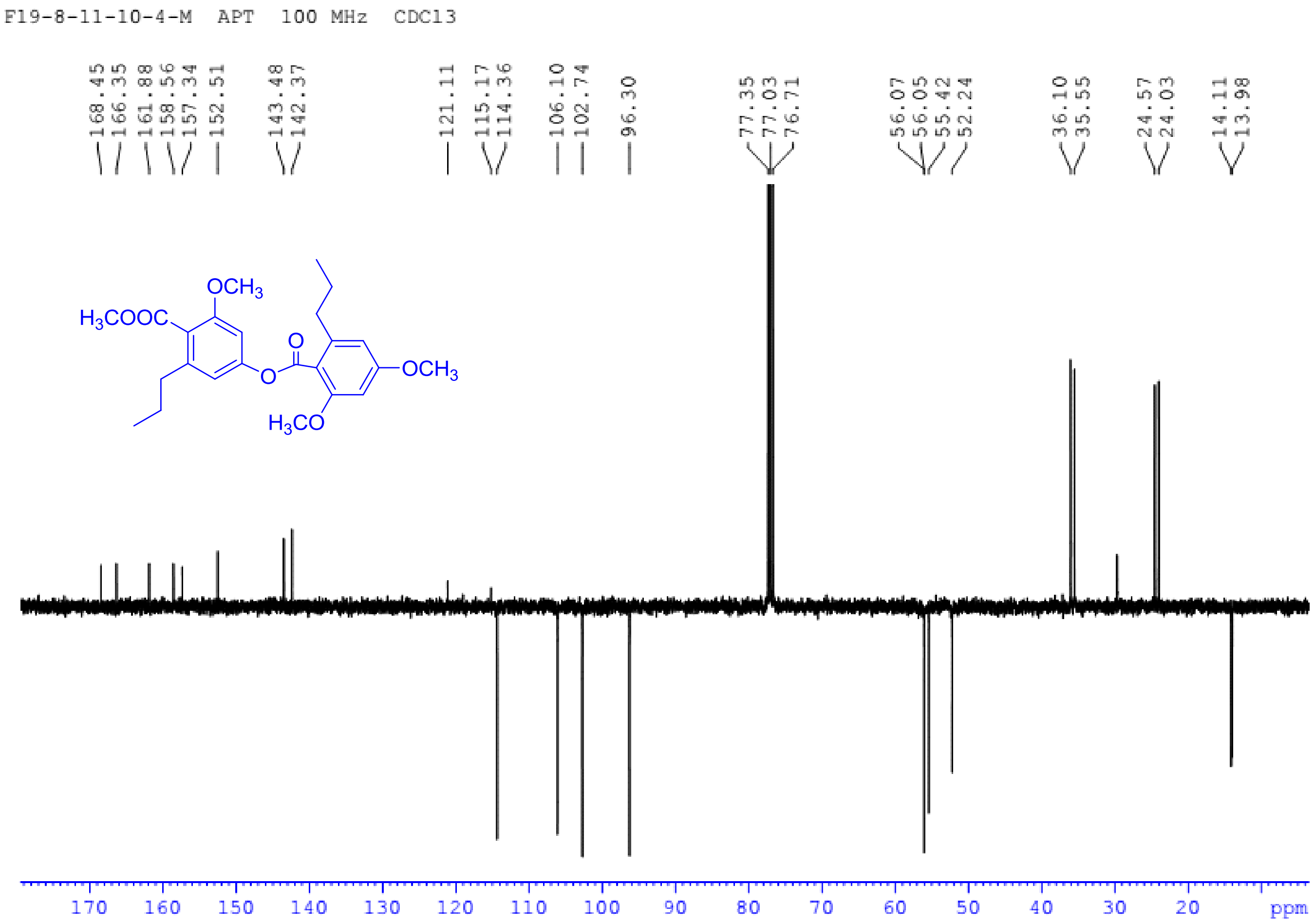

Figure S82. ^{13}C NMR spectrum of compound **10a** in $CDCl_3$ (100 MHz).

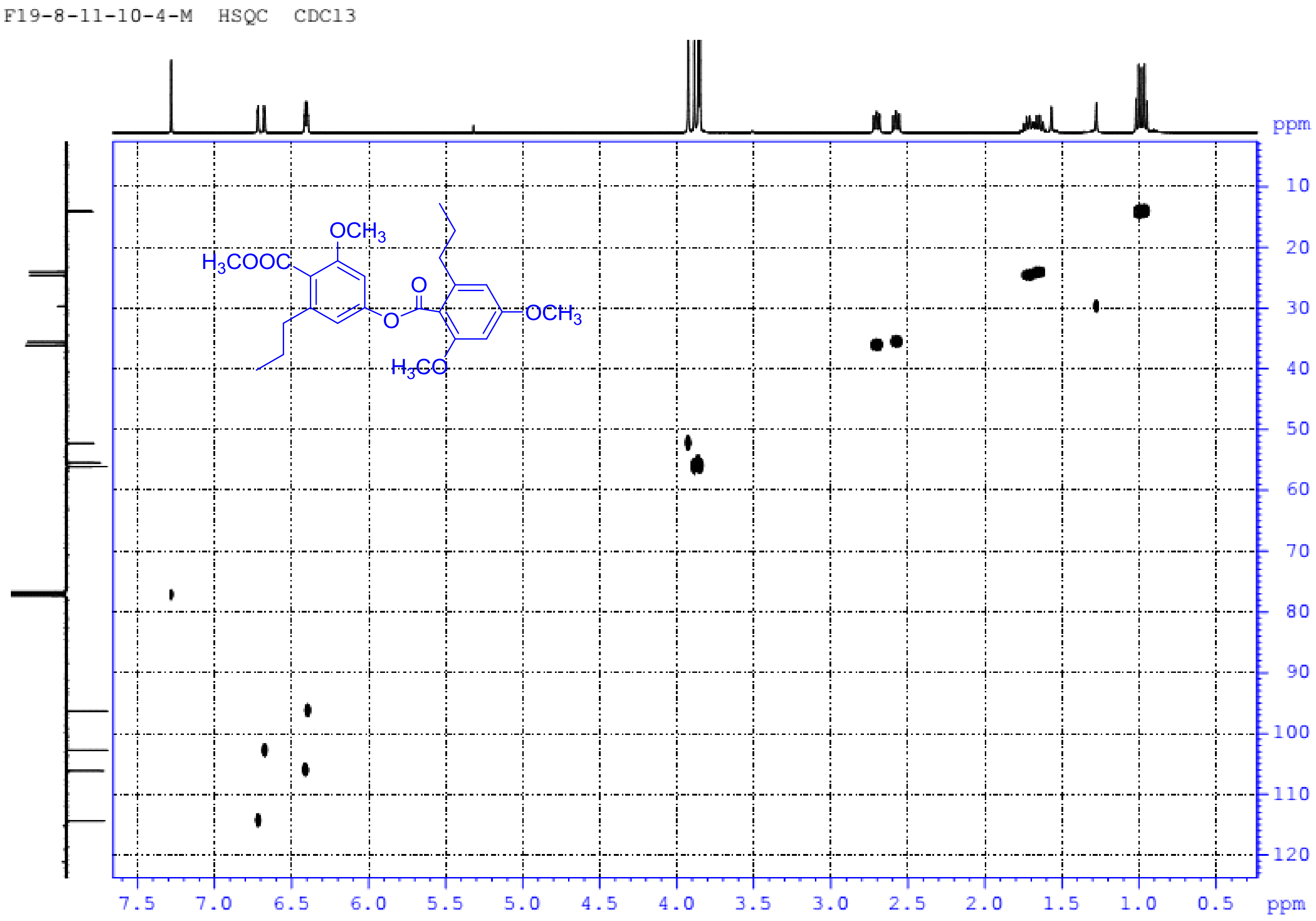

Figure S83. HSQC spectrum of compound **10a** in $CDCl_3$.

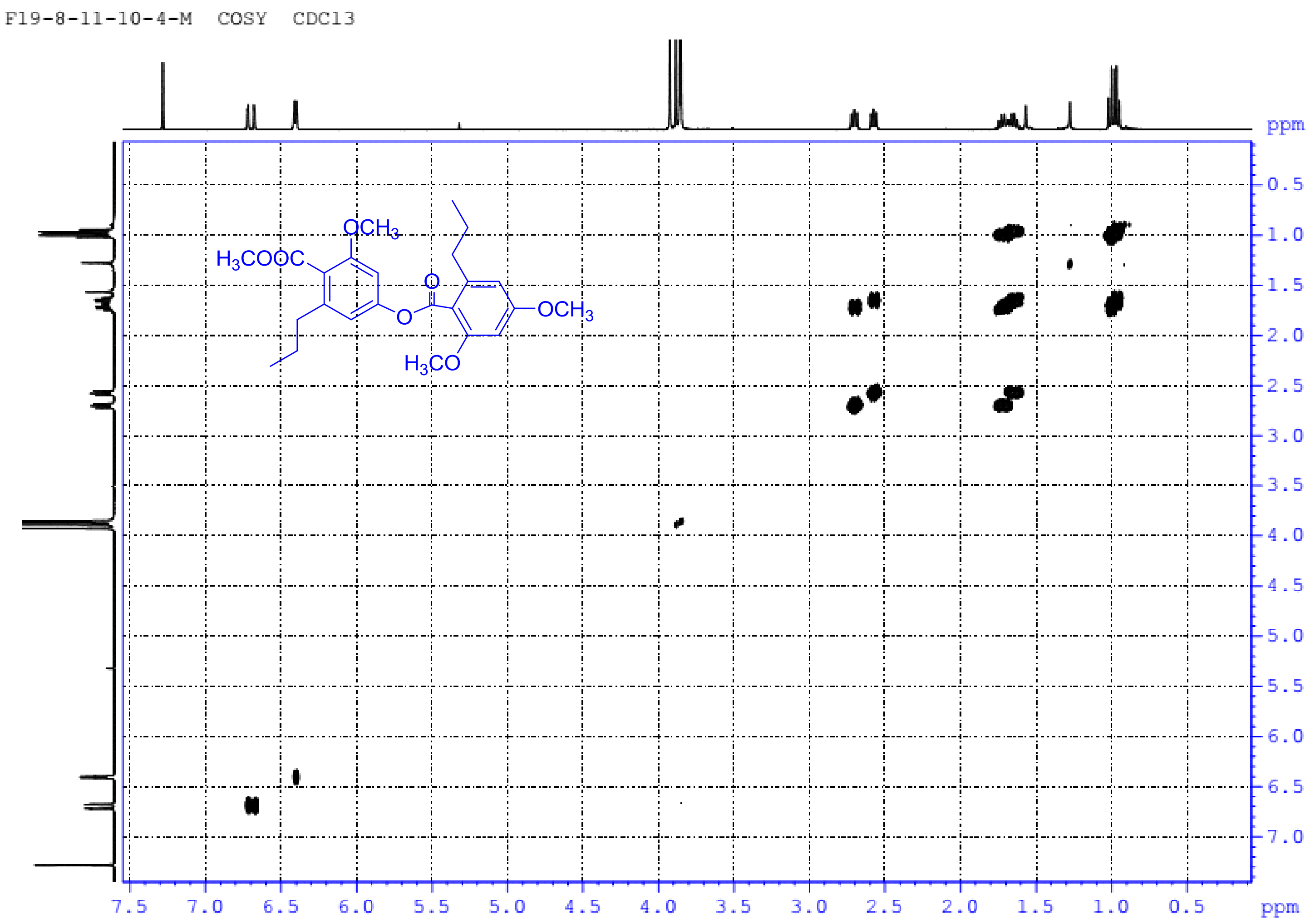

Figure S84. ^{1}H-^{1}H COSY spectrum of compound **10a** in $CDCl_3$.

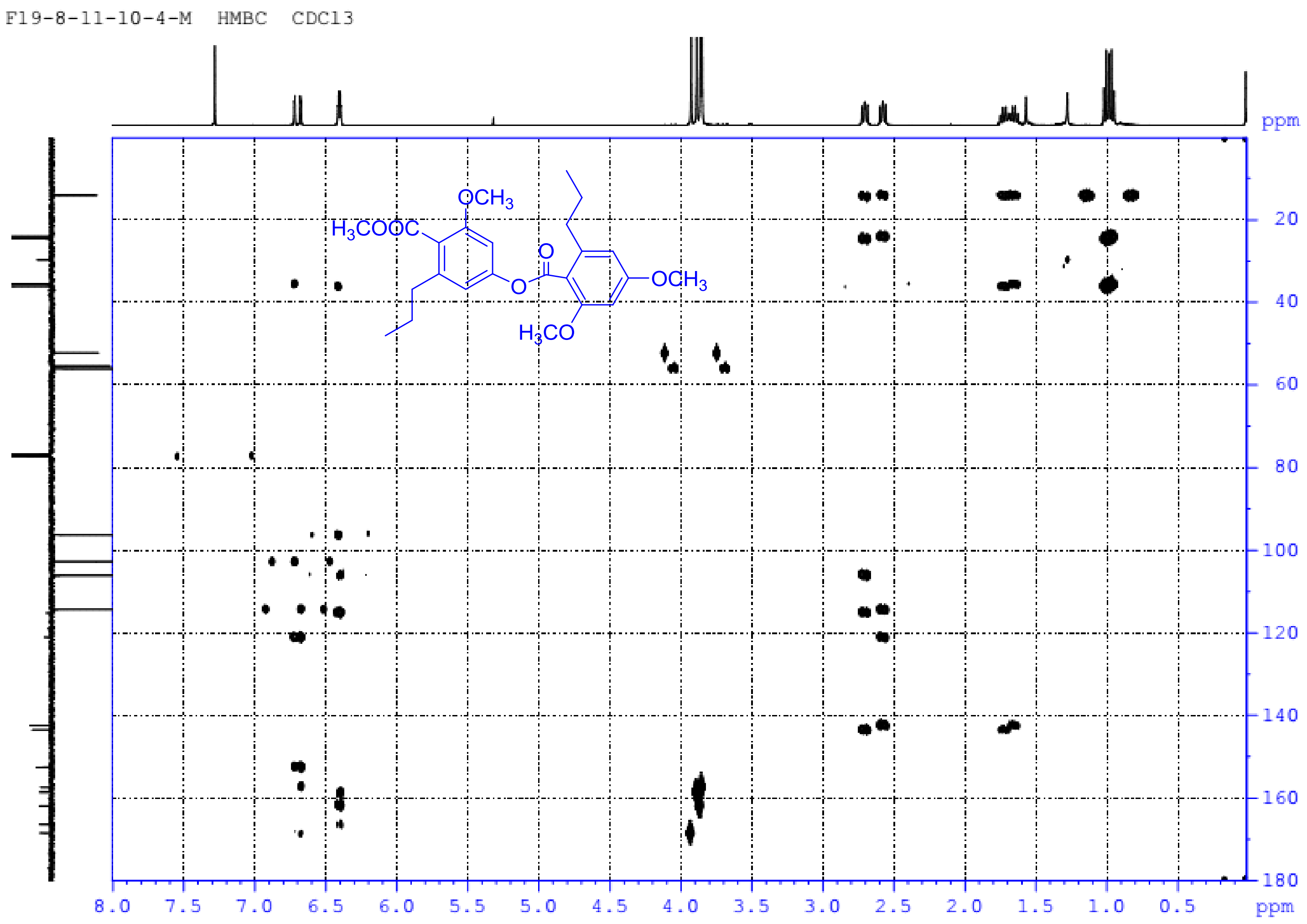

Figure S85. HMBC spectrum of compound **10a** in $CDCl_3$.

© 2015 by the authors; licensee MDPI, Basel, Switzerland. This article is an open access article distributed under the terms and conditions of the Creative Commons Attribution license (http://creativecommons.org/licenses/by/4.0/).
